# Ibrutinib enhances the bias of T cell responses towards staphylococcal superantigens sustaining inflammation in chronic lymphocytic leukaemia

**DOI:** 10.3389/fimmu.2025.1531059

**Published:** 2025-03-26

**Authors:** Fisal Tantoush, David Allsup, Leigh Naylor-Adamson, Frank Voncken, Stefano Caserta

**Affiliations:** ^1^ Biomedical Institute for Multimorbidity, Centre for Biomedicine, Hull York Medical School, Faculty of Health Sciences, University of Hull, Hull, United Kingdom; ^2^ Department of Haematology, Castle Hill Hospital, Hull University Teaching Hospital NHS Trust, Hull, United Kingdom

**Keywords:** chronic lymphocytic leukaemia, superantigens, Staphylococcus aureus, pseudo-exhaustion, chronic inflammation, ibrutinib, TSST-1, SEB

## Abstract

Chronic lymphocytic leukaemia (CLL) is an uncurable haematological malignancy and is associated with significant infection morbidity. Bruton’s tyrosine-kinase inhibitors (*e.g.*, ibrutinib) have improved disease outcomes, but severe infections and poor immunization responses afflict patients. Recently, carriage of the endemic *Staphylococcus aureus* (SA) was associated with lymphocytosis and decreased survival in CLL patients. We then hypothesized that exposure to staphylococcal superantigens (SAgs), known to promote hyper-inflammatory responses, impairs immunity and increases severe infection risk in CLL patients. Herein, we evaluate the reactivity of T cells and CLL cells to SA SAgs, in cultures derived from ibrutinib-treated and untreated CLL patients. We found that ibrutinib-treated patients had less naive CD8+ T cells (p=0.0348), more checkpoint receptor (TIM-3) expression in memory T cells (p<0.0001), and lower IFNγ/cytokine responses in patient T cells (p≤0.0298). Exposure to SA SAg further increased the accumulation of memory T cells with an exhaustion-phenotype, preferentially in cultures derived from ibrutinib-treated patients (p≤0.0350). Nevertheless, staphylococcal SAgs could not induce regulatory T cells from CLL patients inasmuch as healthy donors (p≤0.0461) and this was associated with accumulation of inflammatory T cells. Significantly, SAg-exposure enhanced inflammatory activation of CLL tumour cells, which acquired CD38, CD40, CD86, while downregulating CD27 (p≤0.005), even in cultures from ibrutinib-treated CLL patients. Thus, we suggest that environmental SAg-exposure promotes the accumulation of pseudo-exhausted T cells, which induce/sustain tumour cell activation, not counteracted by ibrutinib. Our study critically helps understand the chronic inflammatory milieu in CLL patients, with implications for infection morbidity, disease aetiology and future interventions.

## 1 Introduction

Chronic lymphocytic leukaemia (CLL) is the most frequent blood cancer (1–3) characterised by B-cell malignant transformation and expansion, with heterogenous mutational profiles (*e.g.*, mutated *vs* unmutated *Ighv* and mutations of *Tp53*, *Atm*, *Sf3b1*, *Notch1*, *Birc3*, etc.) reported in patients (4–9). CLL aetiology still remains unknown, with exposure to autoantigens (4) as well as environmental pathogens (most frequently of viral origin) implicated as a possible cause (10, 11).

CLL patients suffer from recurrent infections, often caused by endemic bacteria which account for up to 50% of CLL deaths (12). Sepsis is the commonest cause of mortality in CLL, killing ~20% of patients and accounting for 13% of CLL-related hospital admissions (13).

CLL patients with infectious co-morbidities are at a significantly higher risk of death, pointing at immune failure (14). Relevantly, chronic infections often promote T cell exhaustion characterised by upregulation of checkpoint receptor inhibitory pathways (15, 16) that curtail long-term protection against cancer and pathogens. In CLL, CD8+ T cells acquire an exhaustion-phenotype with high expression of checkpoint receptors PD-1, CD160, and CD244. However, these do not fully exhaust cytokine responses, hence it was previously suggested that CLL may be characterised by a pseudo-exhaustion state, independent of infection with the endemic virus, cytomegalovirus (17) [CMV; a known promoter of T cell inflationary cytokine responses (18)]. However, the drivers or causes behind the pseudo-exhaustion of T cells observed in CLL patients remain unknown.

Up to 40% of untreated CLL patients can carry *Staphylococcus aureus* (SA) in the upper respiratory tract, three-fold higher than that found in healthy subjects and associated with increased lymphocytosis, PD-1 expression and reduced survival (19, 20). Thus, while cancer primary immunosuppressive mechanisms may trigger exhaustion in CLL patients, we hypothesised that exposure to endemic pathogens may escalate this further.

Bruton’s tyrosine-kinase inhibitors (BTKi), including ibrutinib and second generation BTKis (*e.g.*, acalabrutinib, zanubrutinib) have significantly enhanced CLL patient survival (21–25). However, ibrutinib-treated CLL patients suffer from recurrent/severe infections (including sepsis), especially in relapse (26) and fail to (or minimally) respond to prophylactic vaccinations (27–31). Whilst ibrutinib can exert a toxic effect against CLL-cells, this drug can impact and skew T cell differentiation via off-target inhibition of Itk (32), a Tec kinase involved in T-cell-receptor (TCR)-signalling (33). In their seminal work, Dubovsky et al. (32) elegantly showed that ibrutinib covalently binds to Tec/Itk, specifically in T cells (including those from CLL patients). The authors showed that ibrutinib inhibits responses to the archetypal TCR stimulants (anti-CD3 and anti-CD28 crosslinking stimulatory antibodies, αCD3/28) in T cell cultures derived from healthy donors (32). Within 8 days of a first *de novo* administration, ibrutinib was found to hamper responses in T cells from CLL patients, curtailing T cell function and differentiation (32). Despite the inhibitory role of ibrutinib on early TCR-signals, recent studies support that ibrutinib long-term treatment restores T cell numbers, subset distribution and function *in vivo* (34–37), especially compared to immunosuppressive chemotherapy (38). Ibrutinib may even rescue T cells from exhaustion (34, 35, 37, 39–41) and/or senescence (36), emerging as a potential synergistic treatment to boost T cells in novel CLL-targeted immunotherapy (39, 42–44). Moreover, ibrutinib decreases the frequency of suppressive, regulatory T cells (Tregs) in CLL patients (35, 45), which should facilitate effector/conventional T cells fight cancer and infections (46). However, such improvement of T cell function/numbers contrasts with the increased vulnerability to infection seen in CLL patients, especially those treated with ibrutinib (47–51) [and, to a lower extent, novel BTKis (23, 52–55)].

Given the recent evidence that SA carriage is high in CLL patients (19) and that staphylococcal superantigen (SAg) toxins (56) hijack T cell activation and drive pro-inflammatory cytokine release, critical for severe infection/sepsis (57–60), we postulated that environmental SAg-exposure compromises immunity in CLL. Consistent with this, we show that T cells from CLL patients (including, ibrutinib-treated) preferentially develop exhaustion-like, central-memory phenotype, whilst preserving inflammatory cytokine secretion, under the strain of chronic SAg-exposure, *in vitro*. We provide definitive evidence that T cells from CLL patients mount defective Treg responses to staphylococcal SAgs, and this associates with higher inflammatory responses including the activation of tumour cells. Together, our results point at exposure to endemic SA as a likely driver of chronic T/CLL-cell-driven inflammation, potentially promoting cancer progression and heightening infection risk in CLL patients. Our results have then implications for future interventions leading to new diagnostic/prognostic applications and guidance to help manage infections in CLL patients and reduce sepsis occurrence, while bringing innovative understanding of CLL development.

## 2 Materials and methods

### 2.1 Ethical approvals, patients and healthy donors

Blood from CLL patients was taken under Local Research Ethics Committee approval (08/H1304/35), and used under the University of Hull (UoH) ethical reference, FHS184. We recruited CLL patients (n=23, **Table 1**) either untreated (n=13) or treated with ibrutinib [n=10; daily dose: 420 mg, except two patients receiving 140 mg (61)]. Blood collection from healthy donors (HDs, n=8) was approved by the UoH Faculty of Health Science Research Ethics Committee (FHS70).

**Table 1:** CLL patient characteristics.

Variable	CLL (n=23)	Untreated CLL (n=13)	Ibrutinib-treated CLL (n=10)	Chi-square p value
**Age (years)**	73 (60-89)	73.08 (60-89)	72.80 (62-81)	0.93
Gender				0.66
Male	17 (74%)	9 (53%)	8 (47%)	
Female	6 (26%)	4 (67%)	2 (33%)	
F:M ratio		4:9 (30%)	2:8 (20%)	
Binet stage at diagnosis:				0.75
A	15 (65%)	9 (60%)	6 (40%)	
B	3 (13%)	1 (33%)	2 (67%)	
C	5 (22%)	3 (60%)	2 (40%)	
Rai stage at diagnosis^§^:				0.13
0	10 (43%)	4 (40%)	6 (60%)	
I	5 (22%)	5 (100%)	0 (0%)	
II	3 (13%)	1 (33%)	2 (67%)	
IV	5 (22%)	3 (60%)	2 (40%)	

^§^no patient was classified in Rai stage III.

### 2.2 Isolation of peripheral blood mononuclear cells

Peripheral blood was collected into sterile Sodium-Heparin Vacutainers (BD Biosciences), diluted (1:1) with sterile Dulbecco′s Phosphate Buffered Saline (DPBS), and layered on top of Ficoll-Paque PLUS (GE Healthcare Life Sciences). After spinning (1000g; 30 minutes; at room temperature, RT; no acceleration/deceleration), peripheral blood mononuclear cells (PBMCs) were collected and washed in DPBS (300 g; 10 minutes, RT), twice. Cells were resuspended in RPMI 1640 media supplemented with 10% heat-deactivated foetal bovine serum (FBS), 1% penicillin/streptomycin and L-glutamine (Gibco), hereafter referred to as complete media. Viable cells were counted using 0.1% Trypan Blue exclusion assays.

### 2.3 Primary cultures

Functional grade, purified mouse anti-human CD3 (0.125 µg/ml; Clone OKT3, eBioscience) and CD28 (2 µg/ml; Clone CD28.6, eBioscience) monoclonal antibodies (mAbs) were coated on wells in DPBS (1 hour; 37°C), before washing excess mAb twice and seeding cells. Staphylococcal SAgs, SEB (Sigma) and TSST-1 (2.5 µg/ml, Toxin Technology) were added to cultures as indicated. Typically, 2x10^6^ freshly isolated PBMCs/condition were seeded in complete media, in 24-well plates, and incubated in a humidified incubator at 37°C, 5% CO_2_, for 5 days.

### 2.4 Immunophenotyping of T and B cells

Freshly isolated PBMCs (2x10^6^) (*ex-vivo*) or cultured cells were washed (377 g; 5 minutes; 4°C) and incubated with anti-FcR blocking mAb (0.5 µg/sample; BioLegend) for 15 minutes at 4°C. Thereafter, cells were surface-stained (20 minutes, 4°C), with the following fluorescently-labelled mAbs (from BioLegend, unless stated otherwise; clones indicated in brackets). For T cells: BV510-labeled anti-CD3 (UCHT1); PerCP-labelled anti-CD4 (OKT4); APC-H7-labeled anti-CD8 (SK1; BD); AF700-labeled anti-CD45RA (HI100); FITC-labeled anti-CCR7 (G043H7); PB-labelled anti-CD127 (A019D5); PE-labelled anti-KLRG1 (2F1/KLRG1); APC-labelled anti-CTLA-4 (BNI3); PE/Dazzle 594-labeled anti-LAG3 (11C3C65); BV605-labeled anti-TIM-3 (F38-2E2); and PE/Cy7-labeled anti-PD-1 (J43; eBioscience). For B cells: BV510-labeled anti-CD3; PB-labelled anti-CD127; APC-H7-labeled anti-CD19 (HIB19; BD); AF700-labeled anti-CD40 (5C3); FITC-labeled anti-HLA-DR (Tü39); PE-labelled anti-CD20 (2H7); PerCP-Cy5-5-labeled anti-CD24 (ML5); APC-labelled anti-CD38 (HIT2); BV605-labeled anti-CD27 (O323) and PE/Cy7-labled anti-CD86 (BU63). Excess mAb was washed by centrifugation after adding cold PBS, 2% FBS, 0.2% NaN_3_ (FACS buffer, 377 g, 5 minutes, 4°C). After decanting supernatants, cells were fixed with 1% BD Fix/Lyse buffer (10 minutes, BD). Fixed cells were washed twice (FACS buffer, 500 g, 6 minutes, 4°C) before acquisition.

### 2.5 Intracellular cytokine staining

As established before (18), PBMCs (*ex-vivo*) or cultured cells (1-2x10^6^) were incubated with phorbol 12-myristate 13-acetate (PMA, 20 ng/ml; Sigma-Aldrich) and ionomycin (1 µg/ml; Sigma-Aldrich), or complete media (unrestimulated controls), in the presence of Brefeldin A (5 µg/ml; Sigma-Aldrich) at 37°C, 5% CO_2_, for 4-5 hours. Thereafter, cells were incubated with anti-FcR mAb (15 minutes; RT), and surface-stained (20 minutes; 4°C) with fluorescently-labelled mAbs (from BioLegend, unless stated otherwise): anti-CD3 and anti-CD8 (same as above); PB-labelled anti-CD4; and FITC-labeled anti-CD19. Samples were washed, fixed (as above) and permeabilised with 1X BD PermB2 buffer (10 minutes, RT, BD), as by manufacturer instructions. After another wash, samples were intracellularly stained with PE-Cy7-labeled anti-IFN-γ (B27), AF700-labeled anti-IL-2 (MQ1-17H12) and BV605-labeled anti-TNF-α (MAb11) (30 minutes; RT; in the dark). Intracellular cytokine staining (ICS) samples were finally washed prior to acquisition.

### 2.6 Transcription factors staining of stimulated T cells in HDs and CLL patients

Cultured cells were harvested and washed with FACS buffer (as above) before surface-staining (20 minutes; 4°C) with the following mAbs (from BioLegend, unless stated otherwise): PB-labelled anti-CD4; PE/Cy7-labeled anti-CD8 (HIT8a; BD); APC-H7-labeled anti-CD25 (M-A251; BD); Qdot-605-labeled anti-CD3 (SK7; Invitrogen); and Live/Dead™ Fixable Aqua fluorescent stain (Invitrogen). After a wash with FACS buffer, cell pellets were fixed by resuspension in 1x Foxp3 buffer A (BioLegend) for 10 minutes at RT, in the dark. Fixed samples were washed (FACS buffer; 500 g; 10 minutes, 4°C), supernatants decanted and cells permeabilised in Foxp3 Buffer C (1:50 dilution of Foxp3 Buffer B into 1x Foxp3 Buffer A) (30 minutes; RT; in the dark). After another wash, pellets were resuspended and stained intracellularly with AF647-labeled anti-Foxp3 (150D) (30 minutes; RT; in the dark). Unbound excess mAb was washed before acquisition.

### 2.7 Flow cytometry data acquisition and statistical analysis

Samples were acquired on an LSR-II Fortessa (BD Biosciences) using Diva Software (Version 8), standardised using CS&T beads and rainbow profile SPHERO™ Calibration Particles to reproduce optimal PMTs voltages for each panel. Compensated data was exported for analysis in FlowJO™ (BD) and gated as shown (**Supplementary Figures 1**-**4**). Statistical analyses with GraphPad Prism (Version 9) included Shapiro-Wilk tests to test data normality, prior to unpaired T (parametric data) or Mann-Whitney U tests (non-parametric data), used to test significance between 2 groups. When >2 groups were compared ANOVA or Kruskal-Wallis tests (with Dunnett or Dunn’s *post hoc* tests) were used to determine statistical significance, respectively for parametric or non-parametric data. Levels of statistical significance are: *p ≤ 0.05; **p ≤ 0.005; ***p ≤ 0.0005.

## 3 Results

### 3.1 Reduced naïve CD8 T cell frequency and increased exhaustion phenotype of T cells in ibrutinib-treated CLL patients

Whilst ibrutinib directly reduces CLL cells due to cytotoxicity, it inhibits T cell activation (32, 33), thereby affecting adaptive responses. Hence, we asked whether CLL patients (n=23) showed different distribution of activated T cells, *ex vivo*, dependent on ibrutinib treatment. Based upon whether patients had received ibrutinib (n=10) or were untreated (n=13), we generated two CLL patient sub-cohorts with similar distributions of age, disease stage, and gender (**Table 1**).

**Figure 1:**
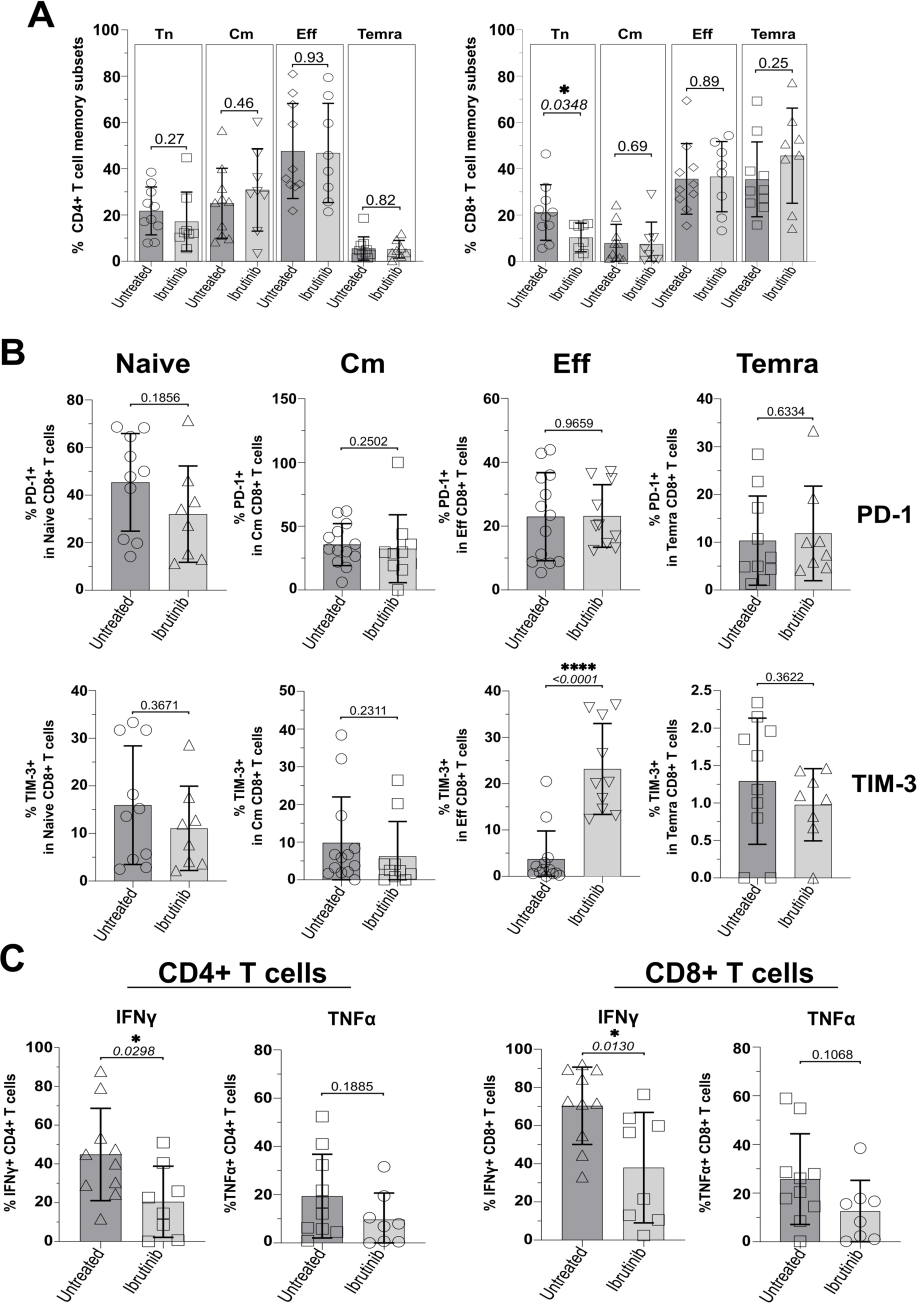
Ibrutinib-treated CLL patients significantly decrease naïve CD8+ T cells and show exhaustion-phenotype effector CD8+ T cells. Fresh PBMCs were immunophenotyped *ex vivo*, comparing ibrutinib-treated (n=8; light grey) and untreated (n=10; dark grey) CLL patients. Data normality was assessed with the Shapiro-Wilk test before evaluating significance with unpaired T or the Mann-Whitney U tests, respectively for parametric or non-parametric data. **(A)** Scatter plots with bars show percentages of naïve (Tn: CCR7+CD45RA+) and memory subsets identified by using CCR7 and CD45RA (Cm; CCR7+CD45RA-, Eff; CCR7-CD45RA-, and Temra; CCR7-CD45RA+), after gating on viable CD3+ CD4+ (left) and CD3+ CD8+ (right) T cells. **(B)** Expression of the exhaustion markers/checkpoint receptors (PD-1 and TIM-3) was investigated in each of the above subsets. Scatter plots with bars show the percentages of PD-1+ (top row) and TIM-3+ (bottom row) within Naïve, Cm, Eff and Temra CD8+ T cells. **(C)** PMBCs were stimulated with PMA/Ionomycin in the presence of Brefeldin A (4h) and surface-stained, fixed and permeabilized, before intracellular cytokine staining with IFNγ and TNFα. Scatter plots with bars show the percentages of IFNγ+ and TNFα+ in CD3+ CD4+ (left) and CD3+ CD8+ (right) T cells. *p ≤ 0.05 and ****p ≤ 0.00005.

Based on previous classification of T cells using surface markers, CD45RA and CCR7 (62), we analysed the phenotype of T naïve-like (Tn: CD45RA+CCR7+) and memory T cells, including the least differentiated, central memory (Cm: CD45RA-CCR7+) and the more differentiated effector/effector memory (Eff/Em: CD45RA-CCR7-) and effector memory reacquiring the expression of CD45RA (TEMRA: CD45RA+CCR7-); refer to **Supplementary Figure 1** for gating strategy. CD4+ T cell subsets were distributed similarly in the two CLL sub-cohorts with no significant changes detected, while trends appeared to point to a non-significant reduction of Tn and a mild accumulation of Cm in ibrutinib-treated CLL patients (**Figure 1A**). In contrast, ibrutinib-treated individuals showed significantly lower frequency of CD8+ Tn cells (p=0.0348, **Figure 1A**).

Given the previous reports of pseudo-exhaustion in CLL (17, 34, 35, 37, 39–41), we analysed expression of checkpoint receptors, TIM-3 and PD-1 (using gates set and validated as by **Supplementary Figures 1** and **2**) and functional cytokine responses in T cell subsets (as gated by **Supplementary Figure 3**). Ibrutinib-treated patients had significantly higher proportions of exhaustion-phenotype TIM-3+, but not PD-1+ Eff/Em CD8+ T cells (p<0.0001, **Figure 1B**). For CD4+ T cells, no differences in the expression of TIM-3 and PD-1 were detected, *ex vivo* (**Supplementary Figures 5A, B**). Interestingly, we noted relatively high expression of PD-1 in CD8+ (**Figure 1B**) and CD4+ (**Supplementary Figure 5A**) T cells with a naïve-like phenotype, consistent with previous reports in CLL patients (63). Relative to HD controls, especially untreated CLL patients showed significantly higher proportions of PD1+ CD4+ (p=0.0009) and CD8+ (p=0.0004) Tn cells (**Supplementary Figures 6A, B**). Despite a reduction in ibrutinib-treated patients, these were not significantly lowered in comparison to untreated CLL patients (**Supplementary Figures 6A, B**).

We wondered whether the significant change in expression of checkpoint receptor, TIM-3 would associate with decreased IFNγ and TNFα secretion in ICS experiments, after a short re-stimulation with PMA/Ionomycin *ex vivo* (refer to Materials and Methods). In contrast to the above reports, ibrutinib-treated patients showed significantly lower IFNγ+ CD4+ and IFNγ+ CD8+ T cell responses than untreated CLL patients (p=0.0298 and 0.013, **Figure 1C**). A similar, non-significant decrease of TNFα was apparent (**Figure 1C**).

Thus, ibrutinib treatment is associated with lower frequency of CD8+ Tn cells, reduced effector cytokine responses and higher expression of exhaustion-associated, TIM-3 in Eff/Em CD8+ T cells.

### 3.2 Preferential exhaustion-phenotype, Cm responses in cultures derived from ibrutinib-treated CLL patients, after exposure to staphylococcal SAgs

Impairment of T cell responses upon ibrutinib treatment may explain the higher risk of severe infection in CLL patients. We then investigated whether T cells from CLL patients mount effective responses after chronic stimulation with TCR stimulants and antigens (Ags) derived from endemic SA (64), commonly detected in CLL patients (19). CLL-patient-derived PBMCs were re-stimulated *in vitro* for 5 days with the polyclonal-activators anti-CD3 and anti-CD28 crosslinking mAbs (αCD3/28), in parallel to staphylococcal SAgs, SEB and TSST-1.

**Figure 2:**
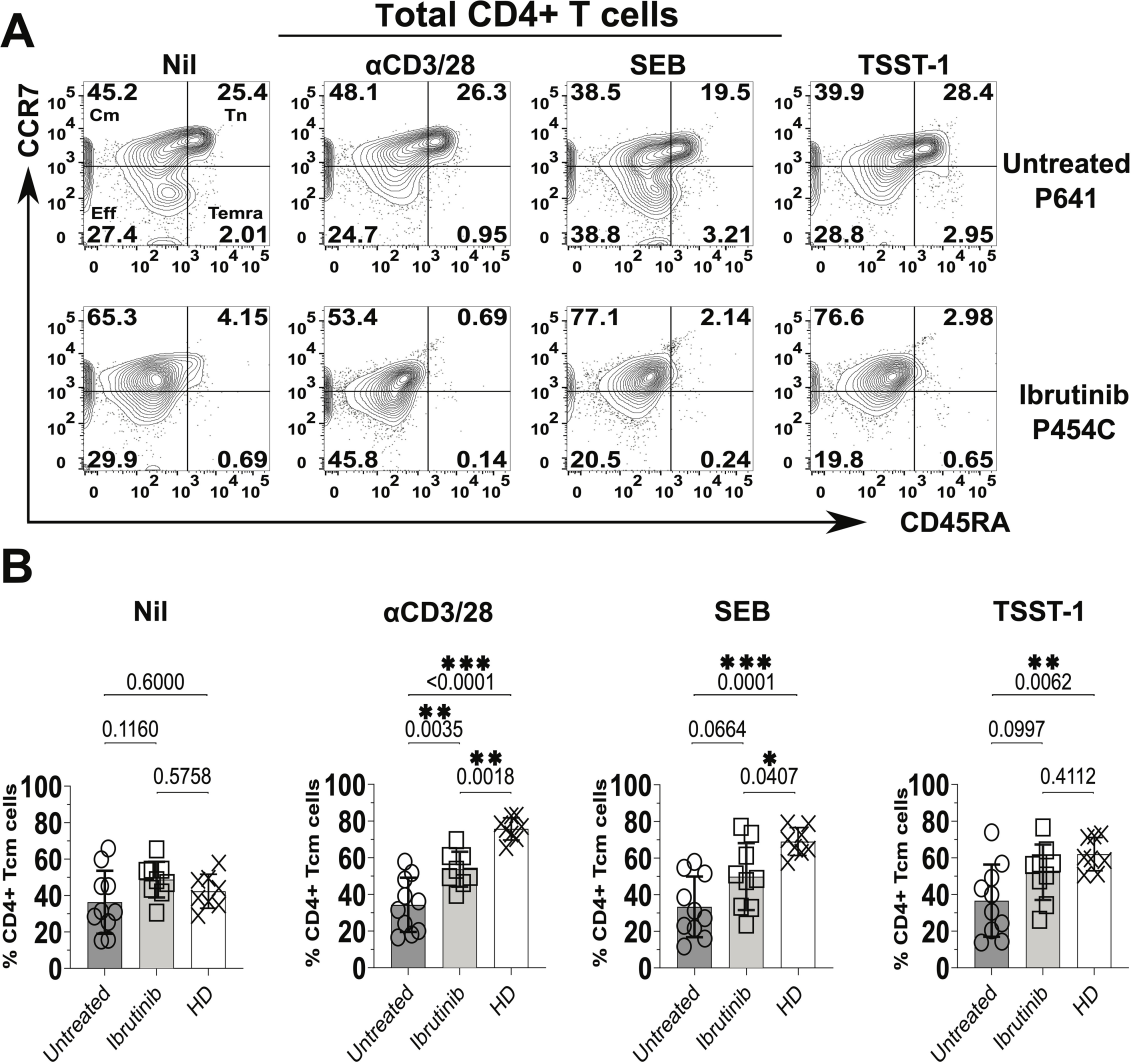
Significant increase of central memory (Cm) CD4+ T cells in cultures derived from ibrutinib-treated CLL patients. Fresh PBMCs from ibrutinib-treated and untreated CLL patients were stimulated *in vitro* for 5 days with stimulatory mAbs or bacterial superantigens, SEB and TSST-1 (refer to Materials and Methods for details) compared to unstimulated control cultures (Nil). Control cultures with the same conditions were established in parallel from HDs. Thereafter, cells were harvested, and surface stained, with anti-CD3, anti-CD4, anti-CD45RA, and anti-CCR7 mAbs. **(A)** Flow cytometric contour plots of example gating of untreated (P641, top) and ibrutinib-treated (P454C, bottom) CLL patients show expression of CD45RA (x axis) and CCR7 (y axis) in gated viable CD4+ T cells to identify subsets as in **Figure 1**. **(B)** Scatter plots with bars show the percentages of Cm+ (CCR7+CD45RA-) CD4+ T cells in ibrutinib-treated (n=9, light grey) and untreated (n=10, dark grey) CLL groups, compared to HDs (n=8, white). Statistical significance was determined after evaluating the data normality with Shapiro-Wilk test using the unpaired T test or the Mann-Whitney U test for parametric or non-parametric data, respectively. *p≤0.05; **p≤0.005; ***p≤0.0005.

In terms of CD45RA and CCR7 expression, we detected heterogenous responses in cultures derived from CLL patients (**Figure 2**). For example, predominant survival/expansion of Cm CD4+ T cells (**Figure 2A**) was notable in cultures derived from the ibrutinib-treated patient P-454C more than in those from the untreated P-641 patient. However, generally, cultures derived from both CLL patient groups showed lower proportions of Cm CD4+ T cells relative to those from HDs, most significantly after αCD3/28 stimulation (p<0.0001 and p=0.0018, respectively for untreated and ibrutinib-treated CLL, **Figure 2B**). This unveiled suboptimal trends towards accumulation of Cm after αCD3/28 stimulation in CLL- (p=0.3255), but not HD- (p=0.0002) derived cultures (compare **Supplementary Figures 7A** and **7B**, respectively). Yet, proportions of Tn cells decreased in αCD3/28 compared to Nil cultures for both HD (p=0.0002) and CLL (p=0.0084) groups (**Supplementary Figure 7**). Similar results were obtained for Cm CD8+ T cells that accumulated in HD- more than CLL patient-derived cultures with αCD3/28 (**Supplementary Figure 8A**). Notably, the underrepresented Eff/Em and TEMRA CD4+ and CD8+ T cell subsets were either marginally or not enriched in the same cultures, including in the case of HDs (**Supplementary Figures 9A, B**, **8B, C**).

It is worth noting that TCR stimulations (including αCD3/28 and SA SAgs, SEB and TSST-1) significantly associated with enlarged proportions of Cm CD4+ T cells in HD-, but not CLL-derived cultures (**Supplementary Figure 10**). The latter showed a trend towards predominant Cm phenotypes under any of the tested culture conditions (including the unstimulated Nil), with some non-significant trends apparent when cultures were established from ibrutinib-treated CLL patients (**Supplementary Figure 10**, bottom panels). When directly compared to untreated CLL counterparts, ibrutinib-treated patients showed a moderate increase in Cm CD4+ T cell proportions, statistically significant after αCD3/28 stimulation (p=0.0035, ANOVA, **Figure 2B**). Except for this, overall conventional markers that distinguish naïve-like from activated T cells (CD45RA and CCR7) did not yield to significant detection of responses in CLL patient-derived cultures after exposure to TCR stimulants, most relevantly to SA SAgs.

We reckoned that SA SAgs may instead promote the accumulation of responses with an exhaustion phenotype, which would be more easily detectable by combining PD-1/TIM-3 with the above markers. In cultures derived from ibrutinib-treated rather than untreated patients, the expression of the exhaustion marker PD-1 significantly increased in memory (CD45RA-, *i.e.* Cm + Eff/Em) CD4+ T cells after αCD3/28 stimulation (p=0.0008, **Supplementary Figure 11**). Higher proportions of Cm and Eff/Em CD4+ T cells upregulated PD-1 in αCD3/28 cultures from ibrutinib-treated patients compared to untreated counterparts (~60% of Cm cells, p=0.0028 and ~80% of Eff/Em cells, p=0.0007, **Supplementary Figure 11C**). Independently of ibrutinib treatment, exposure to SEB and TSST-1 increased the proportions of PD-1+ CD45RA- T cells by ~4-fold, within the total CD4 T cells and memory cell populations, relative to Nil (no SAg) control cultures (**Supplementary Figure 11**). Statistically pooled and concatenated data analyses revealed that CD4+ T cells from CLL patient-derived cultures upregulated levels of PD-1 (as mean fluorescence intensity, MFI) much more after exposure to SA SAgs than αCD3/28 mAbs or in the Nil control cultures (**Supplementary Figures 12A, C**), not dependent on ibrutinib treatment (**Supplementary Figure 13A**).

**Figure 3:**
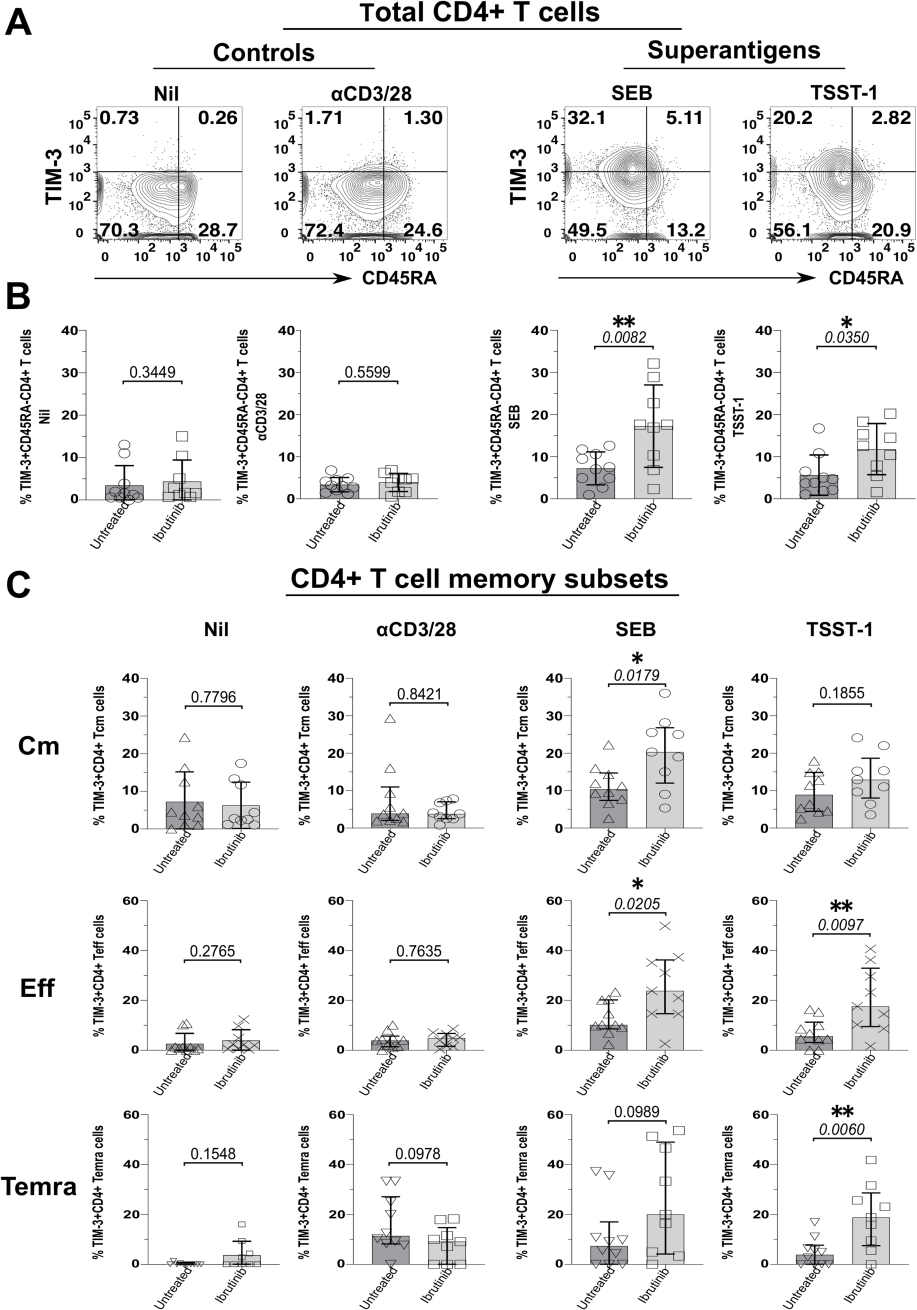
Staphylococcal SAgs drive upregulation of the TIM-3 exhaustion marker in memory CD4+ T cells, preferentially in cultures derived from ibrutinib-treated CLL patients. PBMCs of ibrutinib-treated and untreated CLL patients were stimulated *in vitro*, as described in **Figure 2**. At day 5 of culture, cells were harvested and surface stained as indicated in the Materials and Methods section. **(A)** Flow cytometric contour plots of example patient P590 (ibrutinib-treated CLL) show surface expression of CD45RA (x-axis) and TIM-3 (y-axis) within viable CD4+ T cells after 5d-stimulation with superantigens, SEB, and TSST-1 (left) compared to control conditions (right panels): αCD3/28 and unstimulated (Nil) cultures. **(B)** Scatter plots with bars show the percentage of TIM-3+ cells within total memory CD45RA- CD4+ T cells detected in the above cultures derived from either ibrutinib-treated (n=9) or untreated (n=10) CLL patients. **(C)** Scatter plots with bars demonstrate the percentage of TIM-3+ cells within Cm (top row), Eff (middle row) and Temra (bottom row) CD4+ T cell subsets in the same cultures comparing ibrutinib-treated (n=9) and untreated (n=10) CLL patients. Statistical significance was determined after evaluating the data normality with Shapiro-Wilk test using the unpaired T test or the Mann-Whitney test for parametric or non-parametric data respectively. *p ≤ 0.05 and **p ≤ 0.005.

Moreover, TIM-3+ CD45RA- CD4+ T cells significantly increased in cultures from ibrutinib-treated more than untreated patients, upon exposure to SEB (p=0.0082) and TSST-1 (p=0.035), but not αCD3/28 (**Figures 3A, B**). Cm CD4+ T cells expressing TIM-3 rose significantly in SEB (p=0.0179, **Figure 3C**) and non-significantly in TSST-1 cultures derived from ibrutinib-treated over the untreated patients (**Figure 3C**). Significantly higher proportions of TIM-3+ Eff/Em CD4+ T cells were detected after exposure to SEB (p=0.0205) and TSST-1 (p=0.0097) (**Figure 3C**) in cultures from ibrutinib-treated patients. Similarly, these patients showed significantly higher TIM-3+ TEMRA CD4+ T cell responses, after TSST-1 stimulation (p=0.006, **Figure 3C**), with trends noted even after SEB stimulation (p=0.0989), when compared to the untreated CLL group. MFI analysis of TIM-3 expression, either in statistical pools (**Supplementary Figure 12B**) or concatenated data (**Supplementary Figure 12D**) did not yield as many statistically significant differences as detected in percentage subset analyses (**Figure 3**). However, the concatenated data analysis confirmed the trends of more prominent TIM-3 expression in ibrutinib-treated rather than untreated CLL patient-derived cultures, particularly after SA SAg exposure (**Supplementary Figure 13B**).

Opposite to the case of CD4+ T cells, combining PD-1 (**Supplementary Figure 14**) and TIM-3 (**Supplementary Figure 15**) with CD45RA and CCR7 still did not help achieve significant responses of CD8+ T cells to SA SAgs, in cultures derived from the same CLL patients.

Thus, in chronic Ag-stimulation settings, T cells from CLL patients poorly respond to TCR stimulants in terms of CD45RA/CCR7 subset distribution. While ibrutinib treatment associates with moderate yet significant accumulation of Cm T cells, these and other memory (Eff/Em and TEMRA) T cells readily upregulate checkpoint receptors (TIM-3 and PD-1) after SA SAg-exposure. Hence, pseudo-exhaustion markers most significantly help in the identification of responses to TCR stimulants, in the context of CLL, at least *in vitro*.

### 3.3 Suboptimal Treg expansion upon SAg-exposure in CLL patients

**Figure 4:**
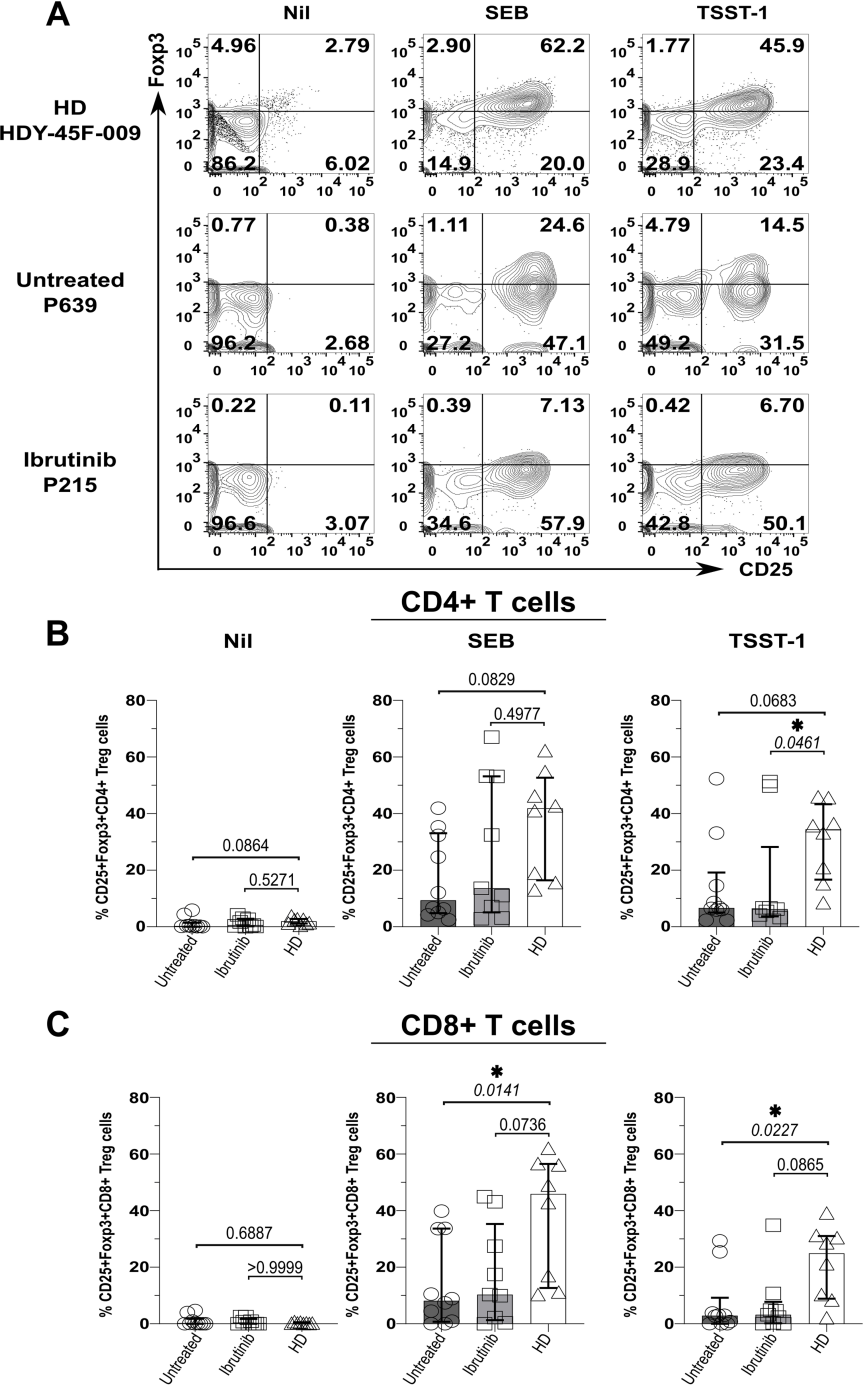
Suboptimal CD4+ (and CD8+) Treg expansion upon SAg/TSST-1-exposure in CLL patients. PBMCs of HDs and ibrutinib-treated or untreated CLL patients were stimulated with SEB and TSST-1 compared to unstimulated controls (Nil) for five days. Cells were then harvested, surface-stained with anti-CD3, anti-CD4, and anti-CD8 mAbs and Aqua/Live stain, then fixed, permeabilised and intracellularly stained with Foxp3 mAb (as described in the Materials and Methods), prior to flow cytometry acquisition. **(A)** Contour plots show expression of the Treg-signature transcription factor, Foxp3 (y-axis) against CD25+ (x-axis) in viable CD3+ CD4+ T cells from example HD (HDY-45F-009; top row), untreated (P639; middle row) and ibrutinib-treated (P215; bottom row) CLL patients after stimulation with SEB (middle column) and TSST-1 (right column), compared to Nil controls (left column). **(B, C)** Scatter plots with bars show the percentages of CD25+ Foxp3+ CD4+ **(B)** and CD8+ **(C)** Tregs between ibrutinib-treated (n=9; light grey), untreated (n=10; dark grey) CLL patients and HDs (n=8; white) detected in the above cultures. Statistical significance was determined after assessing the data normality with Shapiro-Wilk test using the one-way ANOVA test or the Kruskal-Wallis test with Dunnett or Dunn’s multiple comparison correction tests for parametric and nonparametric data respectively. *p ≤ 0.05.

As bacterial SAgs can induce the generation of Tregs (65, 66), which express high levels of the above checkpoint receptors (67), we wondered if levels of Foxp3 (*i.e.*, the Treg-signature transcription factor) would rise in T cells from SAg-stimulated cultures. Compatible with previous evidence (65, 66), cultures from HDs (HDY-45F-009 shown as an example in **Figure 4A**) markedly accumulated CD25+ Foxp3+ CD4+ T cells upon SAg-exposure (**Figures 4A, B**). Surprisingly, unlike HDY-45F-009, untreated (P-639) and ibrutinib-treated (P-215) CLL patients failed to mount similarly large Treg responses (**Figure 4A**). Percentages of CD25+ Foxp3+ CD4+ T cells remained much higher in the HD group relative to ibrutinib-treated and untreated CLL sub-cohorts, significantly after TSST-1 exposure (p=0.0461, ibrutinib-treated CLL) (**Figure 4B**). Similarly, CD25+ Foxp3+ CD8+ T cells expanded in SAg-cultures derived from HDs, but not in those derived from untreated CLL patients after TSST-1 (p=0.0227) and SEB exposure (p=0.0141) (**Figure 4C**). The ibrutinib-treated and untreated CLL sub-cohorts did not differ for the percentages of CD25+ Foxp3+ CD4+ (**Figure 4B**) and CD8+ (**Figure 4C**) T cells.

Hence, in CLL, exposure to SA SAgs fails to induce Treg responses as large as those observed in HDs. In this respect, ibrutinib treatment does not fully recover the ability of T cells from CLL patients to mount SAg-driven immunoregulatory responses.

### 3.4 Enhanced polyclonal inflammatory cytokine responses in T cells from CLL patients upon exposure to SAg

**Figure 5:**
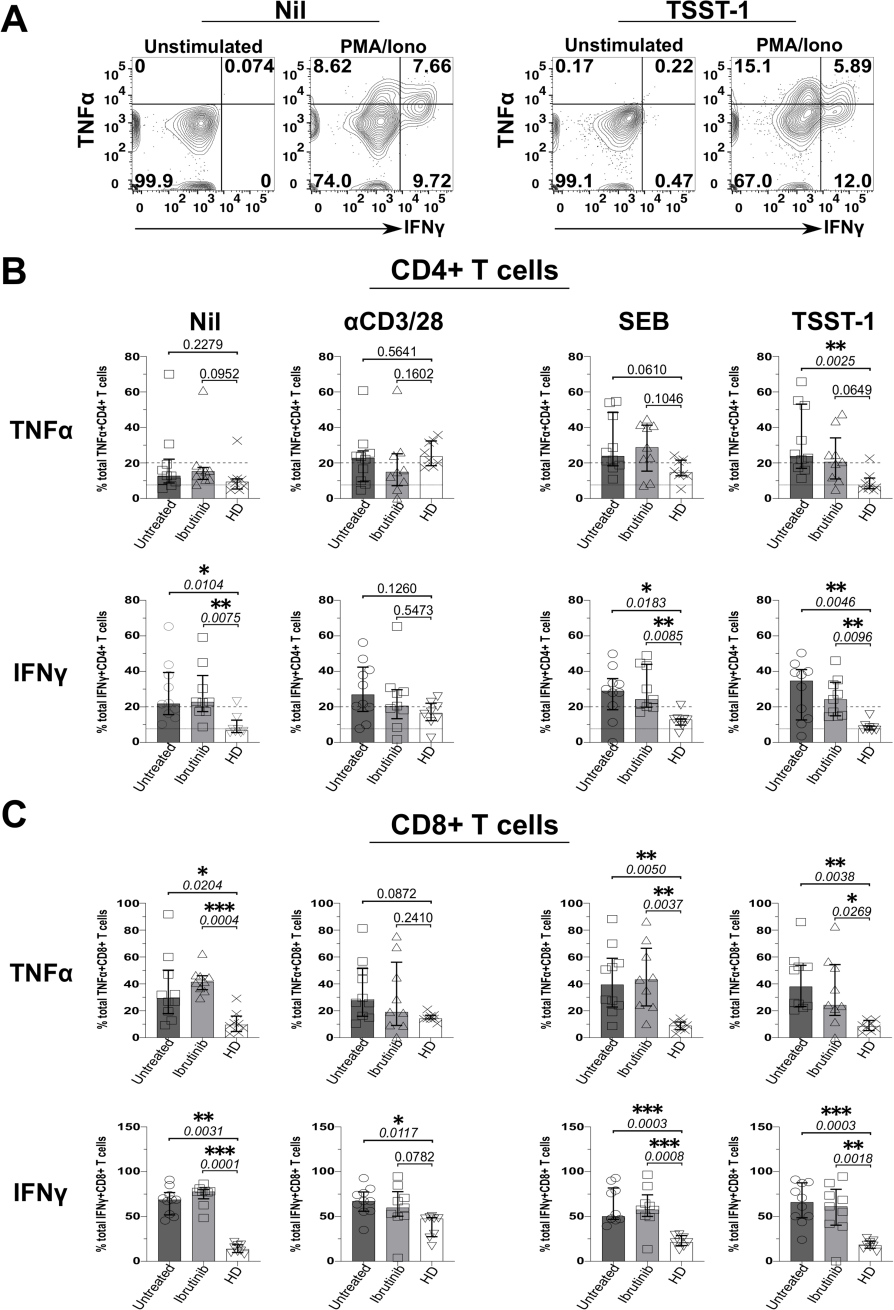
Staphylococcal superantigen (SAg) toxins, SEB and particularly TSST-1 enhance inflammatory TNFα and IFNγ responses in T cells from CLL patients. PBMCs derived from CLL patients and HDs were stimulated *in vitro*, with αCD3 and αCD28 mAbs or bacterial superantigens, SEB and TSST-1 (refer to Materials and Methods for details), compared to control media cultures (Nil). After 5 days, cultured cells were briefly re-stimulated with PMA/Ionomycin (PMA/Iono) in the presence of Brefeldin A (4 hrs), surface stained with fluorescently-labelled anti-CD3, anti-CD4 and anti-CD8 mAbs; fixed; permeabilised; and intracellularly stained with IFNγ and TNFα mAbs in ICS. **(A)** Flow cytometry plots show the percentage of IFNγ+/TNFα+ CD4+ T cells after exposure to TSST-1 (right panels) compared to no-SAg control cultures (Nil, left panels), after re-stimulation with PMA/Iono or in unstimulated ICS controls (an example of gating in ibrutinib-treated patient, P251 is provided). **(B, C)** Scatter plots with bars show the statistical analysis of percentages of total TNFα+ (top rows) and IFNγ+ (bottom rows) in CD4+ **(B)** and CD8+ **(C)** T cells as determined by ICS, comparing cultures derived from ibrutinib-treated (n=9; light grey bars) and untreated (n=10; dark grey bars) CLL patients, relative to HDs (n=8; white bars). After assessing data normality with Shapiro-Wilk tests, statistical significance was determined by using the one-way ANOVA or the Kruskal-Wallis tests, respectively with Dunnett or Dunn’s multiple comparison correction tests for parametric and nonparametric data. *p ≤ 0.05; **p ≤ 0.005; ***p ≤ 0.0005.

We next investigated whether defective Treg responses to SAgs concur with heightened inflammation in CLL. Thus, we measured secretion of IFNγ and TNFα from T cells in the above cultures, by ICS. Data from an example HD showed that, while unstimulated ICS controls typically yielded little cytokine secretion, more CD4+ T cells secreted IFNγ and TNFα in TSST-1 than Nil (no SAg) control cultures (**Figure 5A**). CD4+ and CD8+ T cells produced more IFNγ and TNFα in cultures derived from CLL patients rather than HDs, independent of ibrutinib-treatment (**Figures 5B, C**). Though some significant trends appeared in Nil cultures, both TNFα (p=0.0025 and 0.0649) and IFNγ (p=0.0046 and 0.0096) increased significantly after TSST-1 exposure in CLL patient-derived cultures compared to HD-derived counterparts (**Figure 5B**). Weaker, but yet similar trends were detected in SEB cultures, particularly significant for IFNγ+ CD4+ T cells (p=0.0183 and 0.0085) (**Figure 5B**). Notably, SEB/TSST-1 promoted higher IFNγ release than the levels detected with pan-T-cell, αCD3/28 stimulation in both CLL sub-cohorts (contrary to HDs), indicating a potential bias towards responding to SA SAgs, in CLL patients (**Figure 5B**).

Altogether these data suggest that T cells from CLL patients readily respond to SA SAgs, heightening inflammatory responses in CLL.

### 3.5 Staphylococcal SAgs increase activation and inflammatory potential of CLL tumour cells

We next sought to investigate whether CLL tumour cells have ability to participate in inflammation driven by SAgs, and if ibrutinib could prevent this.

**Figure 6:**
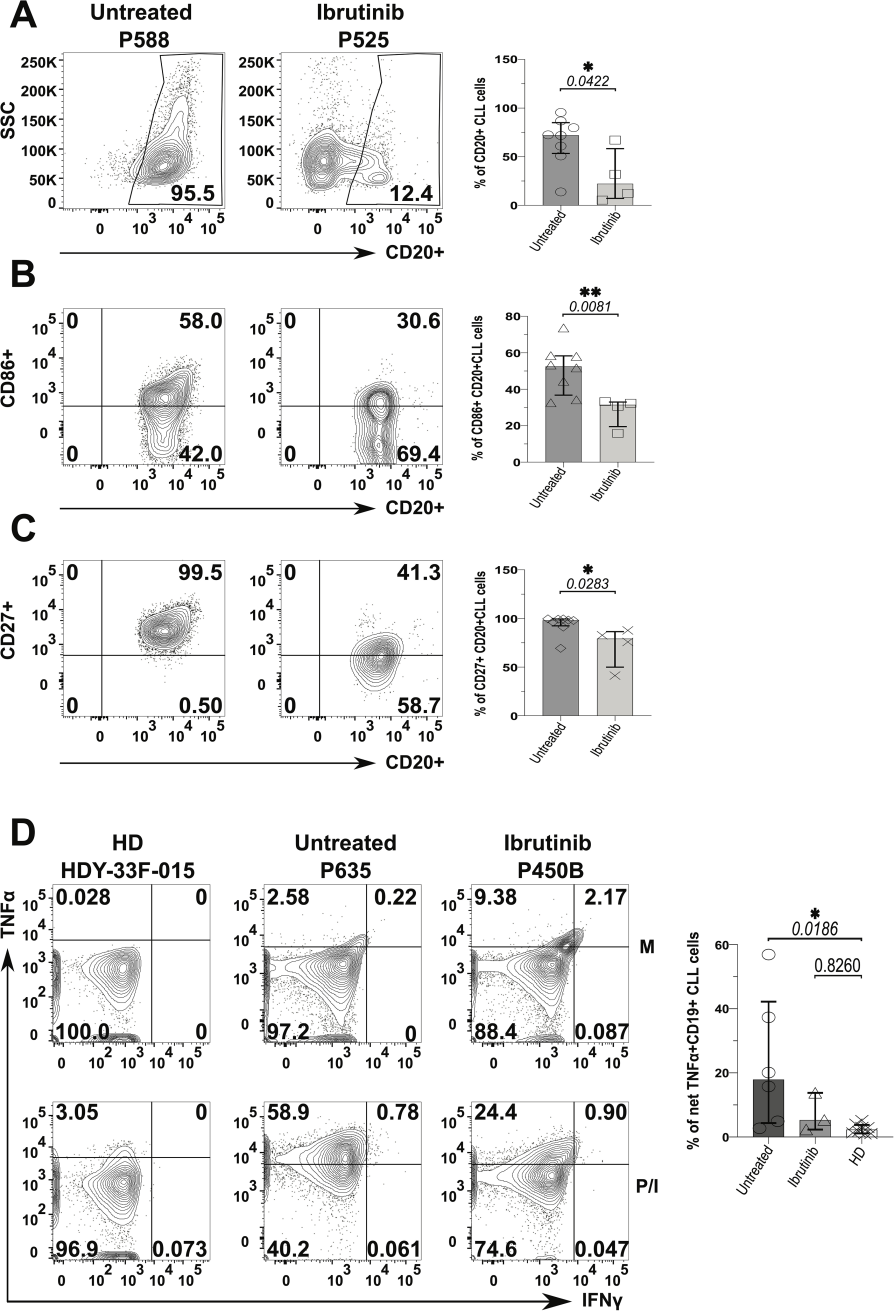
Decreased pro-inflammatory phenotype of CLL tumour cells derived from ibrutinib-treated patients. Fresh PBMCs were immunophenotyped *ex vivo* after surface staining with anti-CD20, anti-HLA-DR (not shown), anti-CD86 and anti-CD27 mAbs (as described in the Materials and Methods) to compare ibrutinib-treated (n=4; light grey) to untreated (n=8; dark grey) CLL patients. Data normality was assessed with the Shapiro-Wilk test before evaluating significance with unpaired t- or the Mann-Whitney U tests, respectively for parametric or non-parametric data. **(A)** Viable CD20+ CLL cells (*i.e.* tumour cells) were identified by using side-scatter (SSC) against CD20 expression. Scatter plots with bars show the median ± IQR frequency of CD20+ CLL cells in the above patient groups using example patients, P588 and P525 (respectively untreated and ibrutinib-treated CLL). **(B, C)** Contour plots with relative bar graphs show expression of surface markers: CD86 **(B)** and CD27 **(C)** (median ± IQR) in gated CD20+ CLL cells respectively from the same example patients shown in A and the pooled patient groups. **(D)** PMBCs were stimulated with PMA/Ionomycin (P/I), relative to unstimulated media controls (M) in the presence of Brefeldin A (4 hrs). Thereafter, cells were surface stained with anti-CD19 and anti-CD3 mAbs, fixed and permeabilized, before ICS with anti-IFNγ and anti-TNFα mAbs. Contour plots show gating examples of HDs (HDY-33F-015) and CLL patients: P635 and P450B (respectively untreated and ibrutinib-treated CLL), reporting the percentage expression of TNFα (y-axis) and IFNγ (x-axis). the bar graph shows the percentage of net TNFα+/IFNγ+ in CD19+ (CD3-) CLL cells detected in HDs (n=8) compared to untreated (n=6) and ibrutinib-treated (n=3) CLL patients, *ex-vivo*. For each individual, net cytokine responses were the subtraction of background cytokine levels in M controls (top) from percentage cytokine+ cells of the P/I stimulated samples (bottom), within a given gate. The right bar graph shows the relative statistical analysis (ANOVA). *p ≤ 0.05 and **p ≤ 0.005.

Frequency of CLL cells was ~5-fold lower in the ibrutinib-treated sub-cohort compared to the untreated counterpart (p=0.0422, **Figure 6A**), reaching levels of B cells found in HDs (12.4%, 95.5%, and 10%, respectively, as shown in **Supplementary Figure 16**). Yet, while ibrutinib-treated individuals showed significant lower tumour burden, as expected (47, 48, 51, 68), residual activated CLL cells persisted. Previously described as proliferative (69), CD86+ CLL cells significantly declined in frequency, in ibrutinib-treated compared to untreated CLL patients (p=0.0081; *e.g.*, 30.6% and 58%, respectively; **Figure 6B**), still lower than in HDs (p=0.0024, **Supplementary Figure 16**). Equally, memory-like (70) CD27+ CLL cells showed a significant moderate decrease in ibrutinib-treated compared to untreated CLL patients (p=0.0283; *e.g.*, 41.3% and 99.5%, respectively in **Figure 6C**), higher than in HDs (p=0.0002, **Supplementary Figure 16**, bottom panels). When measuring inflammatory cytokine secretion, generally low IFNγ responses appeared in B/CLL cells. However, after cytokine-background subtraction, up to 50% and ~14% of CLL cells from untreated and ibrutinib-treated patients produced TNFα, much higher than levels measured in HD B cells (<5%), particularly significant for untreated CLL patients (p=0.0186, **Figure 6D**). Thus, ibrutinib-treated patients preserved an albeit reduced, yet sizeable fraction of CLL cells producing TNFα, with levels intermediate between those of HDs and untreated CLL patients.

**Figure 7:**
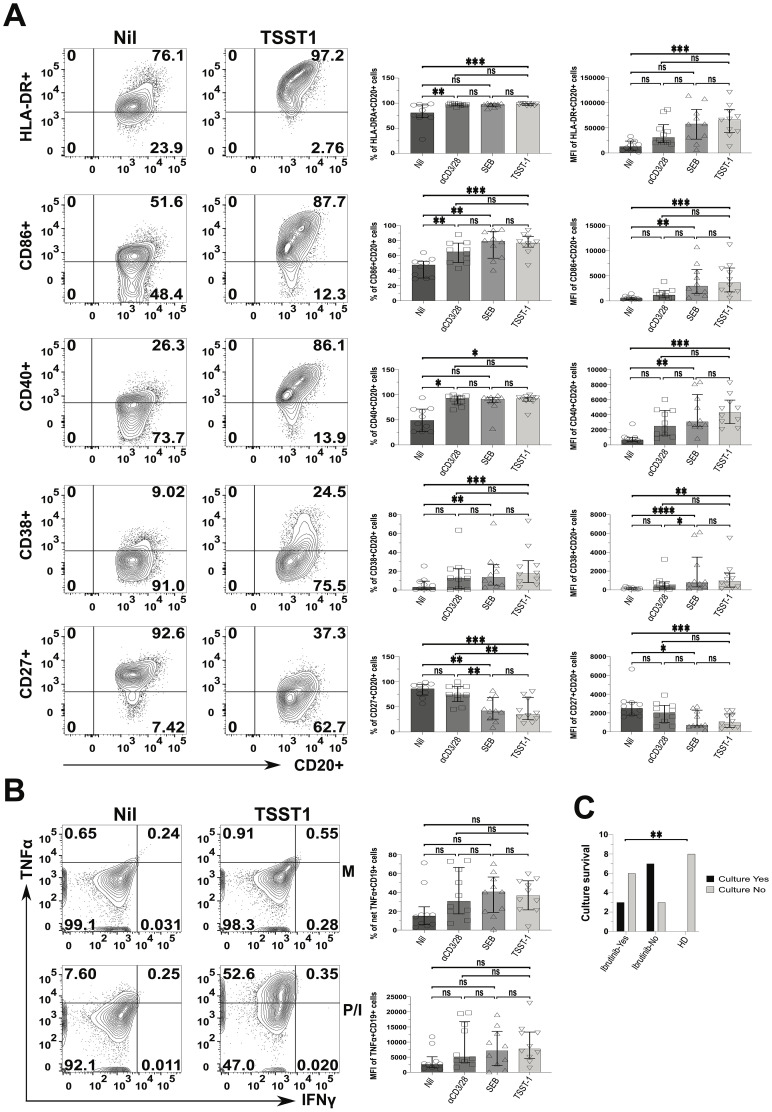
Staphylococcal SAg/TSST-1 significantly promotes the pro-inflammatory phenotype of CLL tumour cells, allowing them to survive/grow *in vitro*. PBMCs freshly derived from ibrutinib-treated and untreated CLL patients were stimulated with αCD3 + αCD28 mAbs or SEB and TSST-1, in comparison to control media unstimulated cultures (Nil). After 5 days, cultured cells were surface-stained as described in the Materials and Methods and viable CD20+ CLL cells were then identified as in **Figure 6**. **(A)** Left contour plots show the expression of activating surface markers, namely in ibrutinib-treated CLL patient P638, as an example: HLA-DR (top row), CD86 (second row), CD40 (third row), CD38 (fourth row) and CD27 (bottom row) in gated CD20+ CLL cells, in cultures exposed to the TSST-1 SAg (second column), compared to control media cultures (Nil, first column). Right scatter plots with bars show statistical analyses of percentages (third column) and mean florescence intensities (MFIs; fourth column) for each of the markers shown, in cultures derived from multiple CLL patients (pooled statistical analyses). **(B)** TSST-1 and control media cultures were re-stimulated with PMA/Ionomycin (P/I), relative to unstimulated media controls (M), and processed in ICS as described in **Figure 6**. Contour plots from the untreated CLL patient (P418) show the percentage of total TNFα+/IFNγ+ in CD19+ (CD3-) CLL cells detected in the above conditions, as a gating example. Right scatter plots with bars show statistical analyses of percentages (top right graph) and MFI (bottom right graph) of TNFα content in cultures derived from different patients. **(C)** The graph shows the numbers of successful (black bars) and unsuccessful (light grey bars) outcomes of SAg/TSST-1-driven cultures (expressed as a binary YES/NO variable; y-axis), dependent on whether these were derived from ibrutinib-treated or untreated CLL patients and HDs (x-axis). X2 test shows that TSST-1-driven cultures derived from CLL patients had significantly more successful rates (7/10 and 3/9, respectively for untreated and ibrutinib-treated patients) than those derived from HDs (0/8; *i.e.* no culture from HDs expanded B cells upon TSST-1 exposure). Unless stated otherwise, statistical significance was assessed after analysing data normality (Shapiro-Wilk test) by using one-way ANOVA or the Kruskal-Wallis tests with Dunnett or Dunn’s multiple comparison correction tests, respectively for parametric or non-parametric data. *p ≤ 0.05; **p ≤ 0.005; and ***p ≤ 0.0005. ns, non-significant.

We wondered if such CLL cell activation would escalate upon SA SAg-exposure. To investigate this, we examined CLL PBMC cultures containing autologous responder T cells. We compared the response of SA SAgs to polyclonal αCD3/28 stimulation, which served as a benchmark for T cell help. The expression of HLA-DR, CD86 and CD40 (percentages and MFI) by CLL cells rose significantly in SEB, TSST-1 and αCD3/28 cultures alike, relative to the Nil (no SAg) controls (*e.g.*; patient P638 in **Figure 7A**). Still, MFI analyses indicated more significantly pronounced activation from SAgs, especially TSST-1, surpassing that of αCD3/28. Further, the CLL activation marker, CD38 significantly increased in CLL cells cultured with TSST-1/SEB, but not αCD3/28, compared to Nil cultures. SAgs significantly reduced CD27+ CLL cells, not mirrored by T cell help provided in αCD3/28 cultures, which remained comparable to Nil controls (**Figure 7A**). The statistical analyses of percentages and MFIs (**Figure 7A**) were further corroborated by concatenated data analyses of CLL cells in the same cultures (**Supplementary Figure 17**). From these, it appeared that HLA-DR, CD86, CD38 and CD40 were powerfully upregulated on CLL cells, especially in SA SAg cultures. Concatenated analysis of CD27 showed downregulation in CLL cells (1508 down to 556) when comparing Nil to αCD3/28 cultures (hence pointing at a potential role of T cell help), further reduced (to 271) in TSST-1 cultures (**Supplementary Figure 17**). Additionally, CLL cells tended to secrete more TNFα in cultures with SAgs and αCD3/28 (~2-fold higher), compared to Nil controls (**Figure 7B**). Significantly different from CLL cells, HD-derived B cells did not activate/survive in staphylococcal SAg cultures (**Figure 7C**, **Supplementary Figure 18**). In this respect, CLL cells from untreated patients were more reliant upon SAg/TSST-1 to survive/activate in culture than their counterparts derived from ibrutinib-treated patients (70% *vs* 33%, p≤ 0.005) (**Figure 7C**). However, in 3 out of 9 ibrutinib-treated CLL patients, we observed that leukemic cells could still activate and proliferate in cultures with SA SAgs (*e.g.*, the ibrutinib-treated patient P638 in **Figure 7A**) similarly to counterparts derived from untreated CLL patients (**Supplementary Figure 18**).

Thus, CLL cells can activate and promote inflammation, enhanced by staphylococcal SAg with such changes not being fully abolished in ibrutinib-treated patients. This indicates that environmental SAg-exposure may perpetuate chronic inflammation in CLL patients, by activating both T and tumour cells. In the context of SA SAg exposure, HD-derived B cells would not benefit from T cell help as much as CLL cells can do.

## 4 Discussion

**Figure 8:**
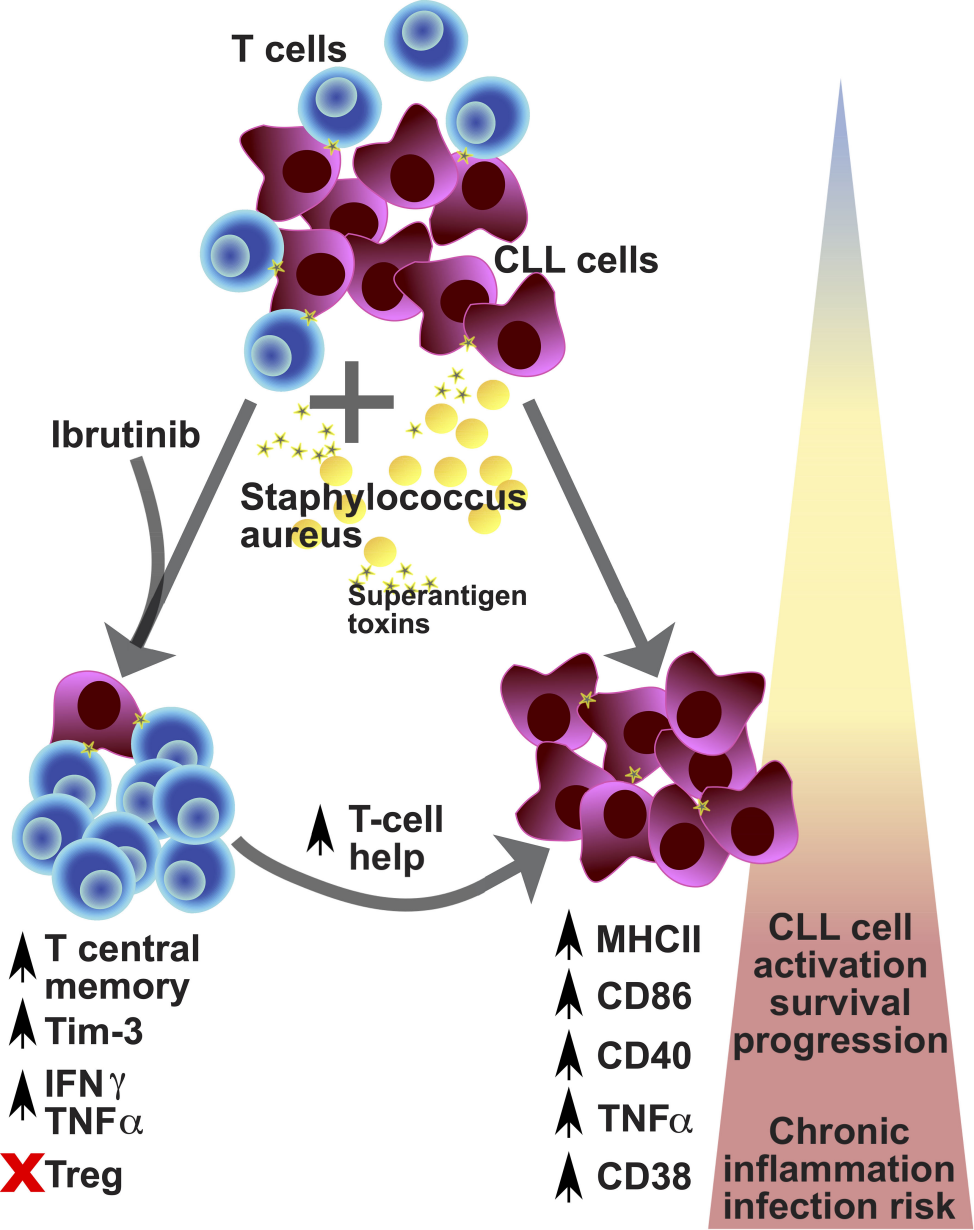
Proposed model for the contribution of environmental staphylococcal SAg to the CLL tumour progression. CLL is a relatively rare condition that arises in aged individuals characterized by the expansion of tumour-transformed B cells (violet cells) and impacting on the T cell repertoire and function (blue cells). CLL patients poorly respond to prophylactic immunisations against pathogens and more frequently experience infections, with sepsis (a critical condition associated with severe inflammatory responses after infection) being the commonest cause of death. Recently, carriage of *Staphylococcus aureus* (yellow cells) has been reported to occur more frequently in CLL patients than healthy subjects. This pathogen can express a variety of toxins, including superantigens (SAg; yellow stars), such as TSST-1 and SEB. After exposure to SAg, we found preferential expansion of central memory T cells which acquire checkpoint receptor/exhaustion marker (TIM-3 and PD-1) expression, associated with high levels of cytokine secretion (TNFα and IFNγ) – known as pseudo-exhausted T cells – in CLL patient-derived cell cultures (bottom left part of the diagram). This bias is particularly significant in patients that receive ibrutinib treatment (although not exclusive to them) and is paralleled by a reduced expansion of regulatory T cells, after SAg exposure. Under these conditions, we found that SAgs powerfully enhance a T cell helper activity in cultures characterized by further tumour cell activation/progression, with the acquisition of markers associated with more aggressive tumour cell behaviour: high expression of MHC-II, CD86, CD40, CD38 and TNFα (bottom right part of the diagram). Hence, exposure to SAg toxins derived from endemic pathogens may significantly impact tumour cell survival and the CLL microenvironment, by promoting a shift in the pro-inflammatory milieu which can contribute to tumour progression, while increasing the risk of severe infection, including sepsis. Based on our results/model, we propose that controlling staphylococcal infection occurrence/carriage may critically help improve untreated and treated CLL patient management and outcomes.

Over the past decade, many studies reported the morbidity of infections in CLL, including during treatment with ibrutinib (26, 47–51) and other BTKis (23, 52–55). Mounting evidence suggests that ibrutinib restores T cell function (34–37, 39–41), but the conundrum is why this does not translate in better protection from infections. Our study unveils that, while ibrutinib treatment promotes long-term memory responses, these may easily veer towards T cell exhaustion under the strain of repeat SA SAg-exposure (**Figure 8**). However, in association with defective Treg induction, these preserve cytokine production, contributing to the inflammatory activation of CLL cells (**Figure 8**).

Chronic inflammation (71), heightened by exposure to endemic pathogens especially under the ibrutinib regimen may explain risk of severe infection outcomes, including sepsis, in CLL patients. SA infections occur in nearly half of CLL patients, more than in the general population (19). Frequently, SA triggers sepsis (13, 72, 73) characterised by pro-inflammatory dysfunction, organ damage and high mortality (74).

Ibrutinib-induced immunosuppression may arise via the inhibition of Btk in other hematopoietic cells (34, 75, 76) beyond B cells (68) and via off-target effects on Itk (32) required for TCR-signalling (32, 33, 77). Upon covalently binding to Tec/Itk, *de novo* treatment with ibrutinib diminished the ability of T cells to respond to TCR stimuli. While we also confirmed off-target Tec/Itk inhibiton in a previous unrelated study (61), other off-targets effects of ibrutinib in T cells cannot be excluded and remain to be investigated in future studies. In contrast to the well-established inhibitory role of ibrutinib in short-term *de novo* administration settings, improvement of T cell responses and even reversal of exhaustion have been associated with ibrutinib long-term treatment, *in vivo* (17, 35–37, 39–41, 78, 79). Further in CLL, ibrutinib may favour Th-1 (32) or Th-17 (35) polarisation over Th-2 responses, helping anti-cancer immunity. However, other conflicting studies reported decreased T cell function under ibrutinib regimen (34, 80). Foremost, we found that long-term ibrutinib-treated patients showed reduced CD8+ Tn cells and higher expression of the exhaustion marker, TIM-3 concurrent with diminished effector cytokine (IFNγ) responses. This supports that “pseudo-exhaustion” (17) [or reversal of exhaustion (38, 40)] may not ensue in ibrutinib-treated patients, universally. Further, how ibrutinib affects the thymic development of CD8+ Tn cells (which critically help fight infections and cancer) is unclear and future research is needed in this respect. Furthermore, we found a significantly increased frequency of PD-1+ CD4+ and CD8+ Tn cells in *ex vivo* analyses of CLL patients, consistent with previous reports (63). The expression of checkpoint receptors on naïve-like T cells might appear somewhat counterintuitive, yet other studies have reported high PD-1 expression in naïve-phenotype (CCR7+ CD45RA+) cells in chronic cancer and infectious conditions. For instance, high PD-1+ naïve-like cells were described in chronically infected HIV (81) and TB (82) patients, while ~5% of naïve-like CCR7+ CD45RA+ T cells were found within PD-1+ T cells in lung cancer patients (83). Previously “naïve-like” Ag-experienced cells were identified within the CCR7+ CD45RA+ gate (84). Hence, based on patient history of exposure to Ag-(including cancer-Ag), CCR7+ CD45RA+ cells may contain cells with higher expression of exhaustion markers, including PD-1, possibly because of exposure to chronic cancer/infection. In these respects, future studies focused on naïve-like T cells of CLL patients would be needed to clarify how these acquire the expression of exhaustion markers during CLL.

While previous CLL studies characterised T cell responses after short polyclonal re-stimulation *ex vivo* (17, 35), we robustly assessed the ability of patient-derived T and CLL cells to respond to chronic Ag signals in cultures that mimic repeat SAg-exposure. Cells derived from CLL patients (but not HDs) reacted to staphylococcal SAgs often to the same extent or more than observed with the pan-T-cell activation elicited with αCD3/28 antibodies. In fact, by using CD45RA and CCR7, we detected relatively weak and heterogenous responses to αCD3/28 TCR-stimulation in CLL patients, compared to HDs (**Figure 2**, **Supplementary Figures 7**-**9**). This is compatible with previous pioneering studies suggesting that, in CLL, T cells suffer from impaired TCR responsiveness and/or biased functionality, potentially because of cancer immunosuppression [Reviewed in (85)]. For instance, CD4 and CD8 T cells of CLL patients are defective in expression of genes involved in TCR signalling in the JNK–p38 MAPK and PI3K pathways, cytoskeleton remodelling and the immune synapse formation (86), associated with defective activation of LAT (87), and decreased proliferation to αCD3/28 (17). Significantly, when exhaustion markers, TIM-3 (**Figure 3**) and to a lower extent PD-1 (**Supplementary Figures 11**-**13**) were combined with CD45RA and CCR7, we detected responses to staphylococcal SAgs in CLL patients, often more sustained than those mounted upon αCD3/28 exposure (**Figure 3**, **Supplementary Figures 12**, **13**). Additionally, this may provide a possible explanation for the TIM-3 upregulation detected *ex vivo* in the ibrutinib-treated sub-cohort. In fact, these may reflect ongoing *in vivo* responses to endemic antigens/stimulants, being more sustained in ibrutinib-treated (read further below for more insights around this) rather than untreated CLL patients. This evidences that CLL patients may contain a higher fraction of (previously activated) SAg-specific cells within the T cell repertoire. These results would suggest that CLL patients experience more repeat infections with SA.

Staphylococcal SAgs trigger oligoclonal T cell expansions by directly binding to specific TCR-Vβ chains and CD28 on T cells and MHC-II and CD80/CD86 on APCs, leading to cytokine storm and sepsis (64). Significantly we found that, upon SAg-exposure T cells from ibrutinib-treated patients further expanded Cm cells, known to generate long-term responses (88), but these and other memory (*i.e.*, Eff/Em and TEMRA) T cells equally acquired an exhaustion-phenotype (TIM-3+/PD-1+). Notably, there was no need to re-introduce ibrutinib [an irreversible inhibitor (32)] in the cultures, as the drug administration *in vivo* produced significant long-lasting impacts on T cells.

Given that ibrutinib inhibits Itk/TCR-signalling (32, 33), the preferential accrual of Cm cells agrees with previous evidence that lowering functional avidity in T cells enhances long-term memory formation (89–91). The trend to accumulate Cm was apparent also without stimulation, in ibrutinib-treated patient-derived cultures (Nil conditions of **Figure 2B**). This could point at a potential stronger survival of T cells under Itk inhibition, preferentially impacting Cm-phenotype cells, which may consequently accumulate more than other subsets in cultures. This is in keeping with our previous published work showing that inhibition of TCR signals prolongs survival of Cm T cells in the face of chronic cancer Ags (88, 89). Alternatively (or additionally), cancer-Ags stimulation may continue in bulk PBMC cultures from CLL patients (comprising tumour cells) and/or T cells that were Ag-primed *in vivo* continue to expand *in vitro* with a Cm phenotype, a trend more apparent in cultures from ibrutinib-treated patients, especially when TCR stimulants (such as αCD3/28) are re-introduced. This would agree with our previous research showing that T cells sensitized to cancer-Ags *in vivo* proliferate *in vitro* maintaining a Cm-phenotype (92). At a first glance, this Cm accumulation may seem beneficial for CLL patient immunity, as Cm are more durable than Eff/Em T cells (46), in the face of chronic/repeat infections. Such resilience may ephemerally exhaust under the strain of repeat SAg encounters, more quickly in ibrutinib-treated patients.

Contrasting studies reported that ibrutinib treatment did not alter frequency of Tregs in CLL (35), or may even reduce Tregs (38), especially in the initial phases of a *de novo* administration (45) [compatible with early TCR signal inhibition (32)], while Itk ablation associates with stronger Treg responses, at least in mice (93). Given that we did not detect significant differences in Treg induction upon SAg exposure, whether cultures were derived from ibrutinib-treated or untreated CLL patients (**Figure 4**), our data suggest that ibrutinib may not help restore Treg responses of CLL patients to the levels of HDs. While future studies are needed in this respect, we propose that defective Treg responses to SAgs would facilitate unregulated hyperinflammatory responses (94), which cannot be resolved with ibrutinib treatment, supported by our ICS data, increasing risk of severe infection/sepsis in CLL (13). Perhaps, the proportions of cytokine positive cells detected in ICS could simply mirror levels of T cell activation. However, Cm T cell accumulation was highest in HD counterparts (≥60-80%, **Figure 2B**) and this associated with the lowest TNFα/IFNγ responses and the strongest Treg induction, significantly upon TSST-1 exposure (**Figures 5B** and **4B**, respectively). Instead, T cells in cultures from CLL patients did not generate Tregs after SAg-exposure inasmuch as HDs (65, 66) and, irrespectively of ibrutinib treatment, maintained high TNFα/IFNγ cytokine levels. While future studies would be needed to sort specific pseudo-exhausted T cells and perform detailed analyses of cytokine secretion *versus* transcription factor expression, we propose a possible model behind the development of pseudo-exhaustion in CLL (**Figure 8**). Following (or concomitant) with ibrutinib treatment, exposure to environmental SAgs favours the selective expansion of exhaustion-phenotype memory T cells (**Figure 3**). These cells would then maintain cytokine expression (**Figure 5**) correlating with simultaneous defects in Treg induction (**Figure 4**) after SAg-exposure. Previous studies suggested that pseudo-exhaustion (and even reversal of exhaustion) happens over a relatively long course of ibrutinib treatment (37, 40), which would be compatible with repeat exposure to environmental cues. Interestingly, despite significant superior target selectivity of acalabrutinib compared to ibrutinib (95), pseudo-exhausted T cells were reported to diminish from 34.7% to just 28.1% over 6-month therapy with acalabrutinib (96). Thus, future studies are needed to clarify whether SAg T cell responses (and the consequent bystander activation of CLL cells), persist even under acalabrutinib (or other novel BTKi) regimens.

Previously, SEA, SEB, SEC1, SED, and SEE from SA were shown to induce activation and proliferation of CLL cells, dependent on irradiated allogeneic T cell helper cells from HDs (97). Instead, for the first time, we provide evidence that SA SAgs powerfully activate CLL tumour cells via the activation of pseudo-exhausted, autologous T (helper) cells, and potentially even directly, contributing to an hyperinflammatory tumour microenvironment. Despite a significant decline of CLL cells, even ibrutinib-treated patients preserved a fraction of tumour cells producing TNFα, lower than that of untreated CLL patients, but higher than HDs. TNFα secretion in CLL cells was previously associated with worse disease stage (98) and adverse prognosis (99). Additionally, we showed that HLA-DR, CD86 and CD40 substantially increased in CLL cells, upon SAg-exposure, well beyond αCD3/28 T-cell-help controls. High CD86 expression was associated with activation of tumour cells (69), ibrutinib resistance (100) and worse prognosis (101). Consistently, we found another CLL activation marker also associated with negative prognosis (102), CD38 significantly increased after SAg-exposure, promoting CLL cell activation. Intriguingly, SAg (but not αCD3/28) drove downregulation of CD27 expression in CLL cells, suggesting that the CD27 pathway may be directly targeted by SAgs in tumour cells. Moreover, we showed that SAgs upregulated CD40 on CLL cells, while recently CD40 was suggested as a possible receptor of TSST-1, at least on keratinocytes (103). Ibrutinib-treatment effectively reduced the CLL tumour burden and this was associated with lower chances of *in vitro* inflammatory activation of CLL cells in response to SAgs, but we still observed substantial CLL activation in 33% of treated CLL cases. In this context, we did not detect significant differences between activation marker expression of activated CLL cells, whether these were derived from untreated or ibrutinib-treated patients. In comparison, HD-derived B cells remained unaffected by SAgs, suggesting that healthy B cells do not benefit from SAg-driven autologous T cell help as much as CLL cells do. While ibrutinib-treatment would not fully oust the ability of CLL cells to gain from SAg-exposure, future studies are warranted to establish SA SAg-direct effects on CLL cells and whether these help prognosis/diagnoses.

We mostly obtained mutational profile data for the ibrutinib-treated CLL patients, that showed considerable heterogeneity in respect to 13q14 and/or 11q23 deletions and/or *Tp53* mutations, and other variants of unknown significance (such as mutations in *Sf3b1* and *Notch1*). Despite the small cohort size and the limited availability of data about *Ighv* mutational profiles (which can be of prognostic value), our results support the scope for future larger studies aimed at untangling how the heterogeneity of CLL mutations impacts on the responsiveness to staphylococcal SAgs. This may help uncover potential immunoediting dynamics driven by the exposure to environmental cues derived from endemic bacteria.

CLL aetiology remains uncertain. About 30% of CLL patients have stereotyped, quasi-identical BCRs, suggesting the existence of common antigenic factors, including autoantigen or microbial Ags behind the disease (104–106). Infection with viruses [CMV (107, 108) and Epstein-Barr virus (109)] and bacteria [especially infecting the respiratory tract (110–112)], have been proposed as possible causes. While the severity of infection correlates with CLL stage, we provide evidence that staphylococcal SAgs boost the tumour proinflammatory milieu. This highlights the importance of microbial factors in CLL cancer-cell survival and tumour progression. Ultimately, understanding the interplay between infection and CLL progression is crucial for developing more effective targeted therapies. Finally, our study has relevant implications for management of untreated CLL patients given that infection constitutes a relatively major cause of death in this group (after malignancy) (113). These individuals live with CLL for 15-25 years (113–115), during which period they may experience repeat encounters with the endemic SA and its SAgs, that we show have potential to modulate the CLL cells/microenvironment. Considering the abundancy of cancer cells in CLL patients, chronic activation of these cells could heighten the potential for systemic inflammation, thereby exacerbating the risk of severe infections and sepsis, in addition to fostering cancer progression. Thus, our study provides critical evidence for the scope of future larger studies of infection responses in CLL patients.

## Data Availability

The original contributions presented in the study are included in the article/**Supplementary Material**. Further inquiries can be directed to the corresponding author.

## References

[1] Redaelli A Laskin BL Stephens JM Botteman MF Pashos CL. The clinical and epidemiological burden of chronic lymphocytic leukaemia. Eur J Cancer Care. (2004) 13:279–87. doi: 10.1111/j.1365-2354.2004.00489.x 15196232

[2] Smith A Howell D Patmore R Jack A Roman E. Incidence of haematological Malignancy by sub-type: A report from the haematological Malignancy research network. Br J Cancer. (2011) 105:1684–92. doi: 10.1038/bjc.2011.450 PMC3242607 22045184

[3] Yao Y Lin X Li F Jin J Wang H. The global burden and attributable risk factors of chronic lymphocytic leukemia in 204 countries and territories from 1990 to 2019: analysis based on the global burden of disease study 2019. Biomed Eng Online. (2022) 21:4. doi: 10.1186/s12938-021-00973-6 35016695 PMC8753864

[4] Zenz T Mertens D Küppers R Döhner H Stilgenbauer S. From pathogenesis to treatment of chronic lymphocytic leukaemia. Nat Rev Cancer. (2010) 10:37–50. doi: 10.1038/nrc2764 19956173

[5] Nadeu F Delgado J Royo C Baumann T Stankovic T Pinyol M. Clinical impact of clonal and subclonal tp53, sf3b1, birc3, notch1, and atm mutations in chronic lymphocytic leukemia. Blood. (2016) 127:2122–30. doi: 10.1182/blood-2015-07-659144 PMC4912011 26837699

[6] Mansouri L Thorvaldsdottir B Sutton LA Karakatsoulis G Meggendorfer M Parker H. Different prognostic impact of recurrent gene mutations in chronic lymphocytic leukemia depending on ighv gene somatic hypermutation status: A study by Eric in harmony. Leukemia. (2023) 37:339–47. doi: 10.1038/s41375-022-01802-y PMC9898037 36566271

[7] Martínez-Trillos A Quesada V Villamor N Puente XS López-Otín C Campo E. Recurrent gene mutations in cll. Adv Exp Med Biol. (2013) 792:87–107. doi: 10.1007/978-1-4614-8051-8_4 24014293

[8] Xia Y Fan L Wang L Gale RP Wang M Tian T. Frequencies of sf3b1, notch1, myd88, birc3 and ighv mutations and tp53 disruptions in Chinese with chronic lymphocytic leukemia: disparities with Europeans. Oncotarget. (2015) 6:5426–34. doi: 10.18632/oncotarget.3101 PMC4467158 25605254

[9] Goldin LR Slager SL Caporaso NE. Familial chronic lymphocytic leukemia. Curr Opin Hematol. (2010) 17:350–5. doi: 10.1097/MOH.0b013e328338cd99 PMC2891437 20389242

[10] Linet MS Schubauer-Berigan MK Weisenburger DD Richardson DB Landgren O Blair A. Chronic lymphocytic leukaemia: an overview of aetiology in light of recent developments in classification and pathogenesis. Br J Haematol. (2007) 139:672–86. doi: 10.1111/j.1365-2141.2007.06847.x 18021081

[11] Yang S-M Li J-Y Gale RP Huang X-J. The mystery of chronic lymphocytic leukemia (Cll): why is it absent in Asians and what does this tell us about etiology, pathogenesis and biology? Blood Rev. (2015) 29:205–13. doi: 10.1016/j.blre.2014.12.001 25541495

[12] Nosari A. Infectious complications in chronic lymphocytic leukemia. Mediterr J Hematol Infect Dis. (2012) 4:e2012070. doi: 10.4084/mjhid.2012.070 23205258 PMC3507529

[13] Lingamaneni P Farooq MZ Vohra I Upadhyay S Malapati S Singh SRK. Outcomes of patients with chronic lymphocytic leukemia admitted with sepsis: an analysis of national inpatient sample database. Blood. (2019) 134:5460–. doi: 10.1182/blood-2019-131646

[14] Kyle RC Best OG Stephen PM. Immune failure, infection and survival in chronic lymphocytic leukemia. Haematologica. (2018) 103:e329–e. doi: 10.3324/haematol.2018.196543 PMC6029549 29970494

[15] Pauken KE Wherry EJ. Overcoming T cell exhaustion in infection and cancer. Trends Immunol. (2015) 36:265–76. doi: 10.1016/j.it.2015.02.008 PMC4393798 25797516

[16] Zhang Z Liu S Zhang B Qiao L Zhang Y Zhang Y. T cell dysfunction and exhaustion in cancer. Front Cell Dev Biol. (2020) 8:17. doi: 10.3389/fcell.2020.00017 32117960 PMC7027373

[17] Riches JC Davies JK McClanahan F Fatah R Iqbal S Agrawal S. T cells from cll patients exhibit features of T-cell exhaustion but retain capacity for cytokine production. Blood. (2013) 121:1612–21. doi: 10.1182/blood-2012-09-457531 PMC3587324 23247726

[18] Reus B Caserta S Larsen M Morrow G Bano A Hallensleben M. In-depth profiling of T-cell responsiveness to commonly recognized cmv antigens in older people reveals important sex differences. Front Immunol. (2021) 12:707830. doi: 10.3389/fimmu.2021.707830 34484207 PMC8414256

[19] Korona-Glowniak I Grywalska E Grzegorczyk A Roliński J Glowniak A Malm A. Bacterial colonization in patients with chronic lymphocytic leukemia and factors associated with infections and colonization. J Clin Med. (2019) 8:861. doi: 10.3390/jcm8060861 31208150 PMC6616586

[20] Morrison VA. Infectious complications of chronic lymphocytic leukaemia: pathogenesis, spectrum of infection, preventive approaches. Best Pract Res Clin Haematol. (2010) 23:145–53. doi: 10.1016/j.beha.2009.12.004 20620978

[21] Byrd JC Hillmen P O’Brien S Barrientos JC Reddy NM Coutre S. Long-term follow-up of the resonate phase 3 trial of ibrutinib vs ofatumumab. Blood. (2019) 133:2031–42. doi: 10.1182/blood-2018-08-870238 PMC6509542 30842083

[22] Byrd JC Furman RR Coutre SE Flinn IW Burger JA Blum K. Ibrutinib treatment for first-line and relapsed/refractory chronic lymphocytic leukemia: final analysis of the pivotal phase ib/ii pcyc-1102 study. Clin Cancer Res. (2020) 26:3918–27. doi: 10.1158/1078-0432.CCR-19-2856 PMC8175012 32209572

[23] Sharman JP Egyed M Jurczak W Skarbnik A Pagel JM Flinn IW. Acalabrutinib with or without obinutuzumab versus chlorambucil and obinutuzumab for treatment-naive chronic lymphocytic leukaemia (Elevate-tn): A randomised, controlled, phase 3 trial. Lancet. (2020) 395:1278–91. doi: 10.1016/S0140-6736(20)30262-2 PMC8151619 32305093

[24] Tam CS Giannopoulos K Jurczak W Šimkovič M Shadman M Österborg A. Sequoia: results of a phase 3 randomized study of zanubrutinib versus bendamustine + Rituximab (Br) in patients with treatment-naïve (Tn) chronic lymphocytic leukemia/small lymphocytic lymphoma (Cll/sll). Blood. (2021) 138:396–. doi: 10.1182/blood-2021-148457

[25] Shadman M. Diagnosis and treatment of chronic lymphocytic leukemia: A review. Jama. (2023) 329:918–32. doi: 10.1001/jama.2023.1946 36943212

[26] Teh BW Tam CS Handunnetti S Worth LJ Slavin MA. Infections in patients with chronic lymphocytic leukaemia: mitigating risk in the era of targeted therapies. Blood Rev. (2018) 32:499–507. doi: 10.1016/j.blre.2018.04.007 29709246

[27] Douglas AP Trubiano JA Barr I Leung V Slavin MA Tam CS. Ibrutinib may impair serological responses to influenza vaccination. Haematologica. (2017) 102:e397–e9. doi: 10.3324/haematol.2017.164285 PMC5622870 28659336

[28] Andrick B Alwhaibi A DeRemer DL Quershi S Khan R Bryan LJ. Lack of adequate pneumococcal vaccination response in chronic lymphocytic leukaemia patients receiving ibrutinib. Br J Haematol. (2018) 182:712–4. doi: 10.1111/bjh.14855 28737280

[29] Pleyer C Ali MA Cohen JI Tian X Soto S Ahn IE. Effect of bruton tyrosine kinase inhibitor on efficacy of adjuvanted recombinant hepatitis B and zoster vaccines. Blood. (2021) 137:185–9. doi: 10.1182/blood.2020008758 PMC7820878 33259596

[30] Shadman M Ujjani C. Vaccinations in cll: implications for Covid-19. Blood. (2021) 137:144–6. doi: 10.1182/blood.2020009966 PMC7820880 33443562

[31] Bacova B Kohutova Z Zubata I Gaherova L Kucera P Heizer T. Cellular and humoral immune response to Sars-Cov-2 mrna vaccines in patients treated with either ibrutinib or rituximab. Clin Exp Med. (2023) 23:371–9. doi: 10.1007/s10238-022-00809-0 PMC8963888 35352210

[32] Dubovsky JA Beckwith KA Natarajan G Woyach JA Jaglowski S Zhong Y. Ibrutinib is an irreversible molecular inhibitor of itk driving a th1-selective pressure in T lymphocytes. Blood. (2013) 122:2539–49. doi: 10.1182/blood-2013-06-507947 PMC3795457 23886836

[33] Shah K Al-Haidari A Sun J Kazi JU. T cell receptor (Tcr) signaling in health and disease. Signal Transduction Targeted Ther. (2021) 6:412. doi: 10.1038/s41392-021-00823-w PMC8666445 34897277

[34] Niemann CU Herman SE Maric I Gomez-Rodriguez J Biancotto A Chang BY. Disruption of *in vivo* chronic lymphocytic leukemia tumor-microenvironment interactions by ibrutinib–findings from an investigator-initiated phase ii study. Clin Cancer Res. (2016) 22:1572–82. doi: 10.1158/1078-0432.Ccr-15-1965 PMC4818677 26660519

[35] Long M Beckwith K Do P Mundy BL Gordon A Lehman AM. Ibrutinib treatment improves T cell number and function in cll patients. J Clin Invest. (2017) 127:3052–64. doi: 10.1172/jci89756 PMC5531425 28714866

[36] Davis JE Sharpe C Mason K Tam CS Koldej RM Ritchie DS. Ibrutinib protects T cells in patients with cll from proliferation-induced senescence. J Trans Med. (2021) 19:473. doi: 10.1186/s12967-021-03136-2 PMC8609739 34809665

[37] Solman IG Blum LK Burger JA Kipps TJ Dean JP James DF. Impact of long-term ibrutinib treatment on circulating immune cells in previously untreated chronic lymphocytic leukemia. Leukemia Res. (2021) 102:106520. doi: 10.1016/j.leukres.2021.106520 33611131

[38] Solman IG Blum LK Hoh HY Kipps TJ Burger JA Barrientos JC. Ibrutinib restores immune cell numbers and function in first-line and relapsed/refractory chronic lymphocytic leukemia. Leuk Res. (2020) 97:106432. doi: 10.1016/j.leukres.2020.106432 32911375

[39] Fraietta JA Beckwith KA Patel PR Ruella M Zheng Z Barrett DM. Ibrutinib enhances chimeric antigen receptor T-cell engraftment and efficacy in leukemia. Blood. (2016) 127:1117–27. doi: 10.1182/blood-2015-11-679134 PMC4778162 26813675

[40] Parry HM Mirajkar N Cutmore N Zuo J Long H Kwok M. Long-term ibrutinib therapy reverses cd8+ T cell exhaustion in B cell chronic lymphocytic leukaemia. Front Immunol. (2019) 10:2832. doi: 10.3389/fimmu.2019.02832 31921116 PMC6921985

[41] Cubillos-Zapata C Avendaño-Ortiz J Córdoba R Hernández-Jiménez E Toledano V Pérez de Diego R. Ibrutinib as an antitumor immunomodulator in patients with refractory chronic lymphocytic leukemia. OncoImmunology. (2016) 5:e1242544. doi: 10.1080/2162402X.2016.1242544 28123879 PMC5213769

[42] Robinson HR Qi J Cook EM Nichols C Dadashian EL Underbayev C. A cd19/cd3 bispecific antibody for effective immunotherapy of chronic lymphocytic leukemia in the ibrutinib era. Blood. (2018) 132:521–32. doi: 10.1182/blood-2018-02-830992 PMC6073325 29743179

[43] Fan F Yoo HJ Stock S Wang L Liu Y Schubert M-L. Ibrutinib for improved chimeric antigen receptor T-cell production for chronic lymphocytic leukemia patients. Int J Cancer. (2021) 148:419–28. doi: 10.1002/ijc.33212 32683672

[44] Gribben JG. No longer too exhausted to run. Blood. (2018) 132:464–5. doi: 10.1182/blood-2018-06-851642 PMC6073321 30072413

[45] Podhorecka M Goracy A Szymczyk A Kowal M Ibanez B Jankowska-Lecka O. Changes in T-cell subpopulations and cytokine network during early period of ibrutinib therapy in chronic lymphocytic leukemia patients: the significant decrease in T regulatory cells number. Oncotarget. (2017) 8:34661–9. doi: 10.18632/oncotarget.16148 PMC5471000 28416773

[46] Hope JL Stairiker CJ Bae E-A Otero DC Bradley LM. Striking a balance—Cellular and molecular drivers of memory T cell development and responses to chronic stimulation. Front Immunol. (2019) 10:1595. doi: 10.3389/fimmu.2019.01595 31379821 PMC6650570

[47] Byrd JC Brown JR O’Brien S Barrientos JC Kay NE Reddy NM. Ibrutinib versus ofatumumab in previously treated chronic lymphoid leukemia. New Engl J Med. (2014) 371:213–23. doi: 10.1056/NEJMoa1400376 PMC4134521 24881631

[48] Burger JA Tedeschi A Barr PM Robak T Owen C Ghia P. Ibrutinib as initial therapy for patients with chronic lymphocytic leukemia. New Engl J Med. (2015) 373:2425–37. doi: 10.1056/NEJMoa1509388 PMC4722809 26639149

[49] O’Brien S Furman RR Coutre SE Sharman JP Burger JA Blum KA. Ibrutinib as initial therapy for elderly patients with chronic lymphocytic leukaemia or small lymphocytic lymphoma: an open-label, multicentre, phase 1b/2 trial. Lancet Oncol. (2014) 15:48–58. doi: 10.1016/S1470-2045(13)70513-8 24332241 PMC4134524

[50] O’Brien S Jones JA Coutre SE Mato AR Hillmen P Tam C. Ibrutinib for patients with relapsed or refractory chronic lymphocytic leukaemia with 17p deletion (Resonate-17): A phase 2, open-label, multicentre study. Lancet Oncol. (2016) 17:1409–18. doi: 10.1016/S1470-2045(16)30212-1 27637985

[51] Farooqui MZH Valdez J Martyr S Aue G Saba N Niemann CU. Ibrutinib for previously untreated and relapsed or refractory chronic lymphocytic leukaemia with tp53 aberrations: A phase 2, single-arm trial. Lancet Oncol. (2015) 16:169–76. doi: 10.1016/S1470-2045(14)71182-9 PMC4342187 25555420

[52] Byrd JC Woyach JA Furman RR Martin P O’Brien S Brown JR. Acalabrutinib in treatment-naive chronic lymphocytic leukemia. Blood. (2021) 137:3327–38. doi: 10.1182/blood.2020009617 PMC8670015 33786588

[53] Ghia P Pluta A Wach M Lysak D Kozak T Simkovic M. Ascend: phase iii, randomized trial of acalabrutinib versus idelalisib plus rituximab or bendamustine plus rituximab in relapsed or refractory chronic lymphocytic leukemia. J Clin Oncol. (2020) 38:2849–61. doi: 10.1200/jco.19.03355 32459600

[54] Sharman JP Egyed M Jurczak W Skarbnik A Pagel JM Flinn IW. Efficacy and safety in a 4-year follow-up of the elevate-tn study comparing acalabrutinib with or without obinutuzumab versus obinutuzumab plus chlorambucil in treatment-naïve chronic lymphocytic leukemia. Leukemia. (2022) 36:1171–5. doi: 10.1038/s41375-021-01485-x PMC8979808 34974526

[55] Tam CS Dimopoulos M Garcia-Sanz R Trotman J Opat S Roberts AW. Pooled safety analysis of zanubrutinib monotherapy in patients with B-cell Malignancies. Blood Adv. (2022) 6:1296–308. doi: 10.1182/bloodadvances.2021005621 PMC8864647 34724705

[56] Oliveira D Borges A Simões M. Staphylococcus aureus toxins and their molecular activity in infectious diseases. Toxins. (2018) 10. doi: 10.3390/toxins10060252 PMC6024779 29921792

[57] Azuma K Koike K Kobayashi T Mochizuki T Mashiko K Yamamoto Y. Detection of circulating superantigens in an intensive care unit population. Int J Infect Diseases: IJID. (2004) 8:292–8. doi: 10.1016/j.ijid.2003.12.005 15325598

[58] Ferry T Thomas D Genestier A-L Bes M Lina G Vandenesch F. Comparative prevalence of superantigen genes in staphylococcus aureus isolates causing sepsis with and without septic shock. Clin Infect Dis. (2005) 41:771–7. doi: 10.1086/432798 16107972

[59] Tuffs SW Goncheva MI Xu SX Craig HC Kasper KJ Choi J. Superantigens promote *Staphylococcus aureus* bloodstream infection by eliciting pathogenic interferon-gamma production. Proc Natl Acad Sci. (2022) 119:e2115987119. doi: 10.1073/pnas.2115987119 35165181 PMC8872782

[60] Salgado-Pabón W Breshears L Spaulding AR Merriman JA Stach CS Horswill AR. Superantigens are critical for staphylococcus aureus infective endocarditis, sepsis, and acute kidney injury. mBio. (2013) 4:00494–13. doi: 10.1128/mbio.00494-13 PMC3747586 23963178

[61] Naylor-Adamson L Chacko AR Booth Z Caserta S Jarvis J Khan S. Bruton’s tyrosine kinase inhibitors impair fcgammariia-driven platelet responses to bacteria in chronic lymphocytic leukemia. Front Immunol. (2021) 12:766272. doi: 10.3389/fimmu.2021.766272 34912339 PMC8667317

[62] Sallusto F Lenig D Förster R Lipp M Lanzavecchia A. Two subsets of memory T lymphocytes with distinct homing potentials and effector functions. Nature. (1999) 402:34–8. doi: 10.1038/35005534 10537110

[63] Palma M Gentilcore G Heimersson K Mozaffari F Näsman-Glaser B Young E. T cells in chronic lymphocytic leukemia display dysregulated expression of immune checkpoints and activation markers. Haematologica. (2017) 102:562–72. doi: 10.3324/haematol.2016.151100 PMC5394965 27927767

[64] Ramachandran G. Gram-positive and gram-negative bacterial toxins in sepsis: A brief review. Virulence. (2014) 5:213–8. doi: 10.4161/viru.27024 PMC3916377 24193365

[65] Taylor AL Llewelyn MJ. Superantigen-induced proliferation of human cd4+Cd25- T cells is followed by a switch to a functional regulatory phenotype. J Immunol (Baltimore Md: 1950). (2010) 185:6591–8. doi: 10.4049/jimmunol.1002416 21048104

[66] Taylor AL Cross EL Llewelyn MJ. Induction of contact-dependent cd8(+) regulatory T cells through stimulation with staphylococcal and streptococcal superantigens. Immunology. (2012) 135:158–67. doi: 10.1111/j.1365-2567.2011.03529.x PMC3277718 22043981

[67] Iglesias-Escudero M Arias-Gonzalez N Martinez-Caceres E. Regulatory cells and the effect of cancer immunotherapy. Mol Cancer. (2023) 22:26. doi: 10.1186/s12943-023-01714-0 36739406 PMC9898962

[68] Honigberg LA Smith AM Sirisawad M Verner E Loury D Chang B. The Bruton tyrosine kinase inhibitor pci-32765 blocks B-cell activation and is efficacious in models of autoimmune disease and B-cell Malignancy. Proc Natl Acad Sci U.S.A. (2010) 107:13075–80. doi: 10.1073/pnas.1004594107 PMC2919935 20615965

[69] Huemer M Rebhandl S Zaborsky N Gassner FJ Hainzl S Weiss L. Aid induces intraclonal diversity and genomic damage in cd86(+) chronic lymphocytic leukemia cells. Eur J Immunol. (2014) 44:3747–57. doi: 10.1002/eji.201344421 PMC4276288 25179679

[70] Grimsholm O. Cd27 on human memory B cells-more than just a surface marker. Clin Exp Immunol. (2023) 213:164–72. doi: 10.1093/cei/uxac114 PMC10361737 36508329

[71] Kaspersen KA Dinh KM Erikstrup LT Burgdorf KS Pedersen OB Sørensen E. Low-grade inflammation is associated with susceptibility to infection in healthy men: results from the Danish blood donor study (Dbds). PloS One. (2016) 11:e0164220. doi: 10.1371/journal.pone.0164220 27701463 PMC5049789

[72] Tong SYC Davis JS Eichenberger E Holland TL Fowler VG. Staphylococcus aureus infections: epidemiology, pathophysiology, clinical manifestations, and management. Clin Microbiol Rev. (2015) 28:603–61. doi: 10.1128/cmr.00134-14 PMC4451395 26016486

[73] Kourtis AP Hatfield K Baggs J Mu Y See I Epson E. Vital signs: epidemiology and recent trends in methicillin-resistant and in methicillin-susceptible Staphylococcus aureus bloodstream infections - United States. MMWR Morbidity Mortality Weekly Rep. (2019) 68:214–9. doi: 10.15585/mmwr.mm6809e1 PMC6421967 30845118

[74] Caraballo C Jaimes F. Organ dysfunction in sepsis: an ominous trajectory from infection to death. Yale J Biol Med. (2019) 92:629–40. PMC6913810 31866778

[75] Blez D Blaize M Soussain C Boissonnas A Meghraoui-Kheddar A Menezes N. Ibrutinib induces multiple functional defects in the neutrophil response against Aspergillus fumigatus. Haematologica. (2020) 105:478–89. doi: 10.3324/haematol.2019.219220 PMC7012467 31171644

[76] Fiorcari S Maffei R Vallerini D Scarfo L Barozzi P Maccaferri M. Btk inhibition impairs the innate response against fungal infection in patients with chronic lymphocytic leukemia. Front Immunol. (2020) 11:2158. doi: 10.3389/fimmu.2020.02158 32983178 PMC7485008

[77] Gomez-Rodriguez J Kraus ZJ Schwartzberg PL. Tec family kinases itk and rlk/txk in T lymphocytes: cross-regulation of cytokine production and T-cell fates. FEBS J. (2011) 278:1980–9. doi: 10.1111/j.1742-4658.2011.08072.x PMC3117960 21362139

[78] Moreno C Munoz C Terol MJ Hernandez-Rivas JA Villanueva M. Restoration of the immune function as a complementary strategy to treat chronic lymphocytic leukemia effectively. J Exp Clin Cancer Res. (2021) 40:321. doi: 10.1186/s13046-021-02115-1 34654437 PMC8517318

[79] Liu Y Song Y Yin Q. Effects of ibrutinib on T-cell immunity in patients with chronic lymphocytic leukemia. Front Immunol. (2022) 13:962552. doi: 10.3389/fimmu.2022.962552 36059445 PMC9437578

[80] Greil R Tedeschi A Moreno C Anz B Larratt L Simkovic M. Pretreatment with ibrutinib reduces cytokine secretion and limits the risk of obinutuzumab-induced infusion-related reactions in patients with cll: analysis from the illuminate study. Ann Hematol. (2021) 100:1733–42. doi: 10.1007/s00277-021-04536-6 PMC8195966 34018029

[81] Breton G Chomont N Takata H Fromentin R Ahlers J Filali-Mouhim A. Programmed death-1 is a marker for abnormal distribution of naive/memory T cell subsets in hiv-1 infection. J Immunol (Baltimore Md: 1950). (2013) 191:2194–204. doi: 10.4049/jimmunol.1200646 PMC3815464 23918986

[82] Day CL Abrahams DA Bunjun R Stone L de Kock M Walzl G. Pd-1 expression on mycobacterium tuberculosis-specific cd4 T cells is associated with bacterial load in human tuberculosis. Front Immunol. (2018) 9:1995. doi: 10.3389/fimmu.2018.01995 30233588 PMC6127207

[83] Waki K Yamada T Yoshiyama K Terazaki Y Sakamoto S Matsueda S. Pd-1 expression on peripheral blood T-cell subsets correlates with prognosis in non-small cell lung cancer. Cancer Sci. (2014) 105:1229–35. doi: 10.1111/cas.12502 PMC4462362 25117757

[84] Gattinoni L Lugli E Ji Y Pos Z Paulos CM Quigley MF. A human memory T cell subset with stem cell-like properties. Nat Med. (2011) 17:1290–7. doi: 10.1038/nm.2446 PMC3192229 21926977

[85] Arruga F Gyau BB Iannello A Vitale N Vaisitti T Deaglio S. Immune response dysfunction in chronic lymphocytic leukemia: dissecting molecular mechanisms and microenvironmental conditions. Int J Mol Sci. (2020) 21:1825. doi: 10.3390/ijms21051825 32155826 PMC7084946

[86] Görgün G Holderried TAW Zahrieh D Neuberg D Gribben JG. Chronic lymphocytic leukemia cells induce changes in gene expression of cd4 and cd8 T cells. J Clin Invest. (2005) 115:1797–805. doi: 10.1172/JCI24176 PMC1150284 15965501

[87] Kabanova A Sanseviero F Candi V Gamberucci A Gozzetti A Campoccia G. Human cytotoxic T lymphocytes form dysfunctional immune synapses with B cells characterized by non-polarized lytic granule release. Cell Rep. (2016) 15:9–18. doi: 10.1016/j.celrep.2016.02.084 27052167

[88] Caserta S Borger JG Zamoyska R. Central and effector memory cd4 and cd8 T-cell responses to tumor-associated antigens. Crit Rev Immunol. (2012) 32:97–126. doi: 10.1615/critrevimmunol.v32.i2.10 23216610

[89] Caserta S Kleczkowska J Mondino A Zamoyska R. Reduced functional avidity promotes central and effector memory cd4 T cell responses to tumor-associated antigens. J Immunol (Baltimore Md: 1950). (2010) 185:6545–54. doi: 10.4049/jimmunol.1001867 21048115

[90] Araki K Turner AP Shaffer VO Gangappa S Keller SA Bachmann MF. Mtor regulates memory cd8 T-cell differentiation. Nature. (2009) 460:108–12. doi: 10.1038/nature08155 PMC2710807 19543266

[91] Bushar ND Corbo E Schmidt M Maltzman JS Farber DL. Ablation of slp-76 signaling after T cell priming generates memory cd4 T cells impaired in steady-state and cytokine-driven homeostasis. Proc Natl Acad Sci U.S.A. (2010) 107:827–31. doi: 10.1073/pnas.0908126107 PMC2818906 20080760

[92] Caserta S Alessi P Basso V Mondino A. Il-7 is superior to il-2 for *ex vivo* expansion of tumour-specific cd4+ T cells. Eur J Immunol. (2010) 40:470–9. doi: 10.1002/eji.200939801 19950184

[93] Gomez-Rodriguez J Wohlfert EA Handon R Meylan F Wu JZ Anderson SM. Itk-mediated integration of T cell receptor and cytokine signaling regulates the balance between th17 and regulatory T cells. J Exp Med. (2014) 211:529–43. doi: 10.1084/jem.20131459 PMC3949578 24534190

[94] Venet F Chung CS Monneret G Huang X Horner B Garber M. Regulatory T cell populations in sepsis and trauma. J Leukoc Biol. (2008) 83:523–35. doi: 10.1189/jlb.0607371 17913974

[95] Byrd JC Harrington B O’Brien S Jones JA Schuh A Devereux S. Acalabrutinib (Acp-196) in relapsed chronic lymphocytic leukemia. New Engl J Med. (2016) 374:323–32. doi: 10.1056/NEJMoa1509981 PMC4862586 26641137

[96] Byrd JC Wierda WG Schuh A Devereux S Chaves JM Brown JR. Acalabrutinib monotherapy in patients with relapsed/refractory chronic lymphocytic leukemia: updated phase 2 results. Blood. (2020) 135:1204–13. doi: 10.1182/blood.2018884940 PMC7146022 31876911

[97] Duan X Nerl C Janssen O Kabelitz D. B-cell maturation in chronic lymphocytic leukaemia. Iv. T-cell-dependent activation of leukaemic B cells by staphylococcal enterotoxin ‘Superantigens’. Immunology. (1992) 75:420–6. PMC1384734 1572690

[98] Foa R Massaia M Cardona S Tos AG Bianchi A Attisano C. Production of tumor necrosis factor-alpha by B-cell chronic lymphocytic leukemia cells: A possible regulatory role of tnf in the progression of the disease. Blood. (1990) 76:393–400. doi: 10.1182/blood.V76.2.393.bloodjournal762393 2114936

[99] Bojarska-Junak A Hus I Szczepanek EW Dmoszynska A Rolinski J. Peripheral blood and bone marrow tnf and tnf receptors in early and advanced stages of B-cll in correlation with zap-70 protein and cd38 antigen. Leuk Res. (2008) 32:225–33. doi: 10.1016/j.leukres.2007.06.007 17675228

[100] Takacs F Kotmayer L Czeti A Szaloki G Laszlo T Mikala G. Revealing a phenotypical appearance of ibrutinib resistance in patients with chronic lymphocytic leukaemia by flow cytometry. Pathol Oncol Res. (2022) 28:1610659. doi: 10.3389/pore.2022.1610659 36213161 PMC9532522

[101] Takacs F Tolnai-Kriston C Hernadfoi M Szabo O Szaloki G Szepesi A. The effect of cd86 expression on the proliferation and the survival of cll cells. Pathol Oncol Res. (2019) 25:647–52. doi: 10.1007/s12253-018-0512-7 30406401

[102] Malavasi F Deaglio S Damle R Cutrona G Ferrarini M Chiorazzi N. Cd38 and chronic lymphocytic leukemia: A decade later. Blood. (2011) 118:3470–8. doi: 10.1182/blood-2011-06-275610 PMC3574275 21765022

[103] Schlievert PM Gourronc FA Leung DYM Klingelhutz AJ. Human keratinocyte response to superantigens. mSphere. (2020) 5. doi: 10.1128/mSphere.00803-20 PMC7568652 33028686

[104] Ghiotto F Fais F Valetto A Albesiano E Hashimoto S Dono M. Remarkably similar antigen receptors among a subset of patients with chronic lymphocytic leukemia. J Clin Invest. (2004) 113:1008–16. doi: 10.1172/JCI19399 PMC379317 15057307

[105] Ten Hacken E Gounari M Ghia P Burger JA. The importance of B cell receptor isotypes and stereotypes in chronic lymphocytic leukemia. Leukemia. (2019) 33:287–98. doi: 10.1038/s41375-018-0303-x PMC7182338 30555163

[106] Hamblin T. Is chronic lymphocytic leukemia a response to infectious agents? Leuk Res. (2006) 30:1063–4. doi: 10.1016/j.leukres.2005.11.022 16406017

[107] Kipps TJ Tomhave E Chen PP Carson DA. Autoantibody-associated kappa light chain variable region gene expressed in chronic lymphocytic leukemia with little or no somatic mutation. Implications Etiol Immunother J Exp Med. (1988) 167:840–52. doi: 10.1084/jem.167.3.840 PMC2188892 3127527

[108] Steininger C Widhopf GF 2nd Ghia EM Morello CS Vanura K Sanders R. Recombinant antibodies encoded by ighv1-69 react with pul32, a phosphoprotein of cytomegalovirus and B-cell superantigen. Blood. (2012) 119:2293–301. doi: 10.1182/blood-2011-08-374058 PMC3311256 22234695

[109] Kostareli E Hadzidimitriou A Stavroyianni N Darzentas N Athanasiadou A Gounari M. Molecular evidence for ebv and cmv persistence in a subset of patients with chronic lymphocytic leukemia expressing stereotyped ighv4-34 B-cell receptors. Leukemia. (2009) 23:919–24. doi: 10.1038/leu.2008.379 19148139

[110] Landgren O Gridley G Check D Caporaso NE Morris Brown L. Acquired immune-related and inflammatory conditions and subsequent chronic lymphocytic leukaemia. Br J Haematol. (2007) 139:791–8. doi: 10.1111/j.1365-2141.2007.06859.x 17941950

[111] Landgren O Rapkin JS Caporaso NE Mellemkjaer L Gridley G Goldin LR. Respiratory tract infections and subsequent risk of chronic lymphocytic leukemia. Blood. (2007) 109:2198–201. doi: 10.1182/blood-2006-08-044008 PMC1801057 17082317

[112] Lanemo Myhrinder A Hellqvist E Sidorova E Soderberg A Baxendale H Dahle C. A new perspective: molecular motifs on oxidized ldl, apoptotic cells, and bacteria are targets for chronic lymphocytic leukemia antibodies. Blood. (2008) 111:3838–48. doi: 10.1182/blood-2007-11-125450 18223168

[113] Wierda WG O’Brien S Wang X Faderl S Ferrajoli A Do K-A. Prognostic nomogram and index for overall survival in previously untreated patients with chronic lymphocytic leukemia. Blood. (2007) 109:4679–85. doi: 10.1182/blood-2005-12-051458 17299097

[114] Condoluci A Terzi di Bergamo L Langerbeins P Hoechstetter MA Herling CD De Paoli L. International prognostic score for asymptomatic early-stage chronic lymphocytic leukemia. Blood. (2020) 135:1859–69. doi: 10.1182/blood.2019003453 PMC11311630 32267500

[115] Brieghel C Galle V Agius R da-Cunha-Bang C Andersen MA Vlummens P. Identifying patients with chronic lymphocytic leukemia without need of treatment: end of endless watch and wait? Eur J Haematol. (2022) 108:369–78. doi: 10.1111/ejh.13743 35030282

