# Supplementary material for: Ibrutinib enhances the bias of T cell responses towards staphylococcal superantigens sustaining inflammation in chronic lymphocytic leukaemia
Source: Front Immunol. 2025 Mar 26;16:1531059. doi: 10.3389/fimmu.2025.1531059 (PMC11978837; doi:10.3389/fimmu.2025.1531059)
Supplement: Supplementary file 1 [file DataSheet1.pdf]

Ibrutinib enhances the bias of T-cell responses towards staphylococcal superantigens sustaining inflammation in chronic lymphocytic leukaemia

Fisal Tantoush[1§], David Allsup[1,2], Leigh Naylor-Adamson[1], Frank Voncken[1] and Stefano Caserta[1*]

[1]Centre for Biomedicine, Biomedical Institute for Multimorbidity, Hull York Medical School, University of Hull, Hull, United Kingdom, HU6 7RX; [2]Department of Haematology, Castle Hill Hospital, Hull University Teaching Hospital NHS Trust, Hull, United Kingdom.

[§]Present address: Brighton and Sussex Medical School, The University of Sussex, Falmer, East Sussex, United Kingdom, BN1 9PX.

*Corresponding author

Correspondence to: Stefano Caserta, Centre for Biomedicine, Biomedical Institute for Multimorbidity, Hull York Medical School, Faculty of Health Sciences, University of Hull, United Kingdom, HU6 7RX, UK Phone: +44-(0)1482-463451; Fax: +44-(0); E-mail: Stefano.Caserta@hyms.ac.uk

Supplementary Material Contents:

1. Supplementary Figure 1 with Legend pages 3-5
2. Supplementary Figure 2 with Legend pages 6-7
3. Supplementary Figure 3 with Legend pages 8-10

4. Supplementary Figure 4 with Legend pages 11-12

5. Supplementary Figure 5 with Legend pages 13-14

6. Supplementary Figure 6 with Legend pages 15-16

7. Supplementary Figure 7 with Legend pages 17-18

8. Supplementary Figure 8 with Legend pages 19-20

9. Supplementary Figure 9 with Legend pages 21-22

10. Supplementary Figure 10 with Legend pages 23-24

11. Supplementary Figure 11 with Legend pages 25-26

12. Supplementary Figure 12 with Legend pages 27-28

13. Supplementary Figure 13 with Legend pages 29-30

14. Supplementary Figure 14 with Legend pages 31-32

15. Supplementary Figure 15 with Legend pages 33-34

16. Supplementary Figure 16 with Legend pages 35-36

17. Supplementary Figure 17 with Legend pages 37-38

18. Supplementary Figure 18 with Legend pages 39-40

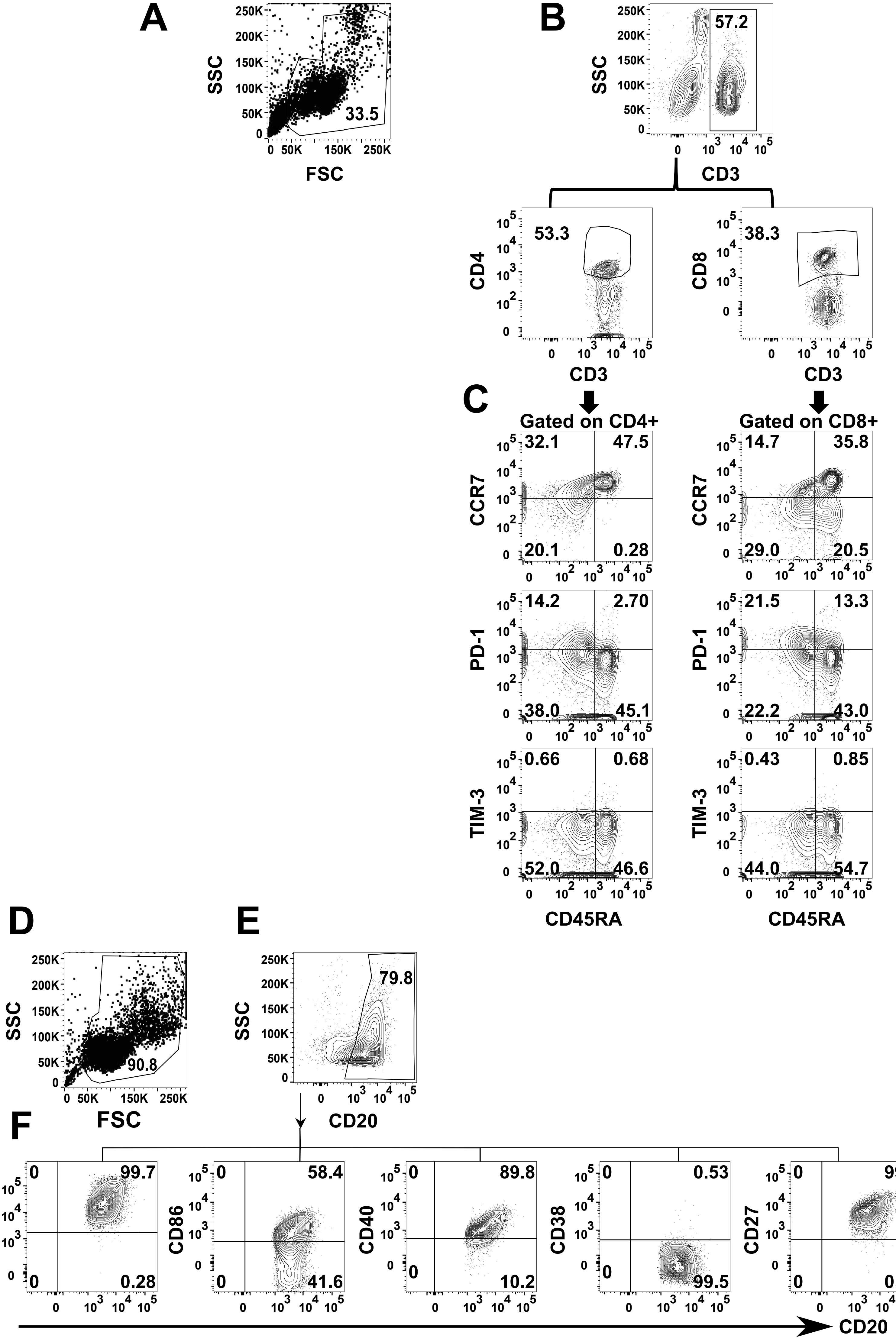
A
SSC
FSC
33.5
B
SSC
CD3
57.2
CD4
CD3
53.3
CD8
CD3
38.3
C
Gated on CD4+
Gated on CD8+
CCR7
CD45RA
32.1
47.5
20.1
0.28
14.7
35.8
29.0
20.5
PD-1
14.2
2.70
38.0
45.1
21.5
13.3
22.2
43.0
TIM-3
0.66
0.68
52.0
46.6
0.43
0.85
44.0
54.7
D
SSC
FSC
90.8
E
SSC
CD20
79.8
F
HLA-DR
0
99.7
0
0.28
CD86
0
58.4
0
41.6
CD40
0
89.8
0
10.2
CD38
0
0.53
0
99.5
CD27
0
99.2
0
0.76
CD20

Supplementary Figure 1. Gating strategy used in the phenotypic analyses of T and B/CLL tumour cells. Freshly isolated, viable PBMCs (as by Trypan Blue exclusion assay; $2x10^6$) derived from HDs or CLL patients were incubated with anti-FcR mAb and surface-stained with mAbs directed to the indicated surface markers in T (A-C) and B (D-F) cell flow-cytometry panels, as detailed in the Materials and Methods. Samples were then fixed and acquired on a BD LSR II Fortessa, setting up strictly defined rainbow profiles specific for each panel (required to remove day-to-day variability across the patient/donor recruitment period), and finally analysed using FlowJo™ software. **A)** The dot plot shows the viable lymphocyte gate by forward and side scatter (FSC *vs* SSC; *i.e.* size *vs* granularity) in HD, HDY-27M-013 sample (T cell panel), as example. **B)** The top contour plot shows how $CD3^+$ T cells were identified in HDY-27M-013, by showing CD3 expression against SSC within gated viable lymphocytes. Thereafter, $CD4^+$ $CD3^+$ (left contour plot) and $CD8^+$ $CD3^+$ (right contour plot) T cells were identified in gated $CD3^+$ viable T cells. **C)** Left and right contour plots respectively show the analysis of $CD4^+$ and $CD8^+$ T cells for the identification of: (i) naïve and memory T cell subsets using well known markers, CCR7 and CD45RA (top row), defining: naïve-like (Tn) $CCR7^+CD45RA^+$; central memory (Cm) $CCR7^+CD45RA^-$; effectors/effector memory (Eff/Em) $CCR7^-CD45RA^-$, and TEMRA (Temra) $CCR7^-CD45RA^+$ gates and (ii) the expression of checkpoint receptors: PD-1 (middle row) and TIM-3 (bottom row), gated against the memory marker CD45RA. **D)** Dot plot shows the viable lymphocyte gate by FSC *vs* SSC in an example CLL patient (P387, untreated CLL) for the B cell panel. **E)** Contour plot illustrates gated CLL tumour cells by showing CD20 expression against SSC within gated viable lymphocytes. **F)** Contour plots respectively show the expression of B cell activation/antigen-presentation marker, HLA-DR; co-stimulation markers, CD86 and

CD40; memory marker, CD27, and CLL negative prognosis marker, CD38, in gated viable $CD20^+$ CLL tumour cells. The gates shown were used consistently across all samples analysed in the study.

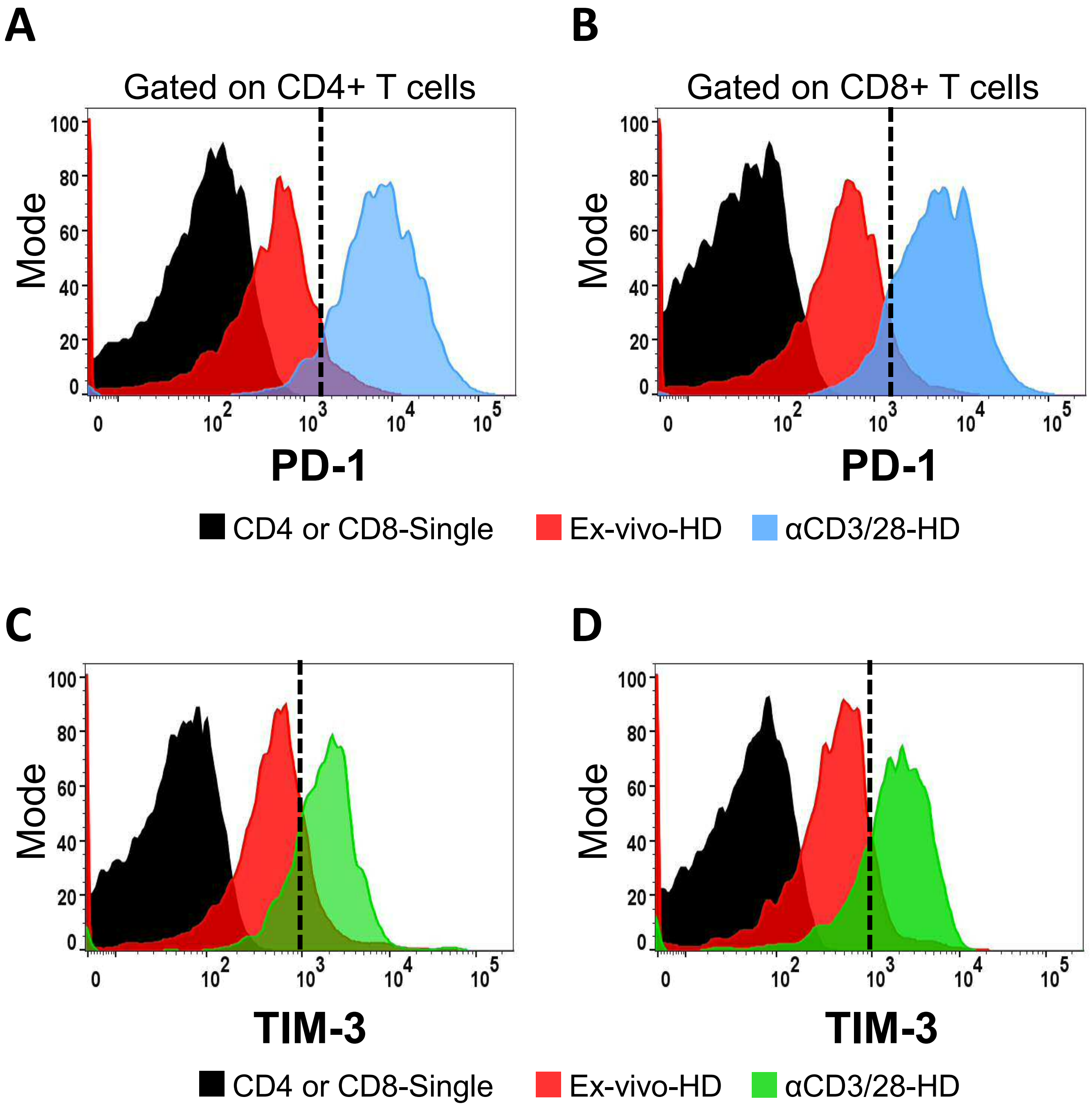
A
B
Gated on CD4+ T cells
Gated on CD8+ T cells
Mode
PD-1
CD4 or CD8-Single
Ex-vivo-HD
αCD3/28-HD
C
D
TIM-3

Supplementary Figure 2. Validation of PD-1+ and TIM-3+ gating strategy for CD4$^+$ and CD8$^+$ T cells. PBMCs from an example HD (HDY-33F-015) were immunophenotyped *ex vivo* (red shaded histograms) or after stimulation with anti-CD3 and anti-CD28 crosslinking monoclonal antibodies (αCD3/28 mAbs; light blue and green shaded histograms), as described in the Materials and Methods for five days. Then, cells were surface-stained with anti-CD3, anti-CD4, anti-PD-1, anti-TIM-3, anti-CD45RA, and anti-CCR7 mAbs prior to acquisition. Viable T cells from *ex-vivo* and day 5 acquisitions were further gated on CD3+ and CD4+ events (A, C) or CD3+ and CD8+ events (B, D), following the gating shown in Supplementary Figure 1A-B. **A-B**) Flow cytometric histogram overlays show PD-1 gating threshold (dashed line) in CD4$^+$ (A) and CD8$^+$ (B) T cells comparing expression *ex vivo* (red shaded histograms), after αCD3/28 stimulation (light blue shaded histograms) relative to CD4 (A) and CD8 (B) single staining controls (black histograms). **C-D**) Flow cytometric histogram overlays show TIM-3 gating threshold (dashed line) in CD4$^+$ (C) and CD8$^+$ (D) T cells comparing expression *ex vivo* (red shaded histograms), after αCD3/28 stimulation (green shaded histograms) relative to CD4 (C) and CD8 (D) single controls (black histograms). The gates shown were used consistently across all samples analysed in the study.

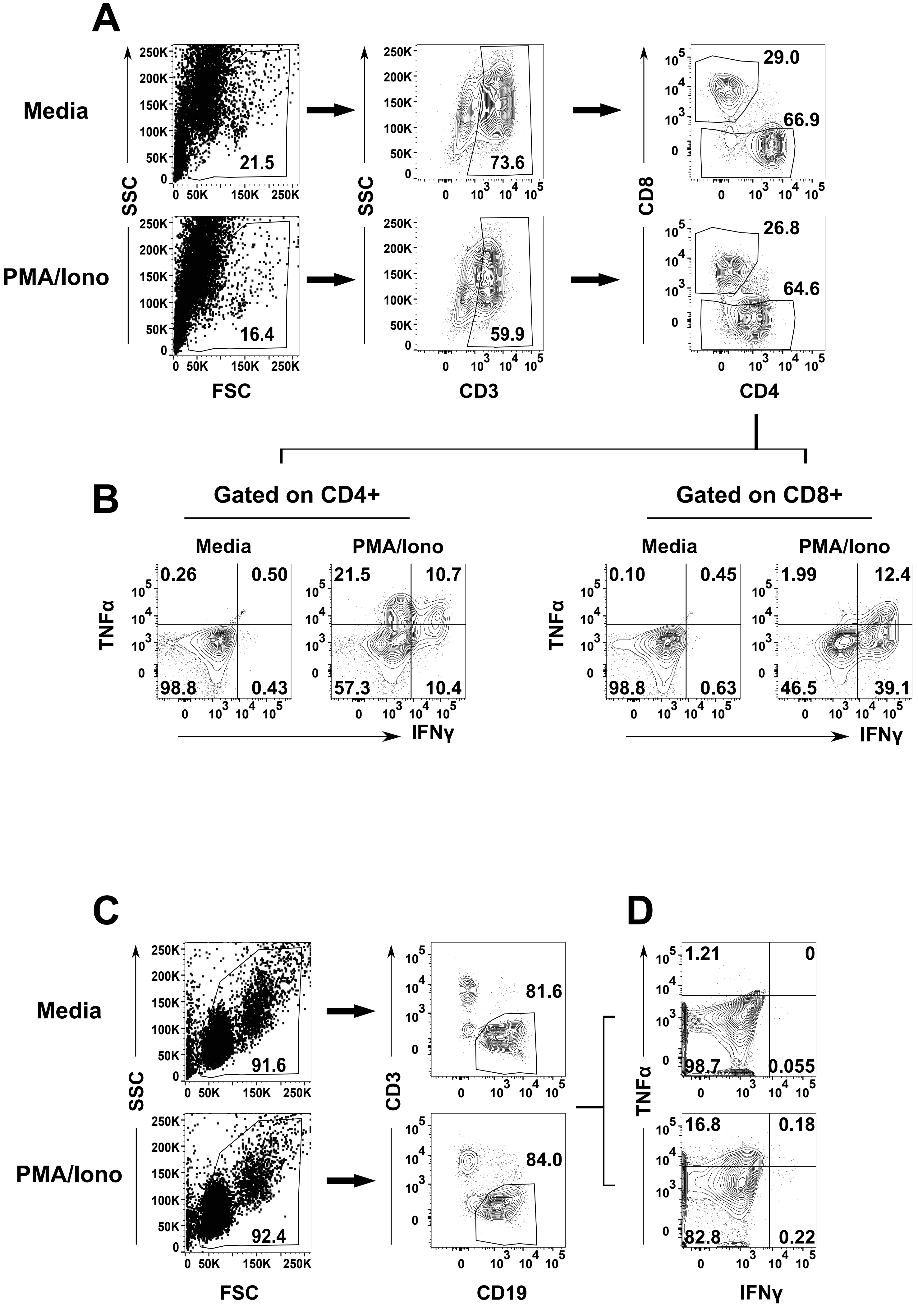

A
Media
PMA/Iono
SSC
FSC
21.5
16.4
73.6
59.9
CD3
CD8
CD4
29.0
66.9
26.8
64.6
B
Gated on CD4+
Gated on CD8+
Media
PMA/Iono
TNFα
IFNγ
0.26
0.50
98.8
0.43
21.5
10.7
57.3
10.4
0.10
0.45
98.8
0.63
1.99
12.4
46.5
39.1
C
D
CD19
91.6
92.4
81.6
84.0
1.21
0
98.7
0.055
16.8
0.18
82.8
0.22

Supplementary Figure 3. Gating strategy for the intracellular cytokine staining (ICS) analysis. PBMCs derived from CLL patients and HDs were stimulated *in vitro*, with αCD3 and αCD28 mAbs or bacterial superantigens, SEB and TSST-1 (refer to Materials and Methods for details), compared to control unstimulated cultures (Nil). After 5 days, cultured cells were briefly re-stimulated with PMA/Ionomycin (PMA/Iono) compared to control media (Media) in the presence of Brefeldin A (4 hrs). **A-B)** Cells were surface-stained with fluorescently-labelled anti-CD3, anti-CD4 and anti-CD8 mAbs; fixed; permeabilised; and intracellularly stained with IFNγ and TNFα mAbs in ICS. As done before in Reus et al. 2021, left dot plots in A show the identification of viable lymphocytes (by FSC *vs* SSC). Thereafter, $CD3^+$ T cells were identified within gated viable lymphocytes (CD3 *vs* SSC; middle panels) and finally $CD4^+$ and $CD8^+$ T cells were identified within gated $CD3^+$ T cells (right plots), accounting for the known downregulation of CD4 expression after PMA/Iono exposure. Contour plots in B depict the downstream gating analysis of IFNγ and TNFα secretion in gated $CD4^+$ $CD3^+$ (left panels) and $CD8^+$ $CD3^+$ (right panels) T cells upon stimulation with PMA/Iono, compared to the respective media negative controls (Media). **C-D)** Cells were surface stained with anti-CD19 and anti-CD3 mAbs, fixed and permeabilized, before ICS with anti-IFNγ and anti-TNFα mAbs in ICS. Left dot plots in C show the identification of viable lymphocytes (by FSC *vs* SSC), while right contour plots show the gating of $CD19^+$ $CD3^-$ B, or CLL tumour, cells within viable gated lymphocytes for the PMA/Iono and the respective control media conditions. **D)** Contour plots depict the downstream gating analysis of IFNγ and TNFα production within gated $CD19^+$ $CD3^-$ viable B (or CLL tumour) cells in the PMA/Iono and the respective control media conditions. For both T and B cell analyses, net cytokine secreted amounts were calculated by

subtracting percentages detected in the control Media condition to the respective percentages in the PMA/Iono condition.

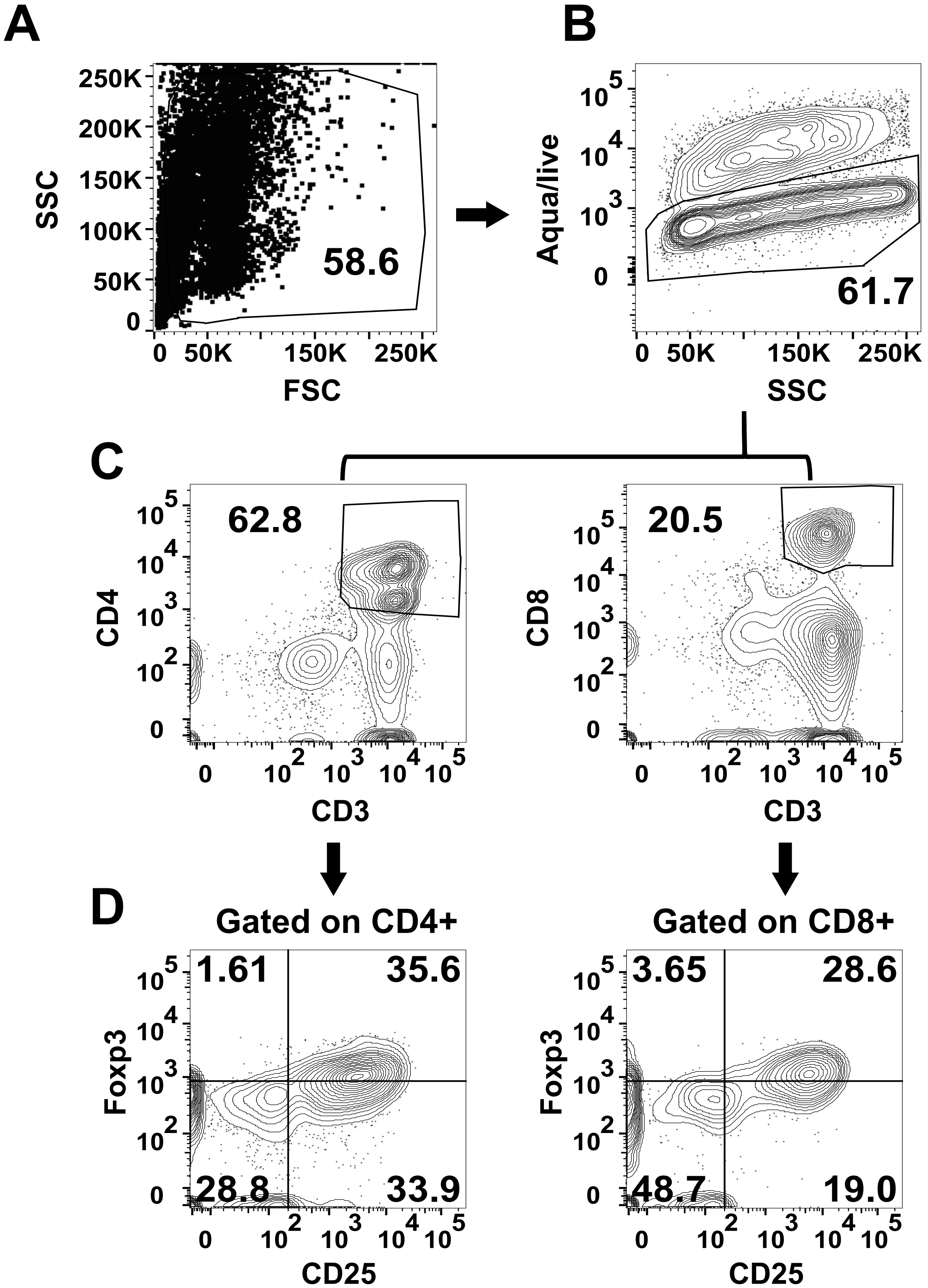
A
B
SSC
FSC
58.6
Aqua/live
SSC
61.7
C
CD4
CD3
62.8
CD8
CD3
20.5
D
Gated on CD4+
Foxp3
CD25
1.61
35.6
28.8
33.9
Gated on CD8+
Foxp3
CD25
3.65
28.6
48.7
19.0

Supplementary Figure 4. Gating strategy for the detection of Foxp3$^+$ CD25$^+$ regulatory T (Treg) cells. PBMCs from HD, HDY-42F-014 are shown as an example. Cells were stimulated as described in Supplementary Figure 3 and the Materials and Methods for five days. Thereafter, cells were surface-stained with anti-CD4; anti-CD8; anti-CD25 and anti-CD3 mAbs and the Live/Dead^{TM} Fixable Aqua fluorescent stain (thereafter referred to as Aqua/live stain), required in this protocol to identify viable cells given the known loss of FSC after permeabilization, and as done before (Taylor and Llewelyn, 2010). Cells were then fixed, permeabilized, and intracellularly stained with Foxp3 mAb. Samples were acquired on a BD LSR II Fortessa, using settings strictly defined and reproduced under a panel-specific rainbow profile. Data was analysed using FlowJo™ software. **A)** Dot plot illustrates the debris exclusion by defining a non-debris larger polygon gate in the FSC *vs* SSC plot. **B)** Viable lymphocytes within the non-debris gate are then identified by gating on cells that are negative for the Aqua/live stain *vs* SSC. **C)** CD4$^+$ CD3$^+$ (left contour plot) and CD8$^+$ CD3$^+$ (right contour plot) T cells are then identified within viable lymphocytes. **D)** Contour plots show the downstream gating analysis of Foxp3 against CD25 in gated CD4$^+$ CD3$^+$ (left) and CD8$^+$ CD3$^+$ (right) viable (Aqua/live$^-$) T cells.

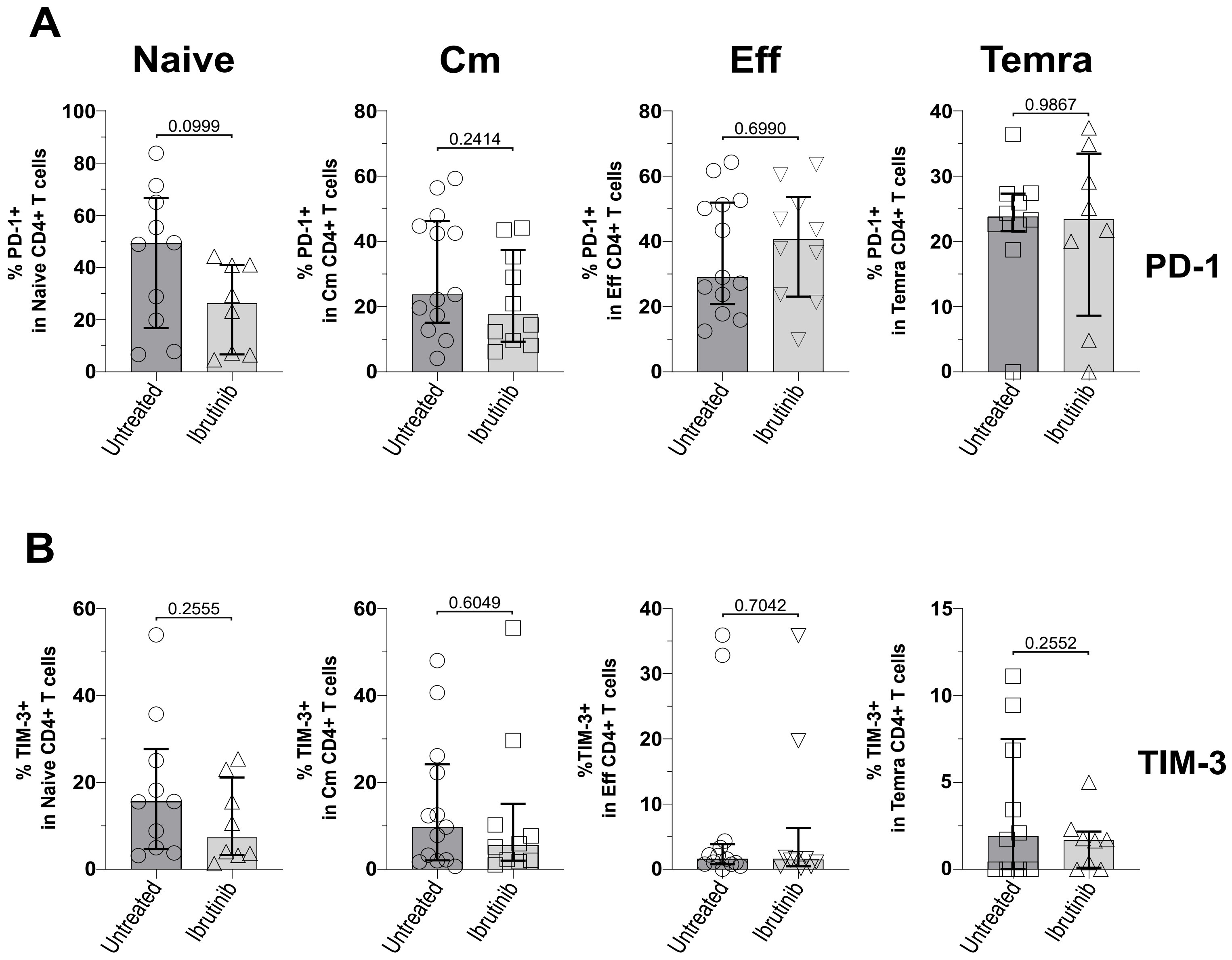
A
Naive
Cm
Eff
Temra
% PD-1+ in Naive CD4+ T cells
% PD-1+ in Cm CD4+ T cells
% PD-1+ in Eff CD4+ T cells
% PD-1+ in Temra CD4+ T cells
0.0999
0.2414
0.6990
0.9867
Untreated
Ibrutinib
PD-1
B
% TIM-3+ in Naive CD4+ T cells
% TIM-3+ in Cm CD4+ T cells
%TIM-3+ in Eff CD4+ T cells
% TIM-3+ in Temra CD4+ T cells
0.2555
0.6049
0.7042
0.2552
TIM-3

Supplementary Figure 5. CLL patients show similar levels of exhaustion-phenotype of $CD4^+$ T cells, *ex vivo*, irrespective of ibrutinib treatment. Expression of the exhaustion markers/checkpoint receptors (PD-1 and TIM-3) was investigated within Tn ($CCR7^+CD45RA^+$) and memory subsets identified by using CCR7 and CD45RA (Cm; $CCR7^+CD45RA^-$, Eff; $CCR7^-CD45RA^-$, and Temra; $CCR7^-CD45RA^+$), after gating on viable $CD3^+$ $CD4^+$ T cells, as shown in Supplementary Figure 1. **A-B)** Scatter plots with medians (bars) ± IQR show percentages of $PD1^+$ (A) and TIM-3^+ (B) in gated on Naïve, Cm, Eff and TEMRA $CD4^+$ T cell subsets (in order, from left to right), comparing levels of expression seen in ibrutinib-treated (n=8; light grey) and untreated (n=10; dark grey) CLL patients. Statistical significance was evaluated after assessing data normality (Shapiro-Wilk test), by using unpaired t or the Mann-Whitney U tests, respectively for parametric or non-parametric data.

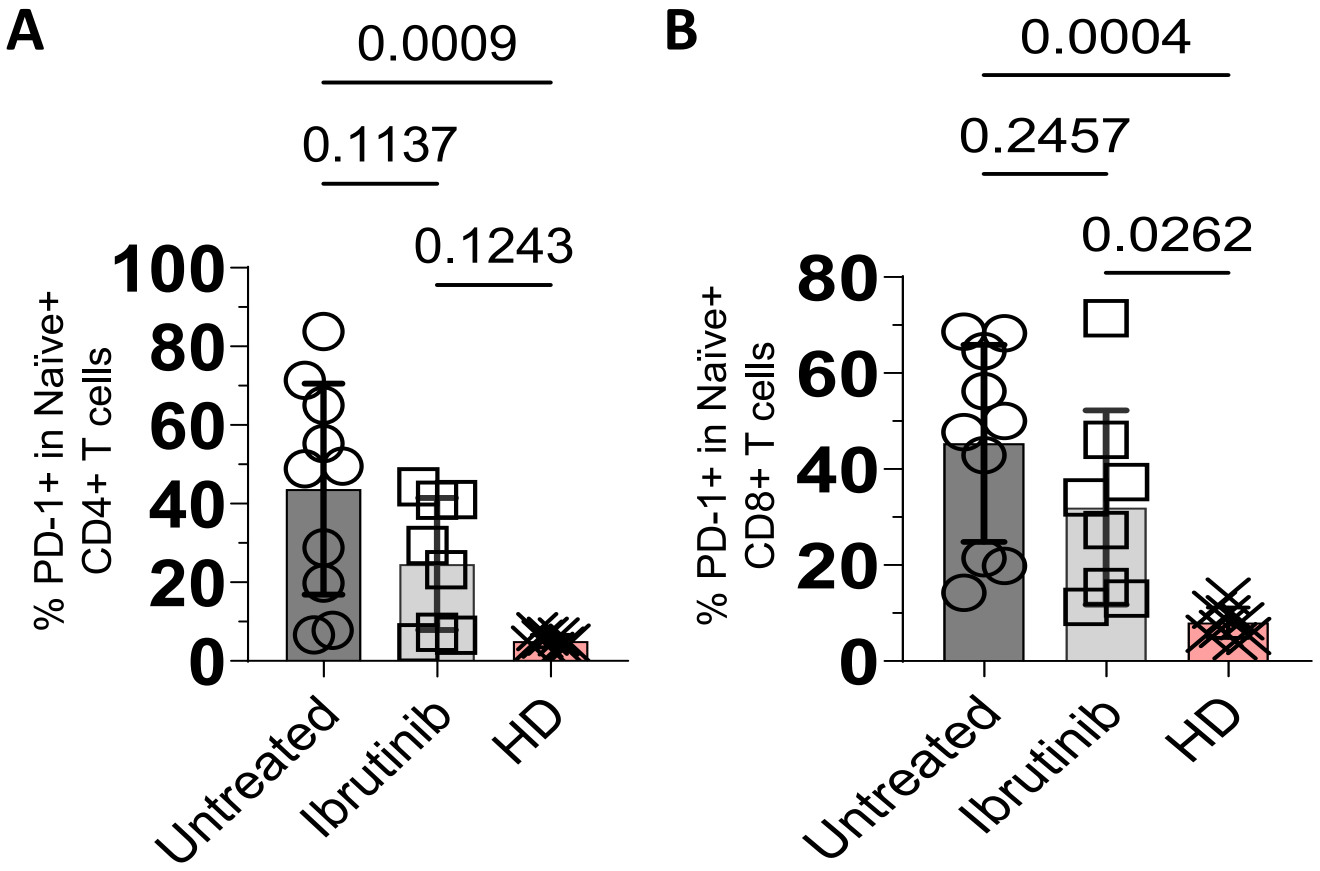
A
0.0009
0.1137
0.1243
100
80
60
40
20
0
% PD-1+ in Naïve+ CD4+ T cells
Untreated
Ibrutinib
HD
B
0.0004
0.2457
0.0262
80
60
40
20
0
% PD-1+ in Naïve+ CD8+ T cells
Untreated
Ibrutinib
HD

Supplementary Figure 6. Naïve-like T cells from CLL patients express significantly higher PD-1 levels than HD-derived counterparts. Fresh PBMCs were immunophenotyped *ex vivo*, comparing ibrutinib-treated (n=8; light grey) and untreated (n=10; dark grey) CLL patients to HDs (n=8; light red). Data normality was assessed with the Shapiro-Wilk test before evaluating significance with one-way ANOVA or the Kruskal-Wallis test with Tukey or Dunn's multiple comparison correction tests for parametric and nonparametric data respectively. **A-B)** Scatter plots with bars show percentages of PD-1$^+$ within the naïve-like (CCR7$^+$CD45RA$^+$) subsets, after gating on viable CD3$^+$ CD4$^+$ (A) and CD3$^+$ CD8$^+$ (B) T cells. *P ≤ .05 and ***P ≤ .0005 were considered significant.

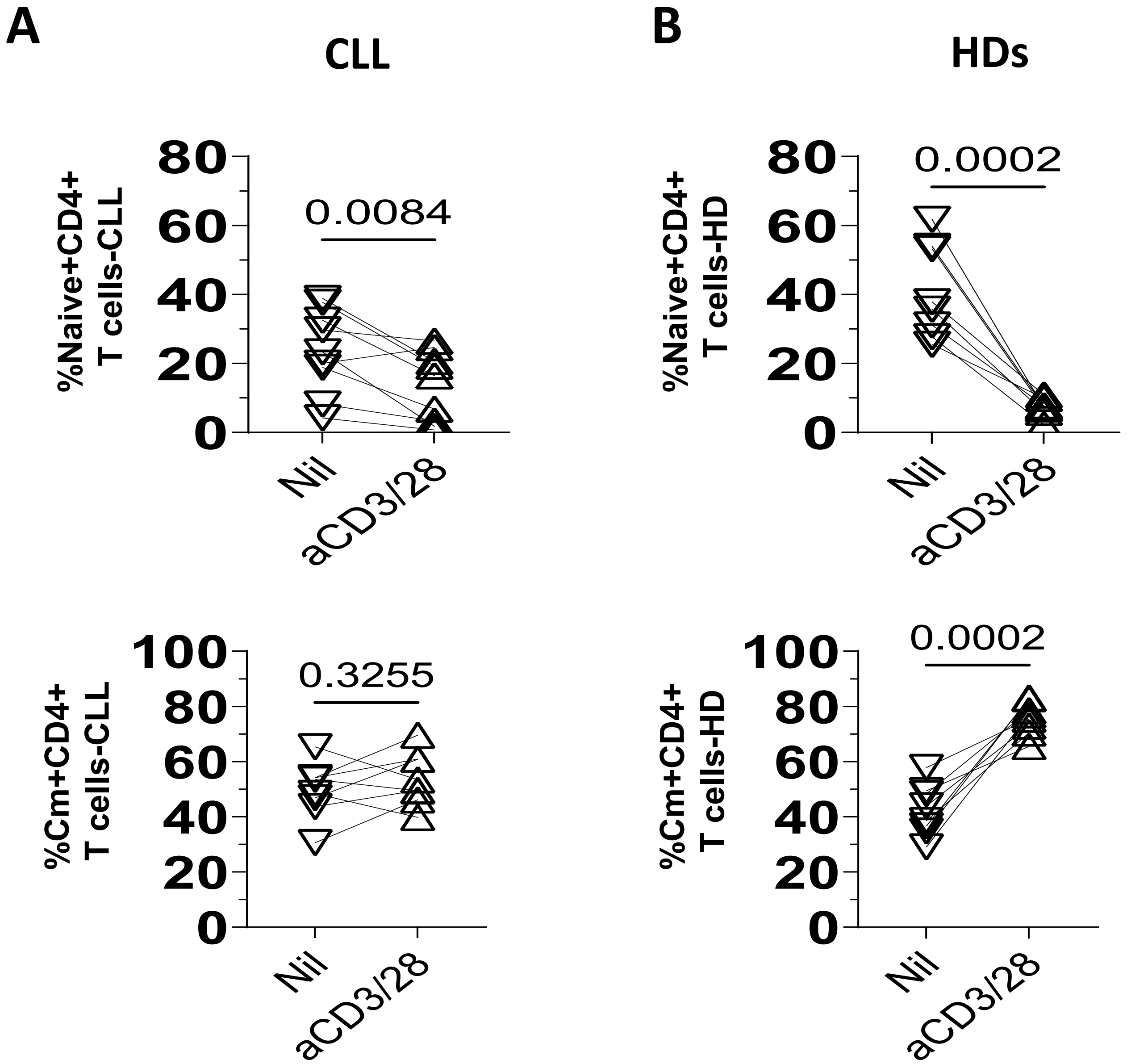
A
CLL
B
HDs
0.0084
%Naive+CD4+ T cells-CLL
0.0002
%Naive+CD4+ T cells-HD
0.3255
%Cm+CD4+ T cells-CLL
0.0002
%Cm+CD4+ T cells-HD
Nil
aCD3/28

Supplementary Figure 7. The archetypal TCR-stimulation, aCD3/28 triggers stronger activation of Cm responses in cultures derived from HDs more than CLL patients, while reducing naive-like precursors. A-B) Plots show the percentage of naïve-like (top) and Cm (bottom) $CD4^+$ T cells in cultures derived from CLL patients (A) and HDs (B) after αCD3/28 stimulation (upward triangles) compared to unstimulated controls (Nil, downward triangles). Statistical significance was determined using paired t tests, after assessing the data normality with Shapiro-Wilk tests. **, $P \leq .005$ and ***, $P \leq .0005$ were considered statistically significant.

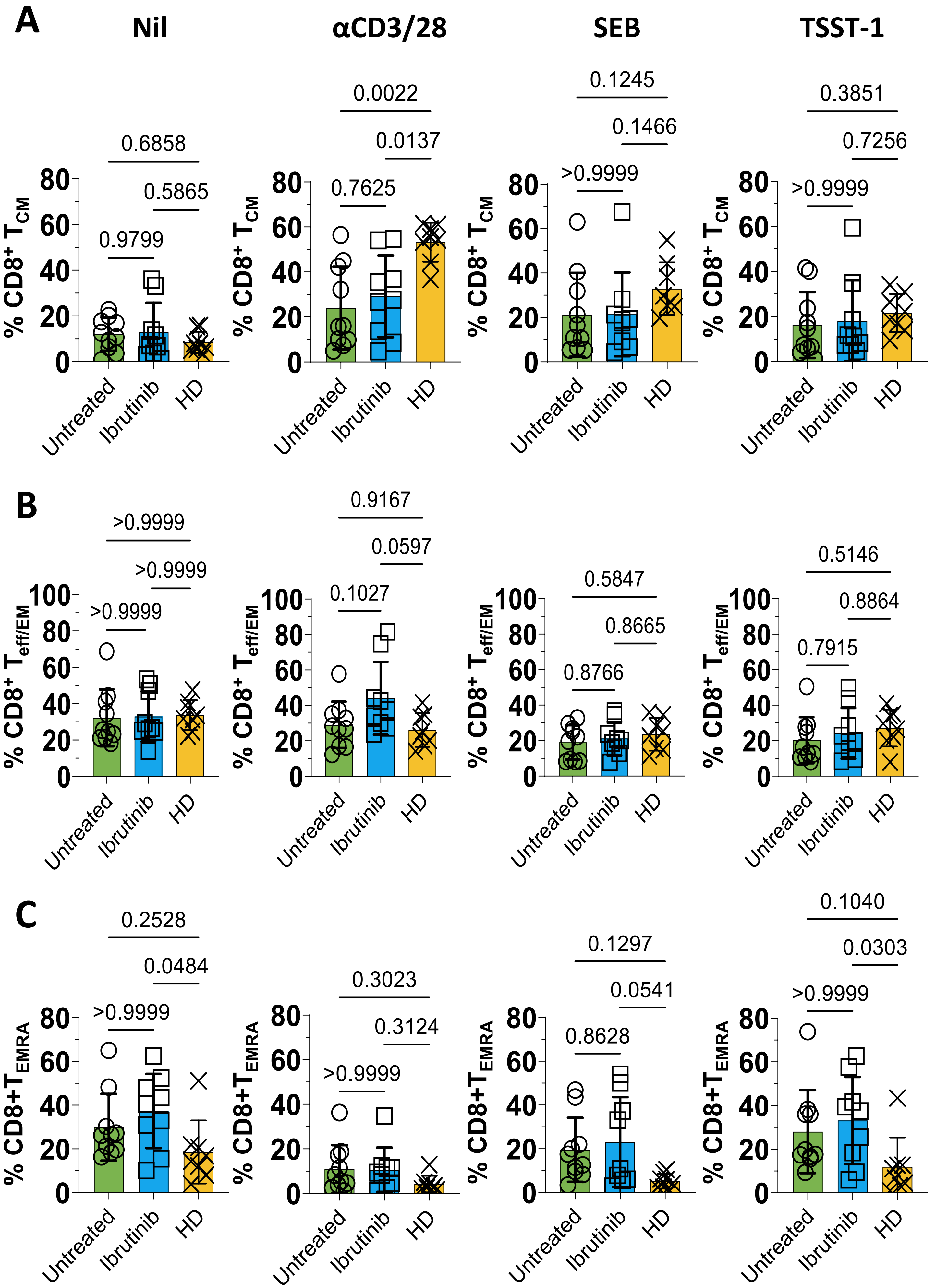

A
Nil
αCD3/28
SEB
TSST-1
% CD8+ TCM
0.6858
0.5865
0.9799
0.0022
0.0137
0.7625
0.1245
0.1466
>0.9999
0.3851
0.7256
>0.9999
Untreated
Ibrutinib
HD
B
% CD8+ Teff/EM
>0.9999
>0.9999
>0.9999
0.9167
0.0597
0.1027
0.5847
0.8665
0.8766
0.5146
0.8864
0.7915
C
% CD8+TEMRA
0.2528
0.0484
>0.9999
0.3023
0.3124
>0.9999
0.1297
0.0541
0.8628
0.1040
0.0303
>0.9999

Supplementary Figure 8. CD8^{+} T cell memory subset profiles in untreated and ibrutinib-treated CLL patients compared to HDs, following stimulations with αCD3/28 and SA SAgs. Fresh PBMCs from ibrutinib-treated (n=9, blue bars) and untreated (n=10, green bars) CLL patients or HDs (n=8, yellow bars) were stimulated *in vitro* for 5 days with αCD3/28 mAbs or staphylococcal superantigens, SEB and TSST-1 (refer to Materials and Methods for details), compared to unstimulated control cultures (Nil). Thereafter, cells were harvested, and surface stained, with anti-CD3, anti-CD8, anti-CD45RA, and anti-CCR7 mAbs. **A-C)** Scatter plots with bars show the percentages of Cm (A; CCR7^{+}CD45RA^{-}), Eff/Em (B; CCR7^{-}CD45RA^{-}) and Temra (C; CCR7^{-}CD45RA^{+}) in gated CD8^{+} T cells in the three experimental groups described above, for each of the culture conditions specified at the top. After assessment of data normality with Shapiro-Wilk tests, statistical significance was determined using the one-way ANOVA or the Kruskal-Wallis tests with Tukey or Dunn's multiple comparison correction tests for parametric and nonparametric data respectively. *P ≤ .05 and **p≤0.005 were considered significant. As for CD4+ T cells (see Figure 2B), significantly higher accumulation of Cm CD8+ T cells was detected in aCD3/28 cultures derived from HDs rather than CLL patients. In contrast, overall, TEff/Em and Temra CD8+ T cell responses were not significantly impacted in the same cultures.

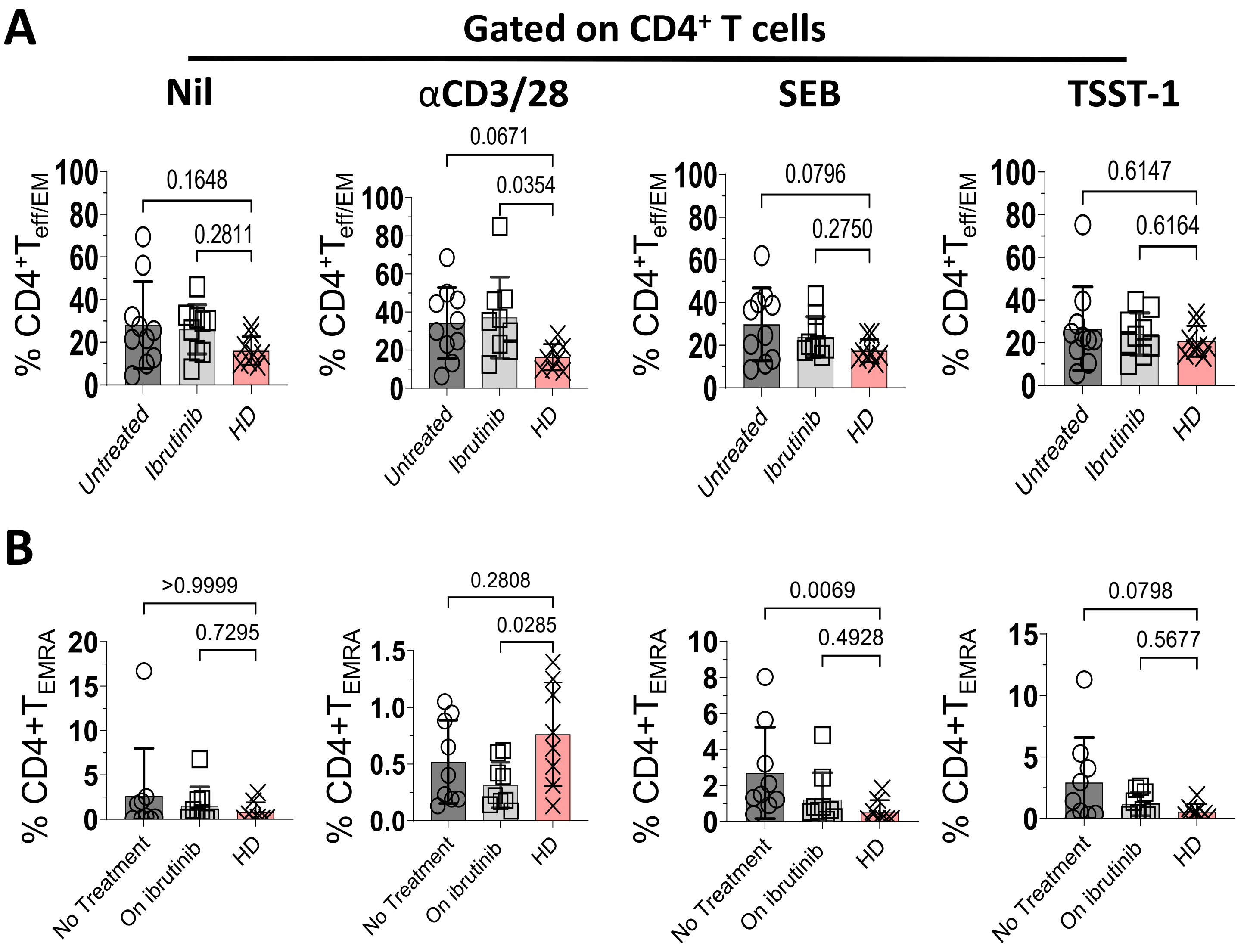

A
Gated on CD4+ T cells
Nil
αCD3/28
SEB
TSST-1
% CD4+Teff/EM
0.1648
0.2811
0.0671
0.0354
0.0796
0.2750
0.6147
0.6164
Untreated
Ibrutinib
HD
B
% CD4+TEMRA
>0.9999
0.7295
0.2808
0.0285
0.0069
0.4928
0.0798
0.5677
No Treatment
On ibrutinib
HD

Supplementary Figure 9. CD4+ TEff/Em and Temra subset profiles in untreated and ibrutinib-treated CLL patients compared to HDs, following stimulations with αCD3/28 and SA SAgs. Fresh PBMCs from ibrutinib-treated (n=9, light grey) and untreated (n=10, dark grey) CLL patients or HDs (n=8, light red) were stimulated *in vitro* for 5 days with αCD3/28 stimulatory mAbs or staphylococcal superantigens, SEB and TSST-1 (refer to Materials and Methods for details), compared to unstimulated control cultures (Nil). Thereafter, cells were harvested, and surface stained, with anti-CD3, anti-CD4, anti-CD45RA, and anti-CCR7 mAbs. **A-B**) Scatter plots with bars show the percentages of Eff/Em (A; $CCR7^{-}CD45RA^{-}$) and Temra (B; $CCR7^{-}CD45RA^{+}$) in gated $CD4^{+}$ T cells in the three experimental groups described above, for each of the culture conditions specified at the top. After assessment of data normality with Shapiro-Wilk tests, statistical significance was determined by using the one-way ANOVA or the Kruskal-Wallis tests with Tukey or Dunn's multiple comparison correction tests for parametric and nonparametric data respectively. *P ≤ .05 and **p≤0.005 were considered significant. Unlike the case of Cm CD4+ T cells (see Figure 2B), TEff/Em and Terma weakly changed in the same cultures representing relatively minor fractions by day 5.

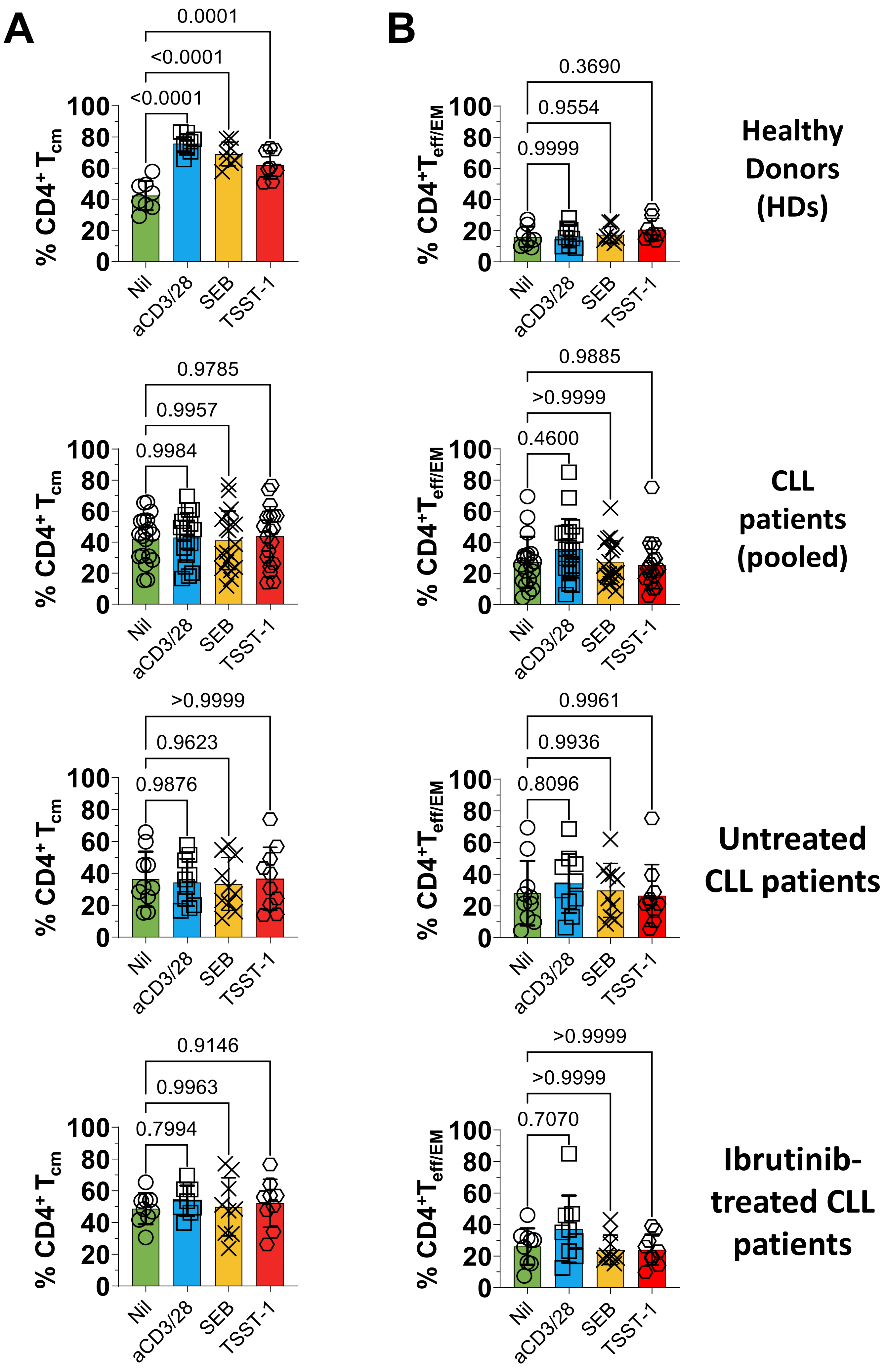
A
B
0.0001
<0.0001
<0.0001
0.3690
0.9554
0.9999
% CD4+ Tcm
% CD4+Teff/EM
Nil
aCD3/28
SEB
TSST-1
Healthy Donors (HDs)
0.9785
0.9957
0.9984
0.9885
>0.9999
0.4600
CLL patients (pooled)
>0.9999
0.9623
0.9876
0.9961
0.9936
0.8096
Untreated CLL patients
0.9146
0.9963
0.7994
>0.9999
>0.9999
0.7070
Ibrutinib-treated CLL patients

Supplementary Figure 10. Unlike CLL counterparts, HD-derived cultures show significant accumulation of Cm, but not Eff/Em CD4$^+$ T cells upon αCD3/28, SEB and TSST-1 stimulations. Fresh PBMCs were cultured for 5 days and then immunophenotyped as described in Figure 2 and Materials and Methods. **A-B)** Scatter plots with bars show the percentages of Cm (A) and Eff/Em (B) in gated CD4$^+$ T cells from HDs (n=8, top row), pooled CLL groups (n=19, second row), untreated (n=10, third row), and ibrutinib-treated (n=9, bottom row) CLL patients after αCD3/28 (blue bars), SEB (yellow bars), TSST-1 (red bars), compared to unstimulated controls (Nil) cultures (green bars). After evaluating data normality with Shapiro-Wilk tests, statistical significance was determined by using the one-way ANOVA or the Kruskal-Wallis tests with Tukey or Dunn's multiple comparison correction tests for parametric and nonparametric data respectively. ***$p \leq 0.0005$ was considered significant.

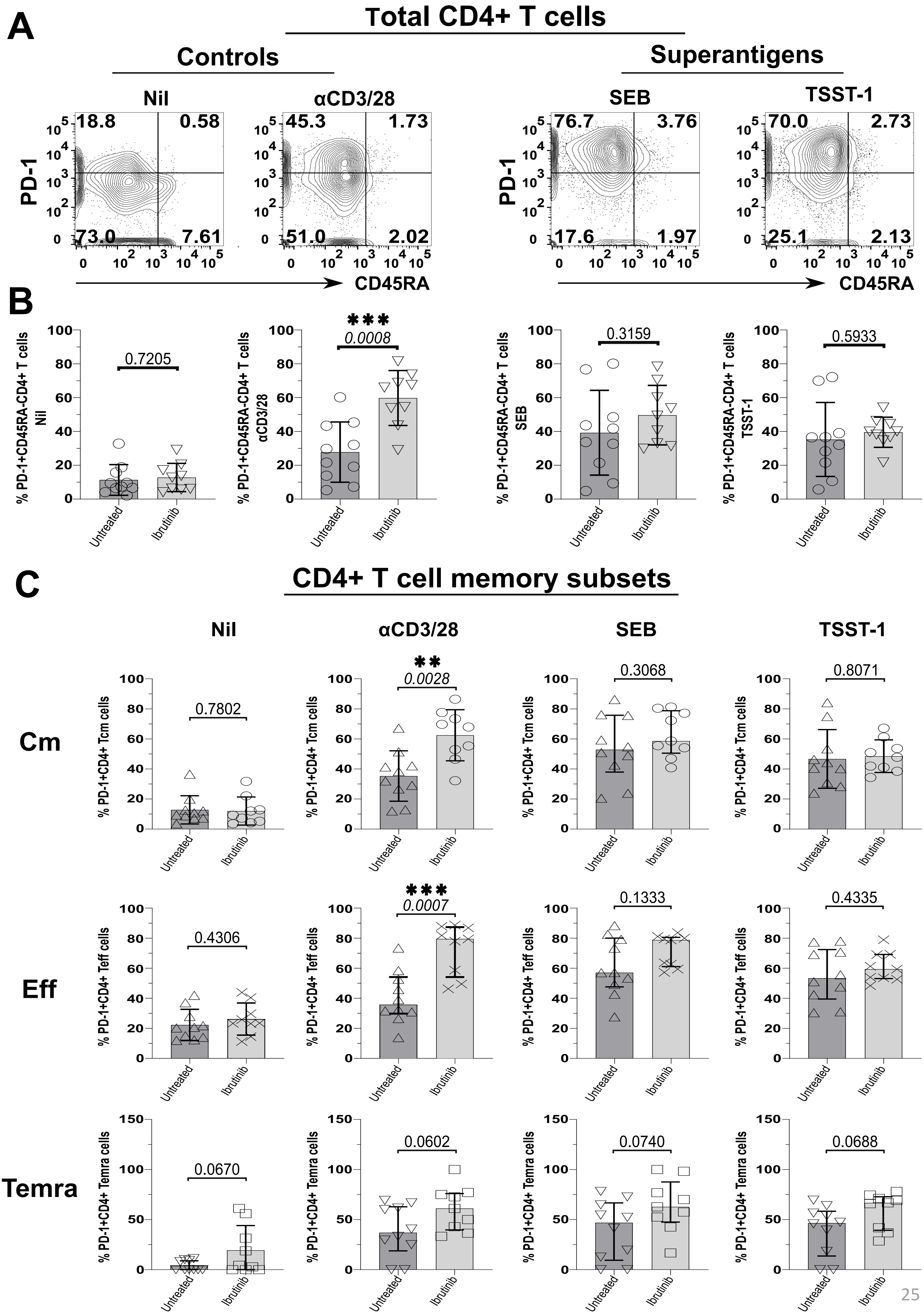

A
Total CD4+ T cells
Controls
Superantigens
Nil
αCD3/28
SEB
TSST-1
PD-1
CD45RA
18.8
0.58
73.0
7.61
45.3
1.73
51.0
2.02
76.7
3.76
17.6
1.97
70.0
2.73
25.1
2.13
B
% PD-1+CD45RA-CD4+ T cells Nil
% PD-1+CD45RA-CD4+ T cells αCD3/28
% PD-1+CD45RA-CD4+ T cells SEB
% PD-1+CD45RA-CD4+ T cells TSST-1
0.7205
***
0.0008
0.3159
0.5933
Untreated
Ibrutinib
C
CD4+ T cell memory subsets
Cm
Eff
Temra
% PD-1+CD4+ Tcm cells
% PD-1+CD4+ Teff cells
% PD-1+CD4+ Temra cells
0.7802
**
0.0028
0.3068
0.8071
0.4306
***
0.0007
0.1333
0.4335
0.0670
0.0602
0.0740
0.0688

Supplementary Figure 11. Central and effector memory CD4$^+$ T cells show elevated PD-1 expression upon αCD3/28 stimulation, preferentially in cultures derived from ibrutinib-treated CLL patients. PBMCs of ibrutinib-treated and untreated CLL patients were stimulated *in vitro*, as described in Supplementary Figure 3 and the Materials and Methods for five days. Thereafter, cells were harvested, and surface-stained with anti-CD3, anti-CD4, anti-CD45RA, anti-CCR7 and anti-PD-1 mAbs. Viable CD4$^+$ T cells were identified as shown in Supplementary Figure 1. **A)** Example contour plots from an untreated CLL patient (P635) show surface expression of CD45RA (x-axis) and PD-1 (y-axis) within viable CD4$^+$ T cells after 5d-stimulation with superantigens, SEB, and TSST-1 (left) compared to control conditions (right panels): αCD3/28 and unstimulated (Nil) cultures. **B)** Scatter plots with bars show the median percentages ± IQR of PD-1$^+$ cells within total memory CD45RA$^-$ CD4$^+$ T cells detected in the above cultures derived from either ibrutinib-treated (n=9) or untreated (n=10) CLL patients. **C)** Scatter plots with bars depict median percentages ± IQR of PD-1$^+$ cells within Cm (top row), Eff (middle row) and Temra (bottom row) CD4$^+$ T cell subsets in the same cultures comparing ibrutinib-treated (n=9) and untreated (n=10) CLL patients. Statistical significance was determined after evaluating data normality with Shapiro-Wilk tests, by using the unpaired t or the Mann-Whitney U tests, respectively for parametric or non-parametric data. **P≤0.005 and ***P≤0.0005.

.

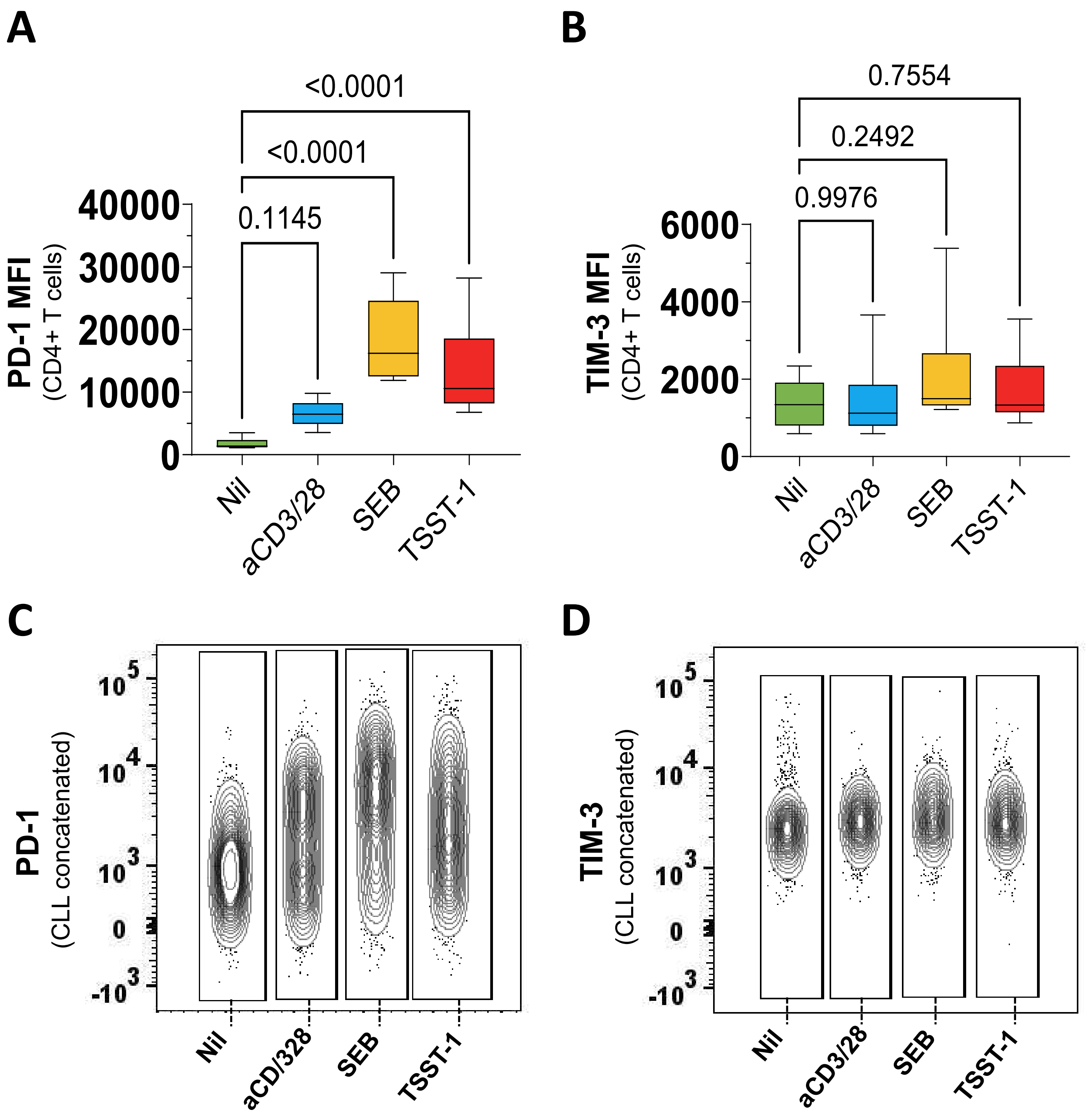

Sample	Mean	SD
**Nil**	1365	138
**αCD3/28**	3480	130
**SEB**	7824	136
**TSST-1**	5502	192

Sample	Mean	SD
**Nil**	3145	147
**αCD3/28**	3136	50.6
**SEB**	3726	69.3
**TSST-1**	3317	72

Supplementary Figure 12. SEB and TSST-1 more than αCD3/28 stimulation promote higher levels of PD-1 and TIM-3 expression in CD4$^+$ T cells when compared to unstimulated controls. PBMCs of CLL patients were stimulated *in vitro*, as described in Supplementary Figure 3 and the Materials and Methods for five days. Thereafter, cells were harvested, and surface-stained with anti-CD3, anti-CD4, anti-CD45RA, anti-CCR7, anti-PD-1 and anti-TIM-3 mAbs. Viable CD4+ T cells were identified as shown in Supplementary Figure 1. After acquisition, for each experimental culture condition, 7200 events from individual CLL patient samples (n=17) were concatenated using the FlowJO^{TM} version 10. Thereafter, a concatenated file of the four concatenated culture conditions was created and further analysed in comparison to statistically pooled data. **A-B**) Box plots show the MFI of PD-1 (A) and TIM-3 (B) expression in total CD4$^+$ CD3$^+$ T cells in statistically pooled analyses (median ± min & max range) of cultures with αCD3/28 (blue bars), SEB (yellow bars) and TSST-1 (red bars) relative to Nil cultures (green bars) derived from CLL patients. Statistical significance was determined after evaluating the data normality with Shapiro-Wilk test using the one-way ANOVA test or the Kruskal-Wallis test with Tukey or Dunn's multiple comparison correction tests for parametric and nonparametric data respectively. ***, P ≤ .0005 was considered statistically significant. **C-D**) Concatenation flow cytometry plots show the concatenated MFIs of PD-1 (C) and TIM-3 (D) in total CD4$^+$ CD3$^+$ T cells, for each culture condition tested: αCD3/28, SEB and TSST-1 stimulation, compared to unstimulated control cultures (Nil). The colour-coded tables show the mean and SD of each concatenated parameter for the stimulations mentioned above. Violet shades indicate low to high levels of expression, respectively from light to dark tones. Overall, concatenated analyses agreed with statistically pooled data analyses.

A

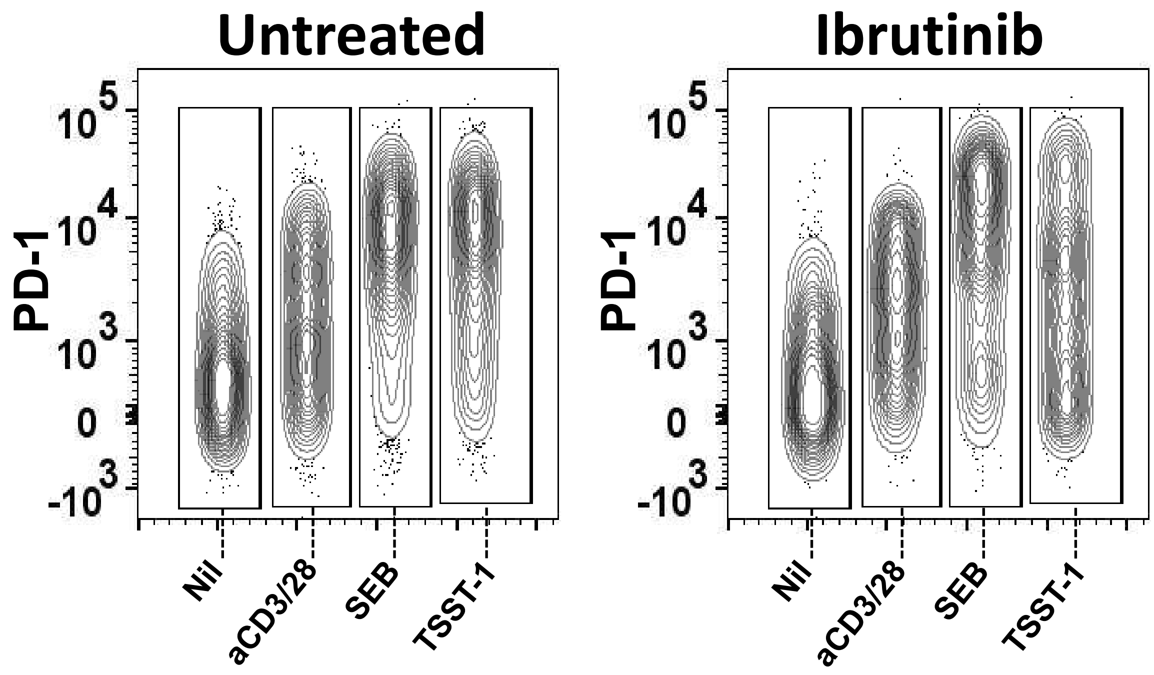

Sample	Mean	SD
**Nil**	614	189
**αCD3/28**	2361	170
**SEB**	8409	183
**TSST-1**	4833	161

Sample	Mean	SD
**Nil**	345	239
**αCD3/28**	3541	116
**SEB**	5858	124
**TSST-1**	2859	184

B

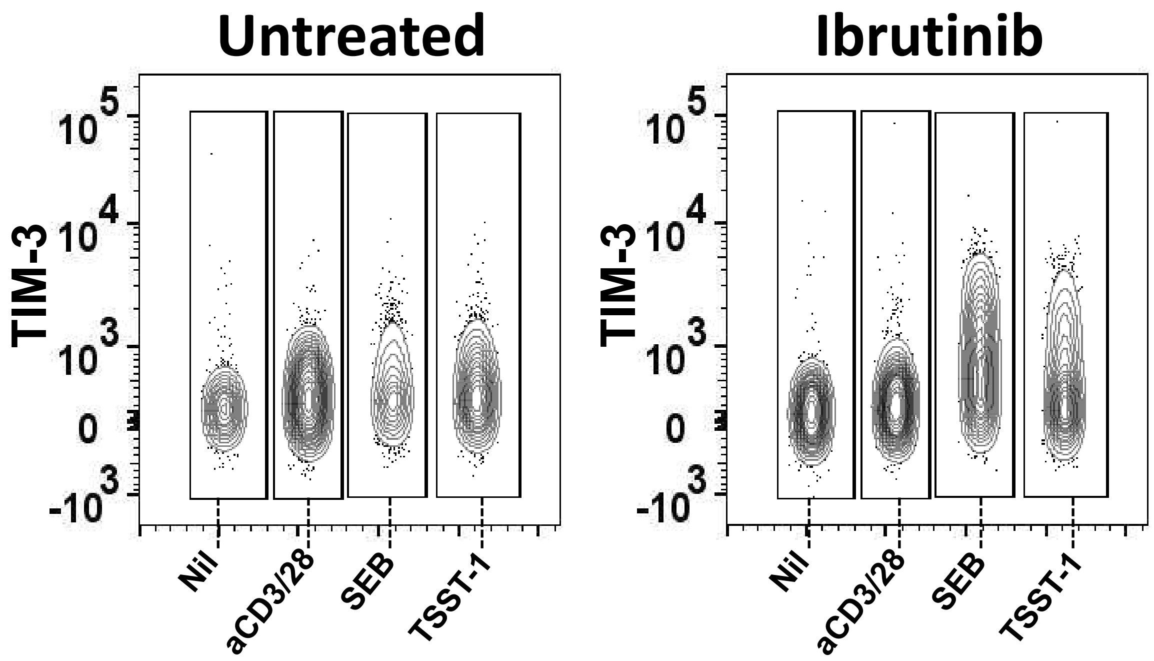

Sample	Mean	SD
**Nil**	245	707
**αCD3/28**	340	156
**SEB**	504	166
**TSST-1**	423	127

Sample	Mean	SD
**Nil**	162	574
**αCD3/28**	278	411
**SEB**	1105	116
**TSST-1**	738	228

Supplementary Figure 13. Concatenated MFI analyses reveal higher TIM-3 expression on CD4$^+$ T cells from ibrutinib-treated rather than untreated CLL patients, upon SEB and TSST-1 stimulations. PBMCs of CLL patients were stimulated and then immunophenotyped as described in Supplementary Figure 12. After acquisition, for each experimental culture condition, 8000 events from individual untreated (n=8) and ibrutinib-treated (n=9) CLL patient samples were concatenated using the FlowJO^{TM} version 10. Thereafter, for each patient sub-cohort, concatenated files of the four concatenated culture conditions were created and further analysed. **A-B**) Concatenation flow cytometry plots of untreated (left panels) and ibrutinib-treated (right panels) CLL patients show the concatenated MFIs of PD-1 (A) and TIM-3 (B) in total CD4$^+$ CD3$^+$ T cells, for each culture condition tested: αCD3/28 (blue), SEB (yellow) and TSST-1 (red) stimulation, compared to unstimulated control cultures (Nil; green). The colour-coded tables show the mean and SD of each concatenated parameter for the stimulations mentioned above. Violet shades indicate low to high levels of expression, respectively from light to dark tones.

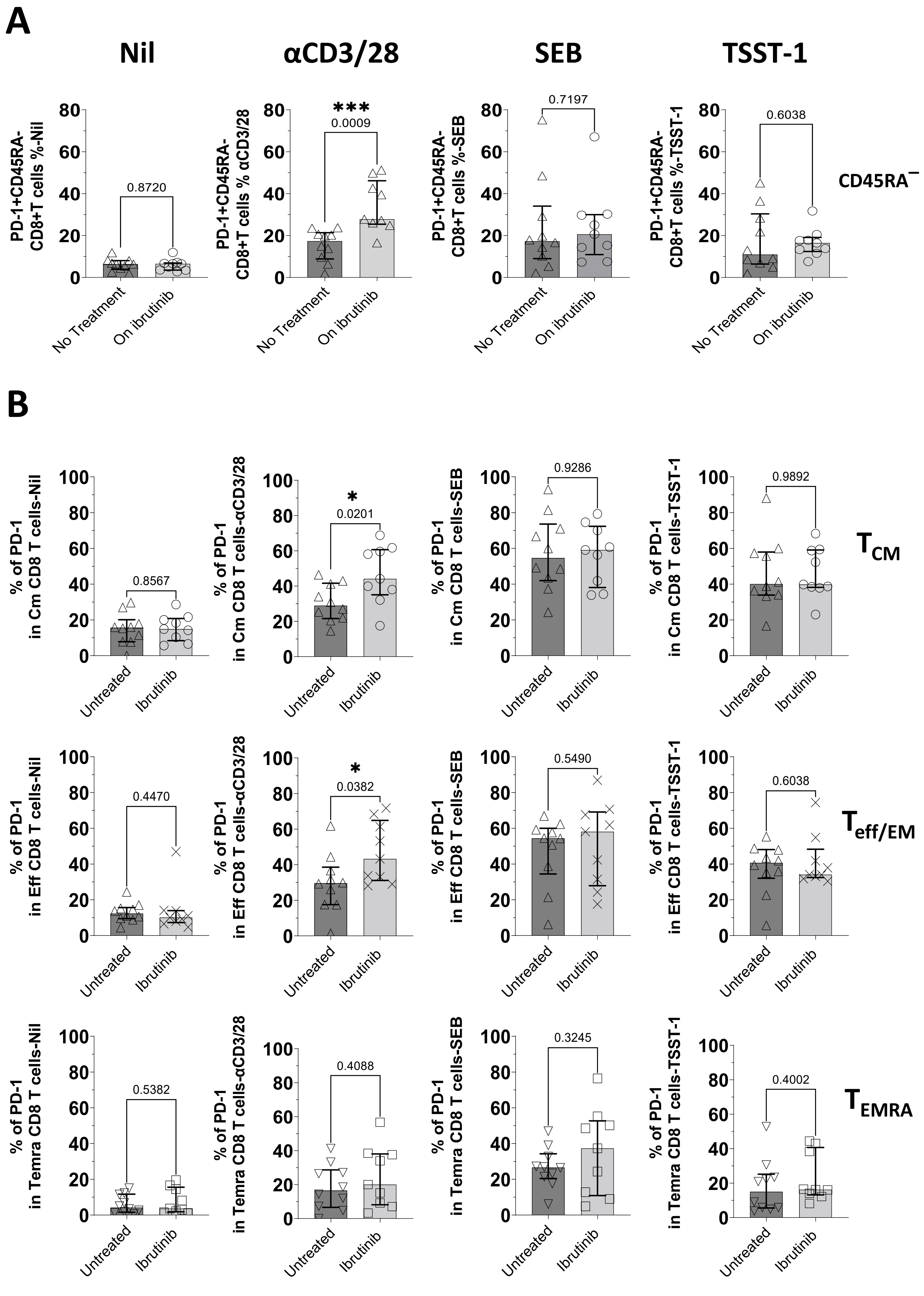
A
Nil
αCD3/28
SEB
TSST-1
PD-1+CD45RA- CD8+T cells %-Nil
PD-1+CD45RA- CD8+T cells % αCD3/28
PD-1+CD45RA- CD8+T cells %-SEB
PD-1+CD45RA- CD8+T cells %-TSST-1
0.8720
***
0.0009
0.7197
0.6038
CD45RA⁻
No Treatment
On ibrutinib
B
% of PD-1 in Cm CD8 T cells-Nil
% of PD-1 in Cm CD8 T cells-αCD3/28
% of PD-1 in Cm CD8 T cells-SEB
% of PD-1 in Cm CD8 T cells-TSST-1
0.8567
*
0.0201
0.9286
0.9892
T CM
% of PD-1 in Eff CD8 T cells-Nil
% of PD-1 in Eff CD8 T cells-αCD3/28
% of PD-1 in Eff CD8 T cells-SEB
% of PD-1 in Eff CD8 T cells-TSST-1
0.4470
*
0.0382
0.5490
0.6038
T eff/EM
% of PD-1 in Temra CD8 T cells-Nil
% of PD-1 in Temra CD8 T cells-αCD3/28
% of PD-1 in Temra CD8 T cells-SEB
% of PD-1 in Temra CD8 T cells-TSST-1
0.5382
0.4088
0.3245
0.4002
T EMRA
Untreated
Ibrutinib

Supplementary Figure 14. Central and effector memory CD8$^+$ T cells show increased PD-1 expression upon αCD3/28 stimulation, preferentially in cultures derived from ibrutinib-treated CLL patients. PBMCs of ibrutinib-treated and untreated CLL patients were stimulated *in vitro*, as described in Supplementary Figure 3 and the Materials and Methods for five days. Thereafter, cells were harvested, and surface stained, with anti-CD3, anti-CD8, anti-CD45RA, anti-CCR7 and anti-PD-1 mAbs. Viable CD8$^+$ T cells were identified as shown in Supplementary Figure 1. **A)** Scatter plots with bars show the median percentages ± IQR of PD-1$^+$ cells within total memory CD45RA$^-$ CD8$^+$ T cells detected in the above cultures derived from either ibrutinib-treated (n=9, light grey) or untreated (n=10, dark grey) CLL patients. **B)** Scatter plots with bars depict median percentages ± IQR of PD-1$^+$ cells within Cm (top row), Eff/Em (middle row) and Temra (bottom row) CD8$^+$ T cell subsets in the same cultures comparing ibrutinib-treated (n=9, light grey) and untreated (n=10, dark grey) CLL patients. Statistical significance was determined after evaluating data normality with Shapiro-Wilk tests, by using the unpaired t or the Mann-Whitney U tests, respectively for parametric or non-parametric data. *P≤0.05 and ***P≤0.0005.

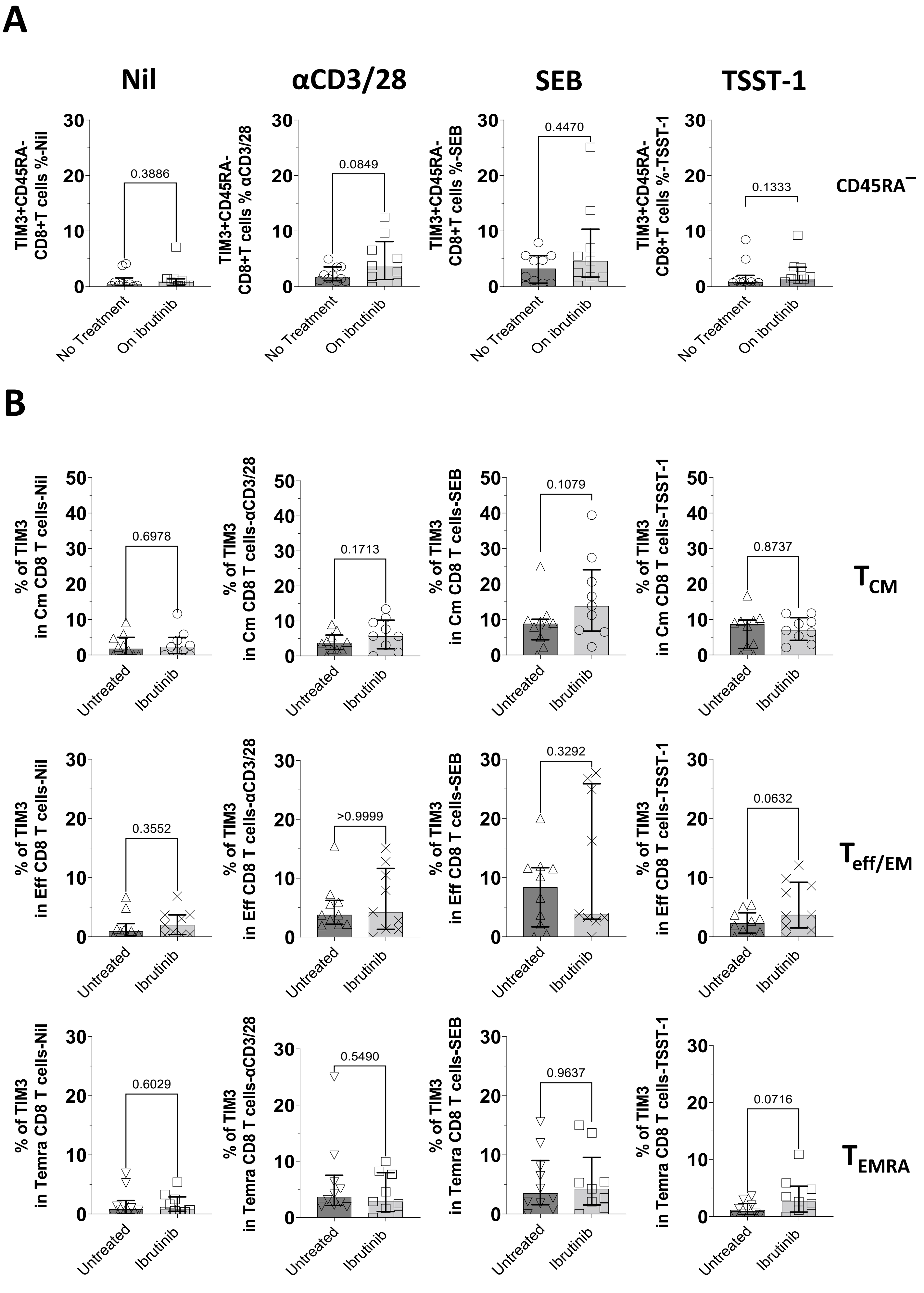
A
Nil
αCD3/28
SEB
TSST-1
TIM3+CD45RA-CD8+T cells %-Nil
TIM3+CD45RA-CD8+T cells % αCD3/28
TIM3+CD45RA-CD8+T cells %-SEB
TIM3+CD45RA-CD8+T cells %-TSST-1
0.3886
0.0849
0.4470
0.1333
No Treatment
On ibrutinib
CD45RA⁻
B
% of TIM3 in Cm CD8 T cells-Nil
% of TIM3 in Cm CD8 T cells-αCD3/28
% of TIM3 in Cm CD8 T cells-SEB
% of TIM3 in Cm CD8 T cells-TSST-1
0.6978
0.1713
0.1079
0.8737
TCM
% of TIM3 in Eff CD8 T cells-Nil
% of TIM3 in Eff CD8 T cells-αCD3/28
% of TIM3 in Eff CD8 T cells-SEB
% of TIM3 in Eff CD8 T cells-TSST-1
0.3552
>0.9999
0.3292
0.0632
Teff/EM
% of TIM3 in Temra CD8 T cells-Nil
% of TIM3 in Temra CD8 T cells-αCD3/28
% of TIM3 in Temra CD8 T cells-SEB
% of TIM3 in Temra CD8 T cells-TSST-1
0.6029
0.5490
0.9637
0.0716
TEMRA
Untreated
Ibrutinib

Supplementary Figure 15. Ibrutinib-treated and untreated CLL patients show similar TIM-3 percentage expression in CD8$^+$ T cell memory subsets after αCD3/28 and SA SAgs stimulations. PBMCs of ibrutinib-treated and untreated CLL patients were stimulated *in vitro*, as described in Supplementary Figure 3. At day 5 of culture, cells were harvested and surface stained with anti-CD3, anti-CD8, anti-CD45RA, anti-CCR7 and anti-TIM-3, as indicated in the Materials and Methods section. **A)** Scatter plots with bars show the percentage of TIM-3$^+$ within total memory CD45RA$^-$ CD8$^+$ T cells detected after 5d-stimulation with αCD3/28, SEB, and TSST-1 compared to unstimulated control cultures (Nil) from either ibrutinib-treated (n=9, light grey) or untreated (n=10, dark grey) CLL patients. **B**) Scatter plots with bars demonstrate the percentage of TIM-3$^+$ cells within Cm (top row), Eff/Em (middle row) and Temra (bottom row) CD8$^+$ T cell subsets in the same cultures comparing ibrutinib-treated (n=9, light grey) and untreated (n=10, dark grey) CLL patients. Statistical significance was determined after evaluating the data normality with Shapiro-Wilk test using the unpaired T test or the Mann-Whitney test for parametric or non-parametric data respectively.

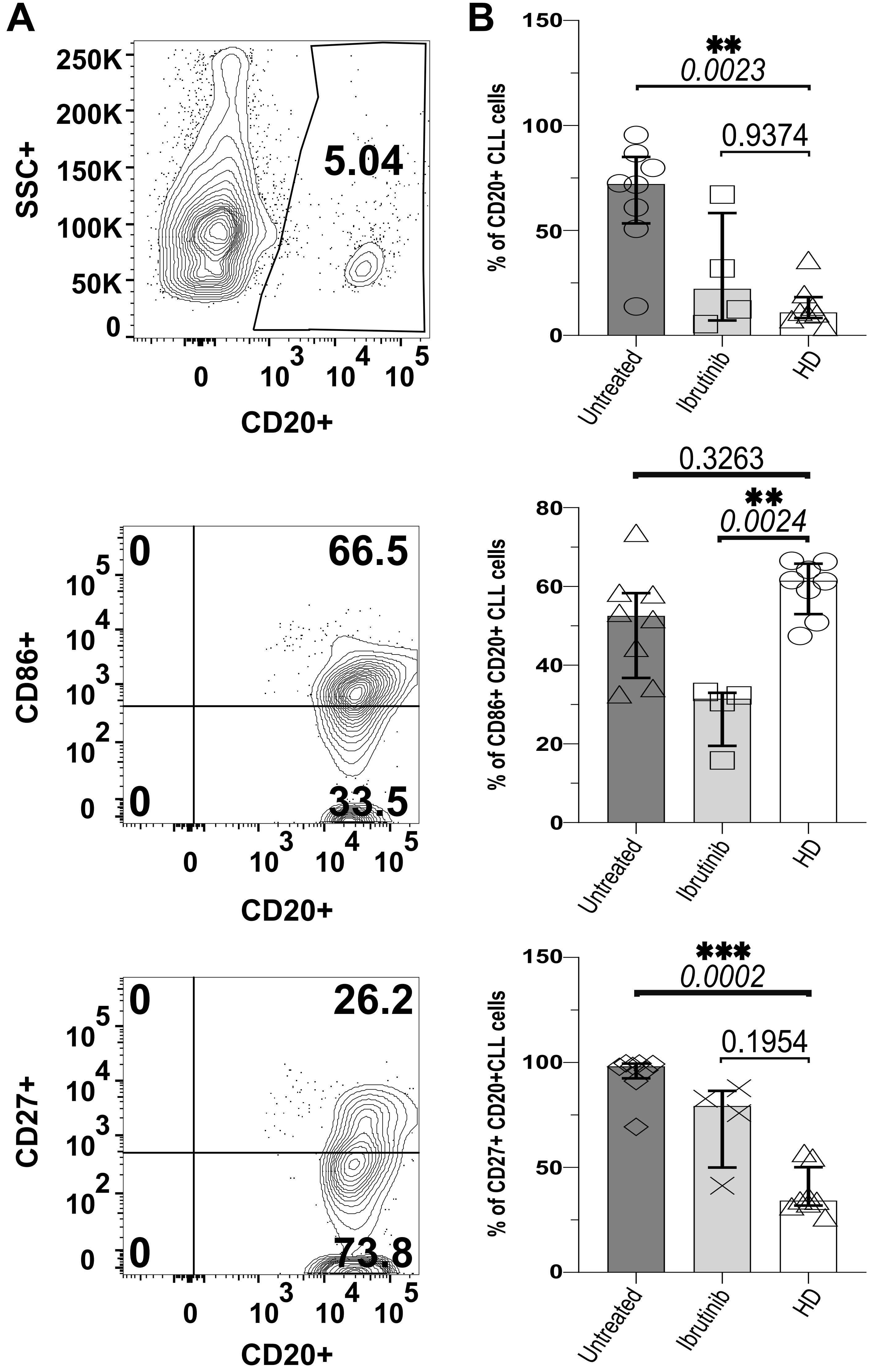
A
B
SSC+
250K
200K
150K
100K
50K
0
5.04
CD20+
% of CD20+ CLL cells
**
0.0023
0.9374
Untreated
Ibrutinib
HD
CD86+
66.5
33.5
0.3263
0.0024
% of CD86+ CD20+ CLL cells
CD27+
26.2
73.8
***
0.0002
0.1954
% of CD27+ CD20+CLL cells

Supplementary Figure 16. Expression of CD86 and CD27 in CD20$^+$ B cells comparing CLL patient sub-cohorts to HDs. A) Example contour plots from HDY-74M-017 show: (i) gated CLL tumour cells, in the CD20$^+$ *vs* SSC top contour plot); (ii) expression of B cell co-stimulation marker, CD86 on gated CD20$^+$ B/CLL cells (middle contour plot); and (iii) expression of the memory marker, CD27 on gated CD20$^+$ B/CLL cells (bottom contour plot). **B)** Scatter plots with bars show median percentages ± IQR of CD20$^+$ B (if from HDs) or CLL tumour cells (top panel), and median percentages ± IQR of CD86$^+$ (middle panel) and CD27$^+$ (bottom panel) cells, within gated CD20$^+$ B/CLL tumour cells, comparing levels across ibrutinib-treated (n=4) and untreated (n=8) CLL patients and HDs (n=8), *ex-vivo*. Statistical significance was determined after assessing data normality with the Shapiro-Wilk test, by using the one-way ANOVA test or the Kruskal-Wallis test with Dunnett or Dunn's multiple comparison correction tests for parametric or non-parametric data, respectively. *P≤0.05; **P≤0.005; ***P≤0.0005.

A

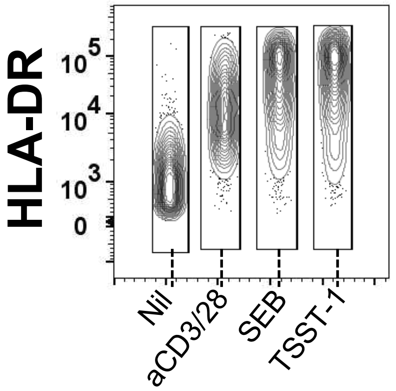

HLA-DR concatenated MFIs		
**Sample**	Mean	SD
**Nil**	1867	253
**αCD3/28**	24821	144
**SEB**	57983	100
**TSST-1**	65140	96.9

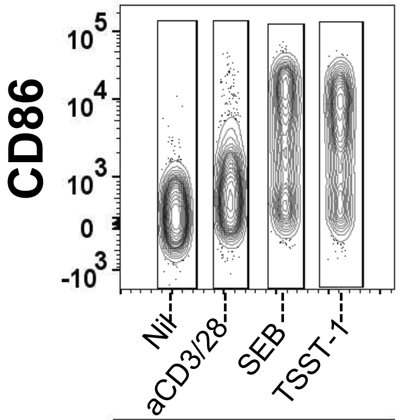

CD86 concatenated MFIs		
**Sample**	Mean	SD
**Nil**	243	230
**αCD3/28**	1250	252
**SEB**	7479	129
**TSST-1**	6371	122

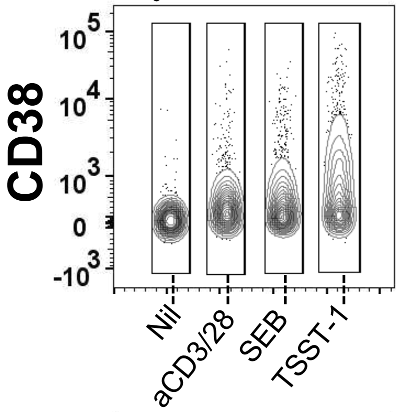

CD38 concatenated MFIs		
**Sample**	Mean	SD
**Nil**	79.8	299
**αCD3/28**	465	386
**SEB**	632	399
**TSST-1**	1605	310

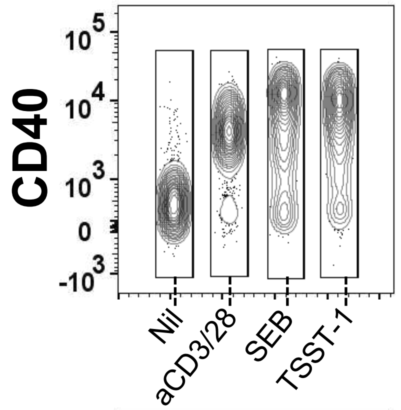

CD40 concatenated MFIs		
**Sample**	Mean	SD
**Nil**	566	141
**αCD3/28**	4463	68.8
**SEB**	8388	87.8
**TSST-1**	8199	82.8

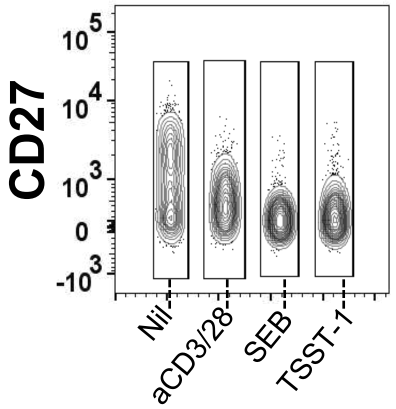

CD27 concatenated MFIs		
**Sample**	Mean	SD
**Nil**	1508	114
**αCD3/28**	556	98.1
**SEB**	186	183
**TSST-1**	271	171

Supplementary Figure 17. Concatenated MFI analyses reveal that exposure to staphylococcal SAgs potentiates the pro-inflammatory phenotype of CLL tumour cells, *in vitro*. PBMCs of CLL patients were stimulated *in vitro*, as described in Supplementary Figure 3 and the Materials and Methods. At d5, cells were harvested, and surface-stained with anti-CD20, anti-HLA-DR, anti-CD86, anti-CD38, anti-CD40, and anti-CD27 mAbs. Viable CLL cells were identified as shown in Supplementary Figure 1D-F. After acquisition, for each experimental culture condition, 4450 events from individual CLL patient samples (n=10) were concatenated using the FlowJO™ version 10. Thereafter, a concatenated file of the four concatenated culture conditions was created and further analysed in comparison to statistically pooled data shown in Figure 7A. **A**) Concatenation flow cytometry plots of CLL patients show the concatenated MFIs of HLA-DR, CD86, CD38, CD40 and CD27 (in order, from top to bottom) in total $CD20^+$ CLL cells, for each culture condition tested: αCD3/28 (blue; a T-cell activator, hence promoting activation of T-helper cells that support B cells), SEB (yellow) and TSST-1 (red) stimulation compared to unstimulated control cultures (Nil; green). The colour-coded tables show the mean and SD of each concatenated parameter for the stimulations mentioned above. Violet shades indicate low to high levels of expression, respectively from light to dark tones.

A

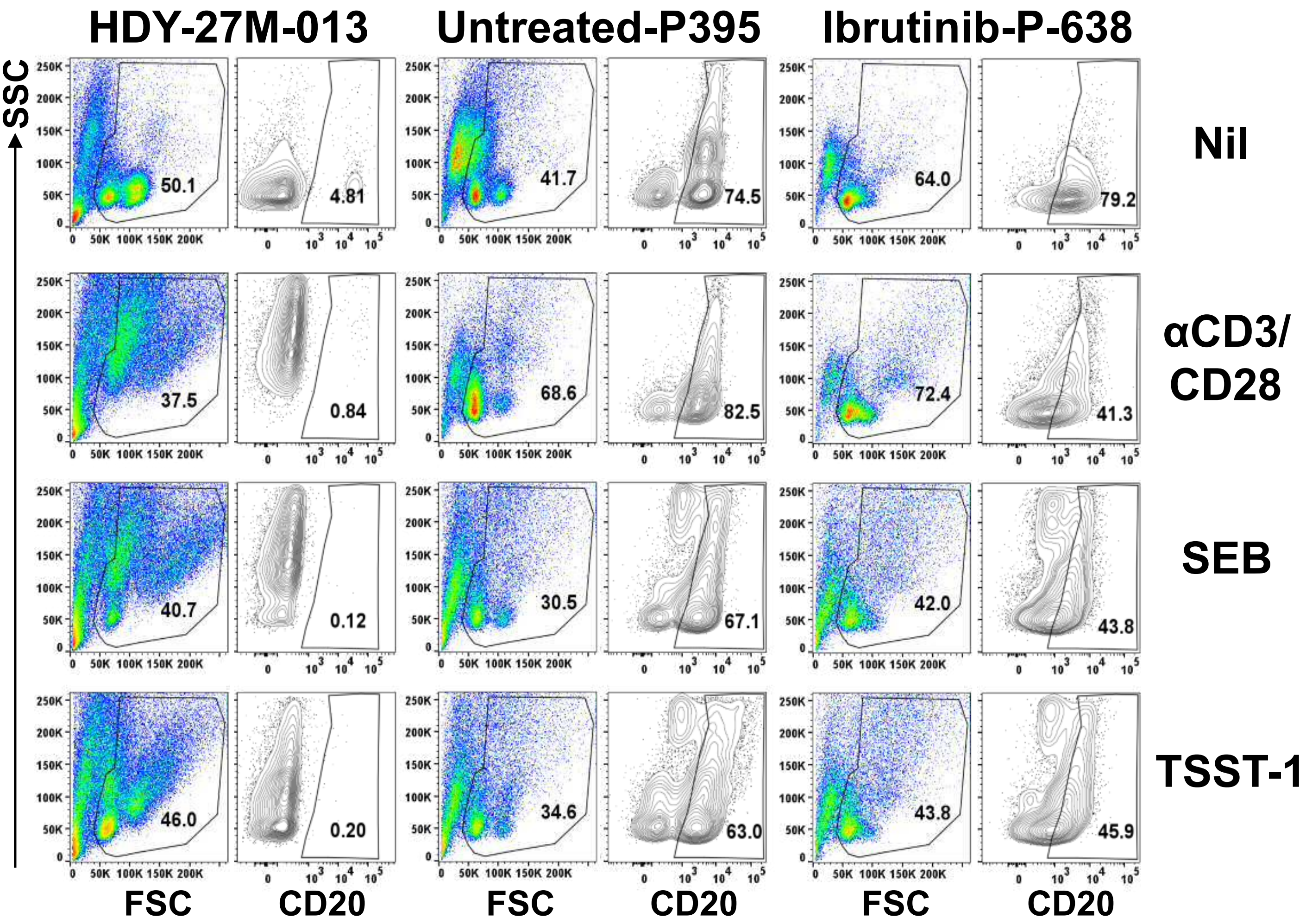
HDY-27M-013
Untreated-P395
Ibrutinib-P-638
Nil
αCD3/
CD28
SEB
TSST-1
SSC
FSC
CD20
50.1
4.81
41.7
74.5
64.0
79.2
37.5
0.84
68.6
82.5
72.4
41.3
40.7
0.12
30.5
67.1
42.0
43.8
46.0
0.20
34.6
63.0
43.8
45.9

Supplementary Figure 18. CD20$^+$ CLL-B cells of CLL patients resist more in cultures with T-cell stimulants than B cells from HDs. Freshly isolated PBMCs (2 x10^6 per condition) from HDs and untreated and ibrutinib-treated CLL patients were stimulated in 24-well plates, using stimulatory antibodies: αCD3 (0.125μg/ml) and αCD28 (2μg/ml) or staphylococcal SAgs (SEB and TSST-1; at 2.5μg/ml) or left unstimulated (Nil) for five days. On day 5, cultured cells were harvested and surface stained with the monoclonal antibody anti-CD20. Viable B and CLL-B cells were identified using the gating strategy described in Supplementary Figure 1D-F. **A**) Flow cytometric analysis shows the lymphocyte/CLL cell gate (FSC *vs.* SSC) and the gate for the detection of CD20+ B/CLL cells (CD20$^+$ vs. SSC) eventually surviving/activating in the cultures. Example plots from HDs (HDY-27M-013; left columns), untreated (P395; middle columns), and Ibrutinib-treated (P638; right columns) CLL patients are provided. HD B cells do not benefit from the exposure to staphylococcal SAgs or αCD3/28 and do not survive in culture with these stimulants, significantly. In contrast, CLL cells (especially when derived from untreated patients) well survive in the cultures with staphylococcal SAgs/αCD3/28 and go on to activate (as shown in Supplementary Figure 17 and Figure 7A-B). Statistical analysis of this dataset is provided in Figure 7C.
